# Modulating Electrostatic Properties and Noncovalent Interactions via Structural Isomerism: The Microwave Spectra and Molecular Structures of (*E*)- and (*Z*)-1,2,3,3,3-Pentafluoropropene and Their Gas-Phase Heterodimers with the Argon Atom

**DOI:** 10.1021/acs.jpca.4c05449

**Published:** 2024-10-02

**Authors:** Helen O. Leung, Mark D. Marshall, Kazuki M. Tayama, Maximillian D. Hauschildt, Elizabeth A. Rose

**Affiliations:** Department of Chemistry, Amherst College, P.O. Box 5000, Amherst, Massachusetts 01002-5000, United States

## Abstract

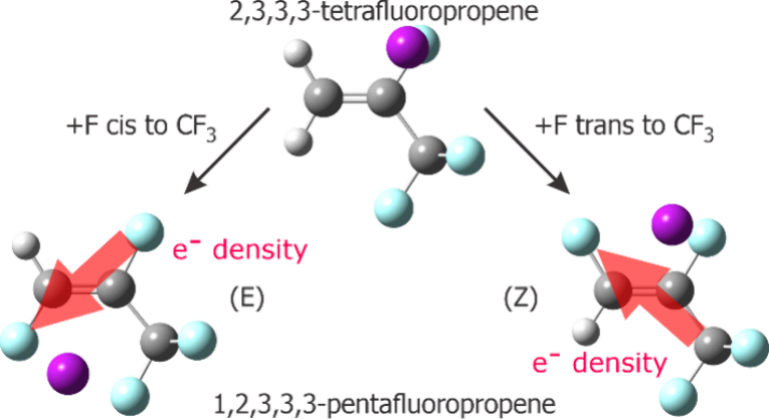

Microwave spectra of both the *E* and *Z* isomers of 1,2,3,3,3-pentafluoropropene along with all three of the singly substituted ^13^C isotopologues for each are obtained using broadband chirped-pulse Fourier transform microwave spectroscopy from 2.0–18.1 GHz. Associated quantum chemistry calculations show that the barrier to internal rotation of the CF_3_ group is significantly higher for the *Z* isomer, which is stabilized by an intramolecular hydrogen bond, although the barriers in both isomers are sufficiently high to prevent the observation of any effects due to internal rotation. The normal isotopologues of the argon heterodimers for both isomers are also observed in the broadband spectrum and a Balle-Flygare cavity Fourier transform microwave spectrometer is used to obtain the 5.0–20.6 GHz spectra of the corresponding ^13^C isotopologues. In each case, the argon atom locates so as to maximize its interactions with areas of significant electron density. However, mapped electrostatic potential surfaces indicate that the areas of greatest nucleophilicity are different for the two isomers, suggesting that they may interact differently in forming heterodimers with protic acids.

## I Introduction

**Figure 1:**
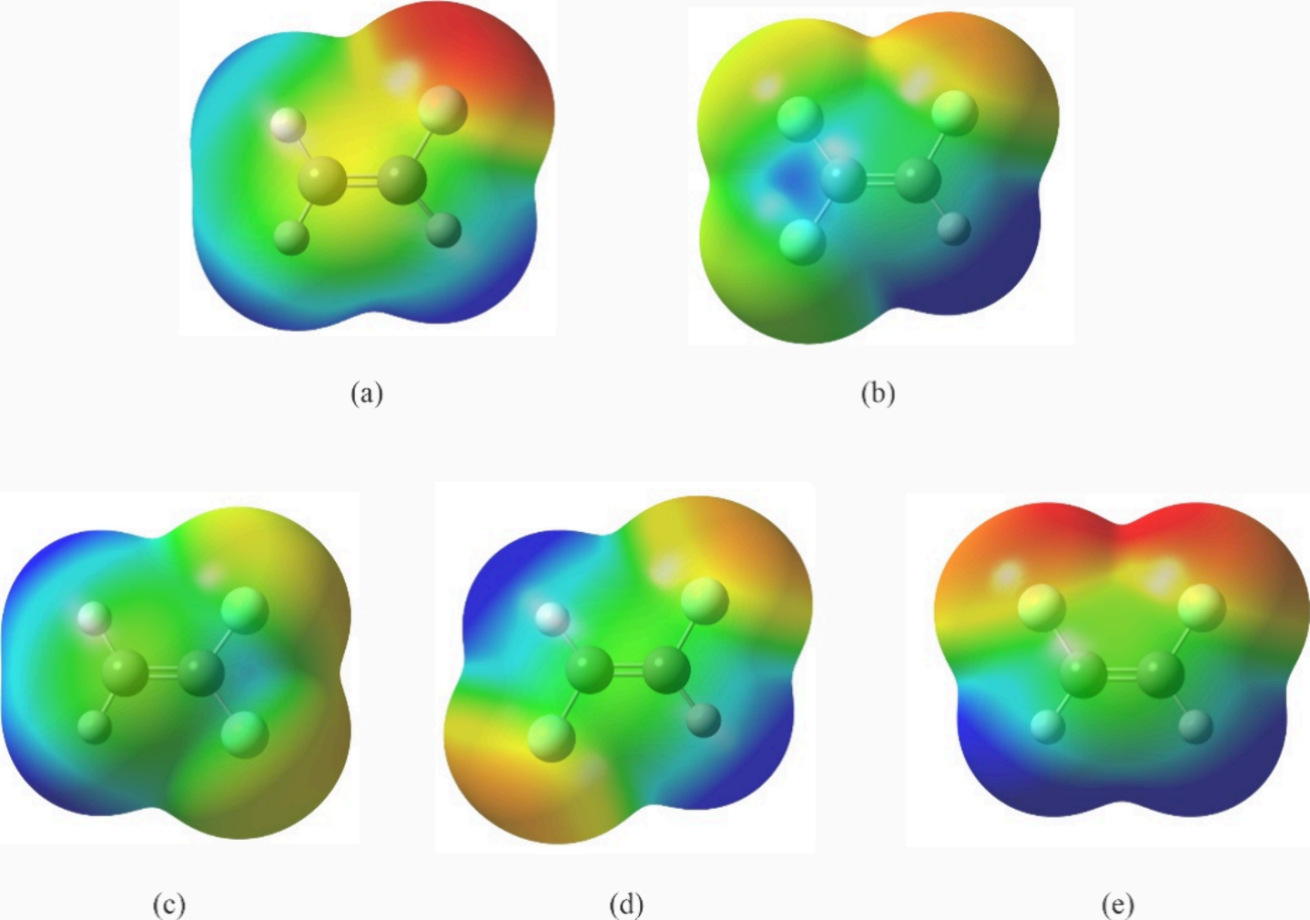
Electrostatic potential surface mapped onto a total electron isosurface for (a) vinyl fluoride, (b) 1,1,2-trifluoroethylene, (c) 1,1-difluoroethylene, (d) *trans*-1,2-difluoroethylene, and (e) *cis*-1,2-difluoroethylene. The same value of electron density is used for the isosurface in all molecules and identical color scales are used. Blue color represents positive electrostatic potential and red, negative electrostatic potential.

Using electron withdrawing halogens, we have been able to tune the properties of ethylenes and observe how they affect the manner in which the substituted molecules participate in intermolecular interactions. The type and the number of halogen atoms naturally play a significant role in affecting the electron density distribution of a haloethylene.^1^ With fluoroethylenes as examples, the effect of different numbers of F atoms can be seen qualitatively by mapping the electrostatic potential of each fluoroethylene onto its total electron density surface at the MP2/6-311++G(2d,2p) level. Specifically, the F atom in vinyl fluoride (Figure 1a) is more nucleophilic than those in 1,1,2-trifluoroethylene (Figure 1b). Among the three H atoms in vinyl fluoride, the one geminal to the F atom is closest to it, and thus can more readily donate its electron density, making it the most electropositive atom in the molecule. In the presence of three F atoms in 1,1,2-trifluoroethylene, the only H atom becomes even more positive than the H atom geminal to the F atom in vinyl fluoride. Additionally, its proximity to the F geminal to it in trifluoroethylene also allows this F atom to be the most nucleophilic atom in the molecule. When two F substituents are present, we have three isomers. The mapped electrostatic potential surfaces of these difluoroethylenes illustrate that the location of the F atoms have considerable impact on the electron density distribution in the molecule. In 1,1-difluoroethylene (Figure 1c), each F atom is three bonds away from one of the two H atoms, but in *trans*- and *cis-*1,2-difluoroethylene (Figure 1d and e), each F atom is three bonds away from one H atom and two bonds away from the other. The H atom two bonds away allows the F atom to more effectively withdraw electron density from it. Thus, the F atoms are more nucleophilic and the H atoms are more electropositive in *cis-* and *trans*-1,2-difluoroethylene than their counterparts in 1,1-difluoroethylene. It is particularly interesting to see that, according to theory, the F atoms are more nucleophilic and the H atoms are more electropositive in *cis*-1,2-difluoroethylene than those in *trans*-1,2-difluoroethylene. This could be an outcome of the “cis effect”: even though the proximity of the electronegative F atoms in *cis*-1,2-difluoroethylene appears to be destabilizing, it is in fact lower in energy than *trans*-1,2-difluoroethylene,^2^ and many theoretical studies (as cited by ref (3)) have attempted to explain its origin. The average structural parameters for the two isomers are slightly different,^4^ and their mapped electrostatic potential surfaces in Figure 1 are generated using these parameters. Briefly, the C–F bond is shorter while the C–H bond is longer in the *cis* isomer than those in the *trans* isomer; the HCC angle is smaller but the FCC angle is greater in the *cis* isomer than those in the *trans* isomer; the C=C bond lengths are, to within 2σ of the larger uncertainty, the same.

The features from the mapped electrostatic surfaces are generally supported by experimental intermolecular geometric parameters of fluoroethylene-protic acid complexes. For example, the protic acid HF binds to an H, F pair located *cis* to each other in vinyl fluoride, *trans-*1,2-difluoroethylene, and 1,1-difluoroethylene: the acid forms a hydrogen bond with the F atom, which bends from linearity so that F in HF can forge a secondary interaction with the H atom in the fluoroethylene. Because the binding mode is the same, the hydrogen bond lengths can be taken as an indicator of the nucleophilicity of the F atoms in the fluoroethylenes that participate in the intermolecular interaction, and they are, respectively, 1.892(14) Å,^5^ 1.910297(68) Å,^6^ and 1.98833(44) Å,^7^ for the HF complexes of vinyl fluoride, *trans-*1,2-difluoroethylene, and 1,1-difluoroethylene, implying a decrease in the nucleophilicity of the F atoms in this series. Similar trends can be observed when the protic acid binding partners are HCl^8−11^ and HCCH.^12,13^ (The complex between *trans*-1,2-difluoroethylene and acetylene is expected to have too small a dipole moment for its rotational spectrum to be readily observed). The three protic acids, HF, HCl, and HCCH, also bind to 1,1,2-trifluoroethylene through an H, F pair, but this time, the paired atoms are geminal, not *cis* to each other. Therefore, in addition to electrostatic factors, steric effects play a role in the structures of the weakly bound complexes. Nevertheless, the large difference in the nucleophilicity of the hydrogen bonded F atoms in 1,1,2-trifluoroethylene and in vinyl fluoride does lead to the expected outcome, as suggested by Figure 1: the hydrogen bonds in the HF, HCl, and HCCH complexes of 1,1,2-trifluoroethylene^14−16^ are longer than their vinyl fluoride counterparts. It is, however, difficult to use structural parameters to compare the nucleophilicity of the F atoms in *cis*- and *trans-*1,2-difluoroethylene due to the lack of appropriate complexes. The HF complex of the *cis* species has not yet been observed, and neither has the HCCH complex of the *trans* species. Their HCl complexes have different motifs: HCl binds to an H, F pair across the double bond of *trans*-1,2-difluoroethylene, but H in HCl is the only atom that interacts with the two F atoms in *cis*-1,2-difluoroethylene;^17^ consequently, the geometric parameters cannot be compared meaningfully. Nevertheless, the HCCH complexes of *cis*-1,2-difluoroethylene and 1,1,2-trifluoroethylene do have the same motif: the binding is through the geminal H, F pair. The hydrogen bond length of HCCH-*cis*-1,2-difluoroethylene is 2.6455(92) Å,^18^ which is shorter than that of HCCH-1,1,2-trifluoroethylene of 2.748(15),^16^ indicating that the F atom involved in the intermolecular interaction in the disubstituted species is more nucleophilic than that in the trisubstituted species, which is consistent with Figure 1.

To further our understanding of how the location of substituents in an olefin can affect intermolecular interactions, we have begun to examine halopropene systems systematically. The mapped electrostatic potential surfaces [also at the MP2/6-311++G(2d,2p) level] of (*E*)-1,3,3,3-tetrafluoropropene^19^ and 2,3,3,3-tetrafluoropropene^20^ show that the relative locations of the CF_3_ group and the F atom bonded to the ethylenic C atoms greatly affect the electronic distributions in these molecules. The F atoms of the CF_3_ group are more nucleophilic in (*E*)-1,3,3,3-tetrafluoropropene than in 2,3,3,3-tetrafluoropropene, but the opposite is true for the F atom bonded to the ethylenic C. This suggests that the CF_3_ group can withdraw electron density more readily, perhaps due to hyperconjugation, from an F atom located *trans* instead of geminal to it. The two rotamers of 2,3,3-trifluoropropene also show electronic distributions that depend on the orientations of the H and F atoms in CF_2_H group.^21^ The present study of (*E*)- and (*Z*)-1,2,3,3,3-pentafluoroethylene extends the knowledge of how electron density is modulated by the location of substituents. These two molecules have identical substituents. One of the ethylenic C atoms, C1, is connected to H and F, while the other C atom, C2, is connected to F and CF_3_. The F atom connected to C1 is *trans* to the F atom and *cis* to the CF_3_ group bonded to C2 in (*E*)-1,2,3,3,3-pentafluoropropene, but it is *cis* to the F atom and *trans* to the CF_3_ group bonded to C2 in (*Z*)-1,2,3,3,3-pentafluoropropene. By characterizing the structures of these molecules and their argon complexes, we are setting the stage to study the similarities and differences in how they form intermolecular interactions with protic acids, which we will generate using argon as a carrier gas.

## II *Ab Initio* Calculations

We use theory both to facilitate our experimental work and to gain insight into the molecular systems. A theoretical study of the barriers of CF_3_ or CH_3_ internal rotation in several propenes containing additional fluorine substituents, including (*E*)- and (*Z*)-1,2,3,3,3-pentafluoropropene, has been reported previously.^3^ To examine the fluorine “cis effect”, the strengths of the π bonds, and the intramolecular interactions in several propenes, that work employs computations conducted at higher levels than we typically use. We will, however, briefly report below our work using the level of theory that we commonly employ, MP2/6-311++G(2d,2p), that has guided our experiments adequately in the past. Additionally, it would be meaningful to be able to compare our computation results here with those performed previously using the same level of theory.

**Figure 2:**
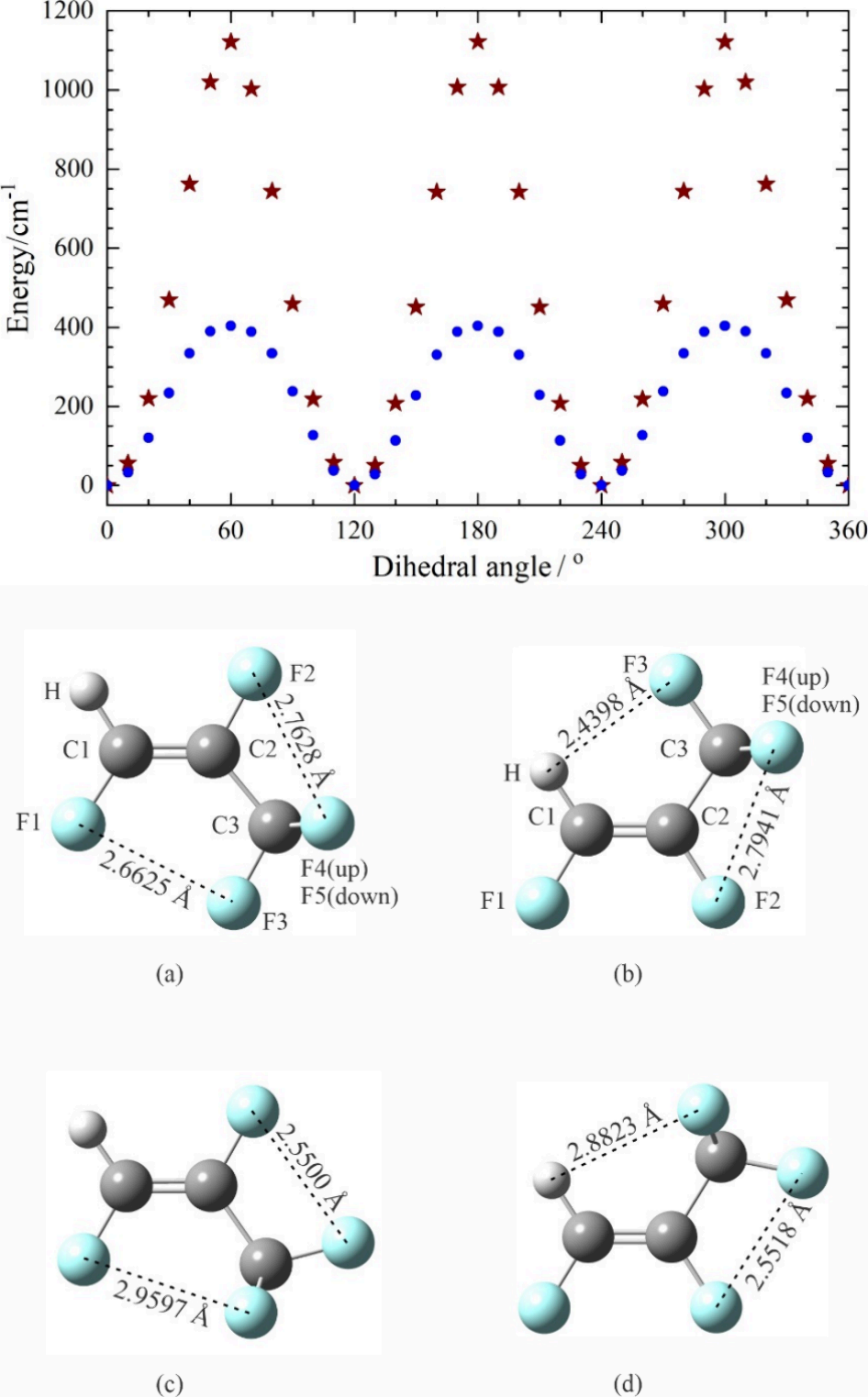
Relaxed scans of the dihedral angle formed by one of the trifluoromethyl F atoms, C3, C2, and C1 in (*E*)- (blue circles) and (*Z*)-1,2,3,3,3-pentafluoropropene (brown stars). The optimized global minimum structures, and the corresponding atom labeling schemes, are shown respectively in (a) and (b); the energy of each is taken to be 0 for the corresponding scan. The optimized structures of the molecules at the potential maxima are shown in (c) and (d), respectively. Atom colors: C, dark gray; H, light gray; F, light blue.

We use GAUSSIAN 16^22^ to investigate the barrier of CF_3_ internal rotation in each of (*E*)- and (*Z*)-1,2,3,3,3-pentafluoropropene by carrying out a relaxed scan of the dihedral angle formed by one of the F atoms in the CF_3_ group, C3, C2, and C1 from 0° to 360° in steps of 10°, while optimizing all other parameters in each step. The labeling scheme for the atoms in each pentafluoropropene, as well as the relative potential energy as a function of the dihedral angle, can be found in Figure 2 (basically, the C atoms and the F atoms attached to them bear the same number; F4 and F5 are the out-of-plane F atoms). The symmetry of the CF_3_ group results in three identical potential minima, and the corresponding optimized structures (with no restriction on the F–C3–C2–C1 dihedral angle) are given in Figure 2a and Figure 2b for (*E*)- and (*Z*)-1,2,3,3,3-pentafluoropropene. The structural parameters of these molecules are listed in Table 1, and the atomic positions in the principal axis systems are available as Supporting Information. These molecules have a plane of symmetry: all but two atoms are in the same plane while two of the trifluoromethyl F atoms are out of the plane. Additionally, the planar trifluoromethyl F atom in each molecule forms a dihedral angle of 0° with C3, C2, and C1, so that it is closest to the atom located *cis* to C3, that is, F1 and H, respectively, in the *E* and *Z* isomer. The rotational constants of the global minimum structures of the two isomers and their dipole moment components are listed in Table 2. We expect both *a* and *b* type rotational transitions from each isomer. Because the *a* component of the dipole moment in the *E* isomer is 1.8 times greater than the *b* component, *a* type transitions should be much stronger. On the other hand, the *b* component of the dipole moment in the *Z* isomer 2.9 times greater than the *a* component; thus, *b* type transitions should be considerably stronger. No *c* type transitions are expected.

**Table 1:** Structural Parameters for (*E*)- and (*Z*)-1,2,3,3,3-Pentafluoropropene Obtained Using *Ab Initio* Calculations^a^ and from a Structure Fit to the Moments of Inertia of Four Isotopologues of Each Molecule^b^

	(*E*)-CHFCFCF_3_		(*Z*)-CHFCFCF_3_	
	theory	experiment	theory	experiment
C1–C2/Å	1.3306	1.3533(73)	1.3293	1.3454(36)
C1–H/Å	1.0777	[1.0777]	1.0748	[1.0748]
C1–F1/Å	1.3335	[1.3335]	1.3335	1.3534(93)
C2–F2/Å	1.3448	[1.3448]	1.3352	[1.3352]
C2–C3/Å	1.4960	1.4775(50)	1.4933	1.4612(51)
C3–F3/Å	1.3330	1.3166(82)	1.3413	[1.3413]
C3–F4/Å	1.3421	[1.3421]	1.3404	[1.3404]
C3–F5/Å	1.3421	[1.3421]	1.3404	[1.3404]
∠HC1C2/°	121.76	[121.76]	123.23	[123.23]
∠F1C1C2/°	123.20	122.54(24)	121.08	[121.08]
∠F2C2C1/°	118.28	117.07(44)	122.59	121.23(49)
∠C3C2C1/°	129.57	129.06(46)	123.82	124.412(85)
∠F3C3C2/°	111.72	112.0355(41)	110.81	111.1591(28)
∠F4C3C2/°	110.37	110.6864^c^	111.01	111.3572^d^
∠F5C3C2/°	110.37	110.6864^c^	111.01	111.3572^d^
∠F4C3C2C1/°	120.78	[120.78]	120.35	[120.35]
∠F5C3C2C1/°	–120.78	[−120.78]	–120.35	[−120.35]

a Except for F4 and F5, all atoms are planar in the theoretical equilibrium structure of each propene and are fixed as such for the experimental average structure.

b 1σ standard deviations in the parameters are given in parentheses. The parameters placed in square brackets are fixed to the *ab initio* values.

c Because of symmetry, the bond angles, ∠F4C3C2 and ∠F5C3C2, are restricted to have the same value, and to be 1.3492° smaller than the ∠F3C3C2 angle, as given by ab initio calculation.

d Because of symmetry, the bond angles, ∠F4C3C2 and ∠F5C3C2, are restricted to have the same value, and to be 0.1981° greater than the ∠F3C3C2 angle, as given by ab initio calculation.

**Table 2:** Rotational Constants and Dipole Moment Components for (*E*)- and (*Z*)-1,2,3,3,3-Pentafluoropropene and Their Argon Complexes Obtained from *Ab Initio* Calculations at the MP2/6-311++G(2d,2p) Level

	(*E*)-CHFCFCF_3_	(*Z*)-CHFCFCF_3_	Ar–(*E*)-CHFCFCF_3_	Ar–(*Z*)-CHFCFCF_3_
*A*/MHz	2726	3462	1509	1435
*B*/MHz	1821	1412	832	803
*C*/MHz	1351	1217	729	640
|*μ*_*a*_|/D	1.78	0.54	0.51	0.03
|*μ*_*b*_|/D	0.99	1.55	1.89	0.73
|*μ*_*c*_|/D	0.00	0.00	0.59	1.43

Each potential energy curve in Figure 2 is plotted using the global minimum energy as a reference, which, for the *Z* isomer, is 1075 cm^–1^ (12.86 kJ mol^–1^) lower than that for the *E* isomer. The rotational barrier for the *E* isomer is 403 cm^–1^ (4.82 kJ mol^–1^), much lower than the 1122 cm^–1^ (13.42 kJ mol^–1^) for the *Z* isomer. These barriers agree well with the higher level calculation using the complete basis set limit,^3^ which are 1.14 and 3.05 kcal mol^–1^ (4.77 and 12.8 kJ mol^–1^) for the *E* and *Z* isomers, respectively. The *E* isomer suffers a repulsion between F1 and F3 while the *Z* isomer is stabilized by a very short intramolecular hydrogen bond of 2.4398 Å (Figure 2). With two F atoms located *cis* to each other in the *Z* isomer, Reference (3) points out that it is also stabilized by the *cis* effect. Additionally, the F–F repulsion causes the C3–C2–C1 angle in the *E* isomer to be greater than that in the *Z* isomer (129.57° vs 123.82° for the *E* and *Z* isomers, respectively, at the present level of theory). The structures at the potential maxima are shown in Figure 2c and 2d for the *E* and *Z* isomers, respectively. These configurations also have a plane of symmetry. Even though the distance between F1 and the two nonplanar F atoms in the CF_3_ group is very long (2.9597 Å) in the global maximum structure of the *E* isomer, the short distance between F2 and the planar F atom in the CF_3_ group is 2.5500 Å, making this configuration too destabilizing. In the global maximum structure of the *Z* isomer, each of the bifurcated hydrogen bonds are too long (2.8823 Å) to be significantly stabilizing, and at the same time, at a distance of (2.5518 Å), there is repulsive interaction between F2 and the planar F in the CF_3_ group.

**Figure 3:**
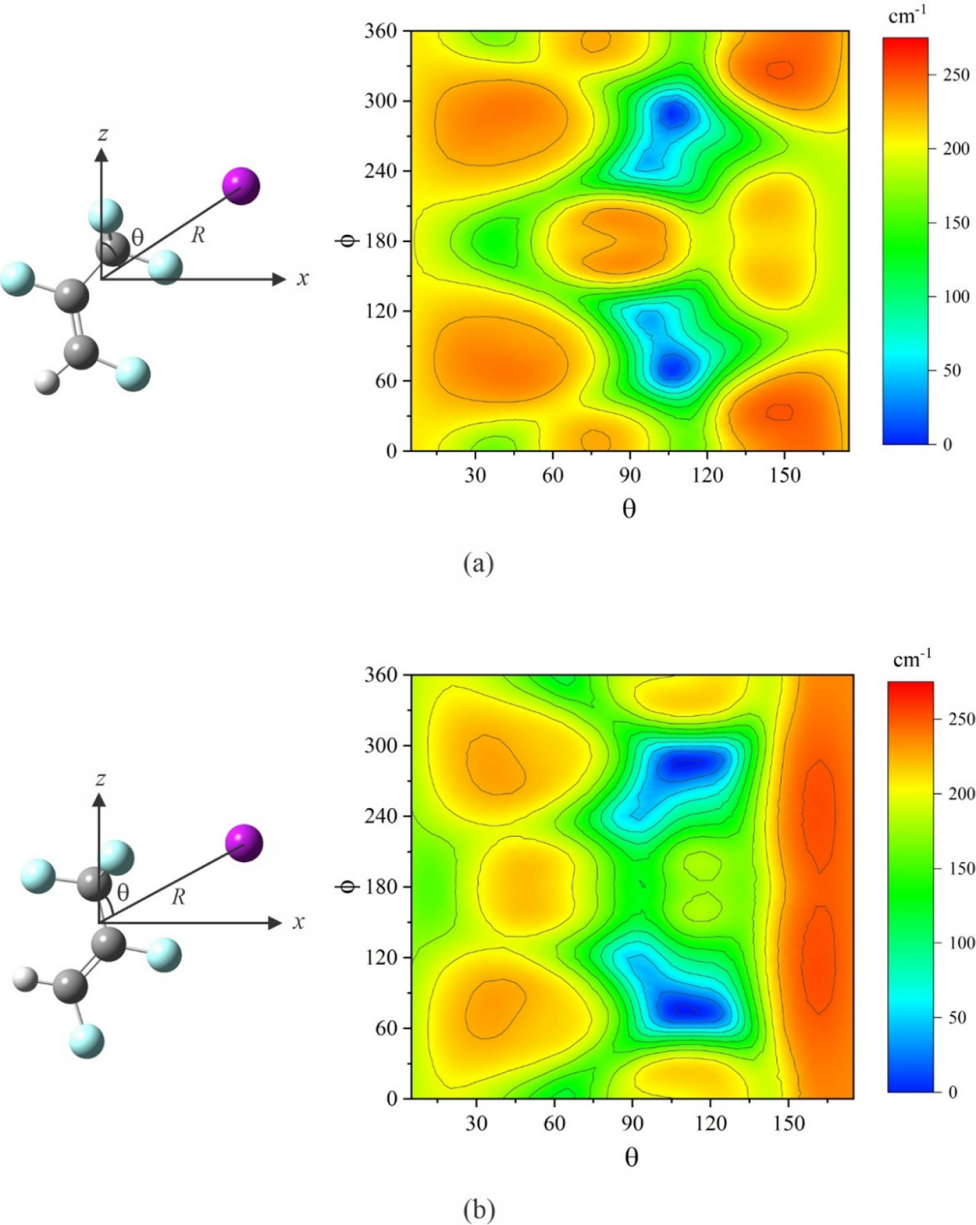
Interaction potential energy contour diagrams formed by argon with (a) (*E*)-1,2,3,3,3-pentafluoropropene and (b) (*Z*)-1,2,3,3,3-pentafluoropropene. *R* is distance between argon and the center of mass of the propene; it is optimized at each pair of values for its polar and azimuthal angles (θ and ϕ, the latter not shown). Atom colors: C, dark gray; H, light gray; F, light blue; Ar, purple.

**Figure 4:**
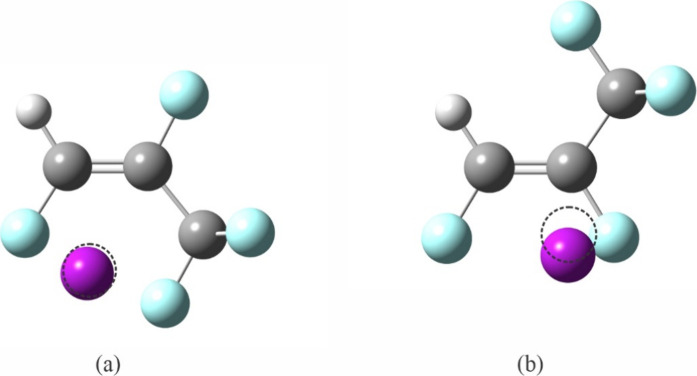
Structures of the complexes formed by argon with (a) (*E*)-1,2,3,3,3-pentafluoropropene and (b) (*Z*)-1,2,3,3,3-pentafluoropropene. All atoms in each complex, except two F atoms and Ar, are in the plane of the page. The theoretical position of Ar is outlined with a dashed circle, whereas the experimental position of Ar is indicated by the purple sphere. Atom colors: C, dark gray; H, light gray; F, light blue; Ar, purple.

Using the experimental, average structure for each of the two isomers of 1,2,3,3,3-pentafluoropropene described in Section IV, we investigate how each interacts with argon. We once again use *ab initio* calculations at the MP2/6-311++G(2d,2p) level. Designating the *a*, *b*, c inertial axes of the propene as the *z*, *x*, *y*, axes, respectively, as conventional in the *I*^*r*^ representation, we place argon at various polar (θ) and azimuthal angles (ϕ) (Figure 3) and optimize its distance from the center of mass of the propene. The relaxed scan is carried out by varying θ from 5° to 175° and ϕ from 0° to 360°, each in 10° increments. The potential energy contour diagrams for the isomers are shown in Figure 3. In each case, there are two equivalent minima, representing argon lying on two different sides of the propene symmetry plane. Upon optimizing, we arrive at the global minimum structures given in Figure 4 (only one of the two equivalent minima for each argon complex is shown). The rotational constants and dipole moment components for each complex are listed in Table 2, and the interaction distances between Ar and each atom in the propenes are in Table 3. (The atomic positions in the principal axis system for each species are available as Supporting Information.)

**Table 3:** Interaction Lengths in Ångstroms between Ar and All Atoms in Ar-(*E*)-1,2,3,3,3-Pentafluoropropene and Ar-(*Z*)-1,2,3,3,3-Pentafluoropropene Obtained from *Ab Initio* Calculations and the Experimental Fit to Four Isotopologues of Each Complex^a^^,^^b^

	Ar–(*E*)-CHFCFCF_3_		Ar–(*Z*)-CHFCFCF_3_	
	theory	experiment	theory	experiment
Ar–C1	3.6973	3.73555(15)	3.7767	3.79444(39)
Ar–C2	3.8028	3.85799(11)	3.5446	3.57091(16)
Ar–C3	3.8009	3.85190(8)	4.1689	4.29419(14)
Ar–F1	3.4753	3.48774(16)	3.9250	3.83465(30)
Ar–F2	4.6512	4.71714(21)	3.4540	3.36875(69)
Ar–F3	3.5194	3.54731(17)	4.7884	4.97297(70)
Ar–F4	3.5127	3.57398(15)	3.6740	3.85307(23)
Ar–F5	5.1291	5.17927(13)	5.2952	5.37952(44)
Ar–H	4.2969	4.33496(21)	4.3478	4.43184(86)

a 1σ standard deviations in the parameters are given in parentheses.

b For both the *ab initio* calculations and the experimental fits, the structure of the respective monomer is fixed at the “experimental” average structure presented in Table 1, which as indicated there, includes both fitted and fixed (to *ab initio* values) structural parameters.

Ar is located in the F1–C1–C2–C3 cavity in the *E* isomer, and in the F1–C1–C2–F2 cavity in the *Z* isomer. We can estimate the important intermolecular interactions that Ar makes using the van der Waals radii of 1.88 Å for Ar, 1.47 Å for F, and 1.70 Å for C,^23^ and we arbitrarily take a distance of within 10% of a van der Waals contact as significant. Ar is positioned well to interact with a large number of heavy atoms in the *E* isomer: C1, C2, C3, F1, F3, and F4. In fact, the distance Ar forms with C1 is only 3.3% greater than the Ar–C van der Waals contact, and those Ar forms with F1, F3, and F4 are between 3.7–5.1% longer than the Ar–F van der Waals contact. For the *Z* isomer, Ar interacts with C1, C2, F2, and F4. The interaction is particularly significant with C2 and F2: The Ar–C2 distance is 1.0% shorter while the Ar–F2 distance is 3.1% longer than the respective van der Waals contacts. We expect to be able to observe all three types of transitions for the most abundant isotopologue for each argon complex of the *E* isomer, with *b* type the strongest because the *b* component of the dipole moment is at least 3.2 times greater than the *a* or *c* component. With a very small dipole moment component along the *a* axis (0.03 D), we should be able to observe only *b* and *c* type transitions for the most abundant isotopologue for the argon complex of the *Z* isomer. Additionally, the *c* component of the dipole moment is nearly twice that of the *b* component, so *c* type transitions should be much stronger.

## III Experiment

Two Fourier transform microwave spectrometers are used in this work: a narrowband, Balle-Flygare instrument operating from 5.0 to 20.6 GHz,^24,25^ and a broadband, chirped-pulse instrument. We have recently extended the frequency range of this latter instrument from its previously described 5.6–18.1 GHz^20,25,26^ to 2.0–18.1 GHz primarily by replacing the original microwave horn transmitter and receiver antennas with 2–18 GHz high gain, wideband models (Q-PAR: QWH-SL-2-18-S-HG-R). These have also improved the performance of the instrument throughout its entire frequency range. Additional details regarding the operation of the broadband spectrometer in the new extension to the range are provided below. For both instruments, the sample is 0.67–1% of (*E*)- or (*Z*)-1,2,3,3,3-pentafluoropropene in argon; it is introduced into the broadband chirped pulse Fourier transform microwave spectrometer via two pulsed valves of 0.8 mm diameter at a backing pressure of 2 atm and into the narrowband, Balle-Flygare spectrometer via one pulsed valve (also 0.8 mm diameter) and a backing pressure of 2–3 atm. The spectrum collected by the broadband instrument allows us to assign transitions due to the most abundant isotopologue and those singly substituted with ^13^C of each propene, as well as the most abundant isotopologue of its argon complex. We then search for and collect transitions arising from the isotopologues of the argon complex singly substituted with ^13^C using the more sensitive narrowband instrument.

**Figure 5:**
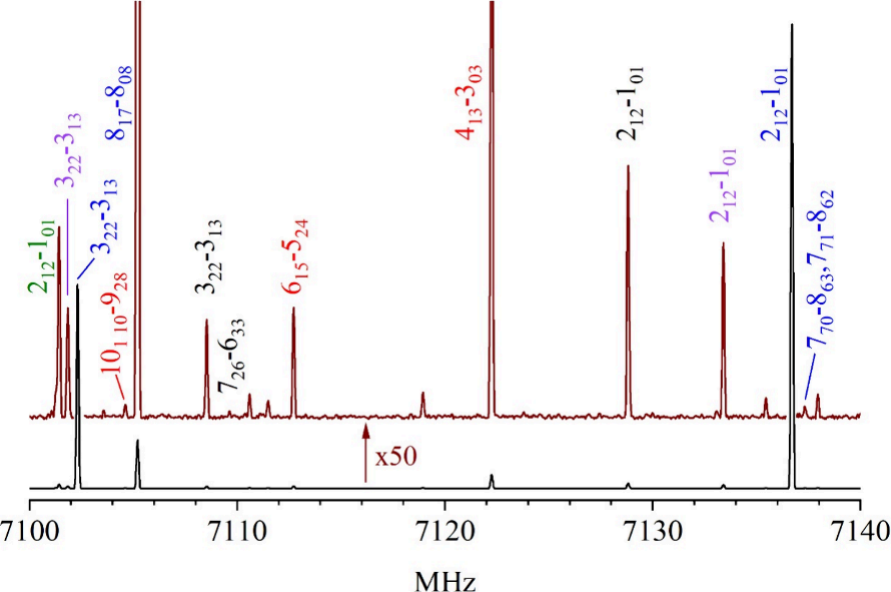
A 40-MHz portion of the broadband spectrum obtained with a sample of (*Z*)-1,2,3,3,3-pentafluoropropene in Ar. The black trace shows strong transitions due to the most abundant isotopologue of the propene. When magnified 50-fold to give the brown trace, transitions due to other species can be readily observed. The transitions labeled in blue, green, purple, and black are due to the most abundant isotopologue of the propene and those singly substituted by ^13^C in the C1, C2, and C3 positions, respectively. Those labeled in red are due to the most abundant species of the argon complex of (*Z*)-1,2,3,3,3-pentafluoropropene.

The broadband spectrum is collected in four segments: 5.6–10.1, 10.1–14.1, 14.1–18.1 GHz, as before, and now also 2.0–6.0 GHz. For the first three bands, the frequencies are obtained by mixing a chirped microwave pulse of 4 μs duration and the appropriate frequency range with the output of phase-locked dielectric resonator oscillators at 10.6, 14.6, and 18.6 GHz, respectively. After isolation of the lower sideband, the pulse is amplified to 20–25 W of power with a solid-state amplifier and polarizes the sample. For 2.0–6.0 GHz operation, a 1.0–3.0 GHz chirped microwave pulse of 1 μs duration is actively doubled (MITEQ: MAX2J010060) and amplified to 500–700 W of power with a traveling wave tube amplifier (Amplifier Research: 500T2G8M53). For both polarization procedures, the subsequent free-induction decay (FID) is digitized at 50 Gs s^–1^ starting 0.5 μs after the end of the excitation pulse and continuing for 20 μs. Ten polarization-digitization cycles are performed for each 800 μs opening of the pulsed valves which operate at 4 Hz, and approximately 1,320,000 to 1,860,000 FIDs are averaged for each segment. The averaged FID is apodized, zero-filled and Fourier transformed, as described previously,^25^ to give a frequency domain spectrum with a resolution element of 11.92 kHz and typical line widths of 125 kHz. The spectra from the four segments are then stitched together. We estimate the frequency measurement uncertainty to be 5–10 kHz using this procedure. As expected given the higher polarization power, the S/N ratio in the 400 MHz overlapping region between 5.6 and 6.0 GHz is significantly better for the data obtained in the 2.0–6.0 GHz segment. A small portion of the spectrum obtained with a sample of (*Z*)-1,2,3,3,3-pentafluoropropene in Ar is shown in Figure 5, showing transitions due to five isotopologues of the propene and the most abundant isotopologue of its argon complex.

The narrowband, Balle-Flygare instrument operates from 5.0 to 20.6 GHz and has been described previously.^24,25^ The background-corrected time domain signals are digitized for 1024 data points and zero-filled to a 2048-point record length before Fourier transformation to give frequency domain signals with a resolution element of 4.8 kHz.

## IV Results

### A Spectral Analysis

#### A1 (*E*)- and (*Z*)-1,2,3,3,3-Pentafluoropropene

As predicted by theory, we are able to observe both *a* and *b* type transitions for both isomers of the pentafluoropropene, with relative intensities as expected. That is, much stronger *a* type transitions are observed for the *E* isomer and much stronger *b* type transitions for the *Z* isomer. No *c* type transitions are observed for either isomer, consistent with the theoretical prediction of the existence of a symmetry plane in each propene. For the *E* isomer, we assign 342 transitions for the most abundant isotopologue, accessing a *J* range of 0–18 and a *K*_*a*_ range of 0–9. The number of transitions for each of the isotopologues singly substituted with ^13^C is between 100–103, with a smaller *J* (0–9) and *K*_*a*_ range (0–5). For the *Z* isomer, we assign 271 transitions over a *J* range of 0–23 and a *K*_*a*_ range of 0–10 but for each of the isotopologues singly substituted with ^13^C, fewer transitions are observed (90–91), with a smaller *J* (0–at least 11) and *K*_*a*_ range (0–4).

The transitions for each species are assigned with the aid of Kisiel’s AABS package^27,28^ and analyzed using the Watson *S*-reduced Hamiltonian in the *I*^*r*^ representation^29,30^ and Pickett’s nonlinear SPFIT program.^31^ We determine, for each isotopologue of the *E* or *Z* isomers, three rotational constants and four to five quartic centrifugal distortion constants. The rms deviation of each fit is 5.7–6.7 kHz (Tables 4 and 5). Tables of observed and calculated transition frequencies with assignments for all isotopologues are provided as Supporting Information.

**Table 4:** Spectroscopic Constants (in MHz, Unless Otherwise Noted) for Four Isotopologues of (*E*)-1,2,3,3,3-Pentafluoropropene^a^

	(*E*)-CHFCFCF_3_	(*E*)-^13^CHFCFCF_3_	(*E*)-CHF^13^CFCF_3_	(*E*)-CHFCF^13^CF_3_
*A*	2731.00041(17)	2728.22310(46)	2724.31012(51)	2729.95696(48)
*B*	1827.29803(13)	1807.73468(37)	1826.25299(39)	1823.97393(37)
*C*	1354.21005(13)	1342.76049(32)	1351.99185(34)	1352.09113(33)
*D*_*J*_/10^–3^	0.3858(12)	0.3497(62)	0.3615(69)	0.3590(62)
*D*_*JK*_/10^–3^	0.42074(93)	0.4082(84)	0.393(10)	0.3904(91)
*D*_*K*_/10^–3^	–0.4494(19)	–0.425(17)	–0.429(20)	–0.418(15)
*d*_1_/10^–3^	–0.05939(12)	–0.0609(16)	–0.0633(18)	–0.0603(14)
*d*_2_/10^–3^	0.092930(56)	0.09007(50)	0.09249(56)	0.09244(49)
no. of rotational transitions	342	100	103	103
no. of *a* type	142	41	44	44
no. of *b* type	200	59	59	59
*J* range	0–18	0–9	0–9	0–9
*K*_*a*_ range	0–9	0–5	0–5	0–5
rms/kHz	6.73	6.12	6.58	6.15

a 1σ standard deviations in the parameters are given in parentheses.

**Table 5:** Spectroscopic Constants (in MHz, Unless Otherwise Noted) for Four Isotopologues of (*Z*)-1,2,3,3,3-Pentafluoropropene^a^^,^^b^

	(*Z*)-CHFCFCF_3_	(*Z*)-^13^CHFCFCF_3_	(*Z*)-CHF^13^CFCF_3_	(*Z*)-CHFCF^13^CF_3_
*A*	3485.49228(22)	3473.52242(42)	3484.60243(47)	3485.40733(39)
*B*	1409.948044(81)	1401.47296(20)	1409.01114(24)	1406.51127(20)
*C*	1217.072158(75)	1209.30374(19)	1216.26989(22)	1214.47153(20)
*D*_*J*_/10^–3^	0.08854(26)	0.0916(22)	0.0923(28)	0.0899(21)
*D*_*JK*_/10^–3^	0.51542(84)	0.5144(72)	0.5099(67)	0.5197(62)
*D*_*K*_/10^–3^	0.0280(18)	[0.0280]	[0.0280]	[0.0280]
*d*_1_/10^–3^	–0.008831(47)	–0.00932(41)	–0.00906(58)	–0.00937(43)
*d*_2_/10^–3^	0.014213(18)	0.01411(16)	0.01421(21)	0.01413(15)
no. of rotational transitions	271	90	91	91
no. of *a* type	48	19	22	19
no. of *b* type	223	71	69	72
*J* range	0–23	0–11	0–13	0–13
*K*_*a*_ range	0–10	0–4	0–4	0–4
rms/kHz	6.17	5.68	6.18	5.72

a 1σ standard deviations in the parameters are given in parentheses.

b Constants that cannot be fitted are fixed to those appropriate to the most abundant isotopologue and enclosed by square brackets.

#### A2 Ar-(*E*)-1,2,3,3,3-Pentafluoropropene and Ar-(*Z*)-1,2,3,3,3-Pentafluoropropene

We assign 472 rotational transitions of *a*, *b*, and *c* type for the most abundant isotopologue of Ar-(*E*)-1,2,3,3,3-pentafluoropropene, and 44–45 rotational transitions, all *b* type, to each of the isotopologues singly substituted with ^13^C. No *a* and *c* type transitions are observed for these minor isotopologues, commensurate with the theoretical prediction that the *b* component of the dipole moment is much greater than the *a* and *c* components. The *J* range is 0–19 and the *K*_*a*_ range is 0–9 for the most abundant species, and these are less for the ^13^C isotopologues: 2–11 for the *J* range and 0–6 for the *K*_*a*_ range (Table 6).

**Table 6:** Spectroscopic Constants (in MHz, Unless Otherwise Noted) for Four Isotopologues of the Ar-(*E*)-1,2,3,3,3-Pentafluoropropene Complex^a^

	Ar–(*E*)-CHFCFCF_3_	Ar–(*E*)-^13^CHFCFCF_3_	Ar–(*E*)-CHF^13^CFCF_3_	Ar–(*E*)-CHFCF^13^CF_3_
*A*	1516.09730(12)	1501.513137(95)	1512.91600(10)	1514.28816(11)
*B*	816.764444(73)	816.73952(16)	815.44282(17)	815.24247(16)
*C*	716.023062(74)	712.744321(51)	714.715967(54)	714.562424(53)
*D*_*J*_/10^–3^	1.16369(41)	1.1519(14)	1.1604(14)	1.1614(14)
*D*_*JK*_/10^–3^	1.88477(60)	1.8878(46)	1.8899(48)	1.8606(47)
*D*_*K*_/10^–3^	–0.0952(14)	–0.1644(35)	–0.1329(36)	–0.0803(35)
*d*_1_/10^–3^	–0.134027(62)	–0.13510(78)	–0.13078(82)	–0.13345(80)
*d*_2_/10^–3^	–0.011205(25)	–0.01210(22)	–0.01110(23)	–0.01087(22)
no. of rotational transitions	472	45	45	44
no. of *a* type	62	0	0	0
no. of *b* type	310	45	45	44
no. of *c* type	100	0	0	0
*J* range	0–19	2–11	2–11	2–11
*K*_*a*_ range	0–9	0–6	0–6	0–6
rms/kHz	6.72	0.91	0.97	0.95

a 1σ standard deviations in the parameters are given in parentheses.

For Ar-(*Z*)-1,2,3,3,3-pentafluoropropene, *b* and *c* type transitions are assigned for all isotopologues: 401 for the most abundant species and 55–57 for each isotopologue singly substituted with ^13^C. No *a* type transitions are observed, consistent with theoretical prediction of a small dipole moment component along the *a* axis. The *J* range of 0–15 and *K*_*a*_ range of 0–9 for the most abundant species are greater than those for the ^13^C isotopologues, which are 2–10 and 0–6, respectively (Table 7).

**Table 7:** Spectroscopic Constants (in MHz, Unless Otherwise Noted) for Four Isotopologues of the Ar-(*Z*)-1,2,3,3,3-Pentafluoropropene Complex^a^^,^^b^

	Ar–(*Z*)-CHFCFCF_3_	Ar–(*Z*)-^13^CHFCFCF_3_	Ar–(*Z*)-CHF^13^CFCF_3_	Ar–(*Z*)-CHFCF^13^CF_3_
*A*	1506.79333(25)	1494.82362(24)	1504.89123(23)	1506.11417(19)
*B*	766.75309(16)	766.32086(11)	766.53924(10)	764.16999(10)
*C*	624.49919(13)	622.62928(10)	624.122896(91)	622.671237(79)
*D*_*J*_/10^–3^	4.7180(16)	4.6916(10)	4.69308(90)	4.71572(96)
*D*_*JK*_/10^–3^	–25.7509(42)	–25.4644(94)	–25.5554(83)	–25.9733(73)
*D*_*K*_/10^–3^	71.2849(75)	70.295(13)	70.901(12)	71.634(10)
*d*_1_/10^–3^	–1.97711(58)	–1.97669(39)	–1.97015(35)	–1.97290(40)
*d*_2_/10^–3^	–0.149384(51)	–0.15000(20)	–0.15015(18)	–0.14836(15)
*H*_*J*_/10^–6^	–0.1777(57)	[–0.1777]	[–0.1777]	[–0.1777]
*H*_*JK*_/10^–6^	3.306(22)	3.38(10)	3.239(91)	3.385(82)
*H*_*KJ*_/10^–6^	–15.565(57)	–15.97(28)	–15.66(25)	–15.77(23)
*H*_*K*_/10^–6^	20.507(69)	20.83(23)	20.55(20)	20.66(18)
*h*_1_/10^–6^	–0.0736(25)	[–0.0736]	[–0.0736]	[–0.0736]
no. of rotational transitions	401	57	55	55
no. of *b* type	180	16	15	14
no. of *c* type	221	41	40	41
*J* range	0–15	2–10	2–10	2–10
*K*_*a*_ range	0–9	0–6	0–6	0–6
rms/kHz	6.80	1.19	1.06	0.90

a 1σ standard deviations in the parameters are given in parentheses.

b Constants that cannot be fitted are fixed to those appropriate to the most abundant isotopologue and enclosed by square brackets.

With the Watson *S*-reduced Hamiltonian in *I*^*r*^ representation^29,30^ and Pickett’s nonlinear SPFIT program,^31^ we determine, for each isotopologue of Ar-(*E*)-1,2,3,3,3-pentafluoropropene, three rotational constants and five quartic centrifugal distortion constants (Table 6). For Ar-(*Z*)-1,2,3,3,3-pentafluoropropene, in addition to the rotational constants and quartic centrifugal distortion constants, we determine 3–5 sextic centrifugal distortion constants for each isotopologue (Table 7). The AABS package^27,28^ is used in the analysis of spectra obtained with the broadband instrument, and the rms deviation for each fit is characteristic of the spectrometer used to collect the spectrum: approximately 7 kHz for the most abundant species (broadband instrument) and 1 kHz for the minor isotopologues (narrowband spectrometer). Once again, tables of observed and calculated transition frequencies with assignments for all isotopologues are provided as Supporting Information.

### B Structure Determination

#### B1 (*E*)- and (*Z*)-1,2,3,3,3-Pentafluoropropene

According to theory, there is a plane of symmetry in the *E* and *Z* isomers. Therefore, the only atoms that contribute to the value of the second moment, *P*_*cc*_, for any isotopologue of each isomer are those that are out-of-plane. Consequently, a substitution of ^13^C for ^12^C should not affect the value of *P*_*cc*_. Indeed, this is the case. The values of *P*_*cc*_ for the 4 isotopologues of the *E* isomer range from 44.212 to 44.217 u Å^2^ and those for the 4 isotopologues of the *Z* isomer range from 44.091 to 44.096 u Å^2^. The very small variation in these values in the same isomer simply reflects slightly different zero-point motions in the isotopologues, and the small difference between the two isomers suggest that the out-of-plane F atoms are arranged somewhat differently (different C–F lengths and/or different out-of-plane angles). The fact that all C atoms lie in the same plane in each isomer is also consistent with a Kraitchman analysis.^32^ The substitution coordinates for the C atoms in the principal axis system of the most abundant isotopologue of each isomer are listed in Table 8. The values of *c* coordinates so obtained for the C atoms are either close to 0 or imaginary, indicating these atoms are all in the *ab* plane.

**Table 8:** Coordinates of the Carbon Atoms in (*E*)- and (*Z*)-1,2,3,3,3-Pentafluoropropene Determined from Structure Fits^a^ and from Kraitchman Analyses^b^

	(*E*)-CHFCFCF_3_		(*Z*)-CHFCFCF_3_	
	*a*/Å	*b*/Å	*a*/Å	*b*/Å
from structure fit				
C1	–1.7318(18)	–0.4503(63)	–1.4738(13)	–0.7125(12)
C2	–0.3981(78)	–0.6798(44)	–0.4865(41)	0.2015(18)
C3	0.7140(21)	0.2930(26)	0.9385(20)	–0.1221(16)
substitution coordinates^c^^,^^d^				
C1	–1.73202(87)	–0.4424(34)	–1.4737(10)	–0.7120(21)
C2	–0.3974(38)	–0.6760(22)	–0.4884(31)	0.1910(79)
C3	0.7147(21)	0.2766(54)	0.9405(16)	–0.0923(16)

a In the equilibrium structure of each molecule, these atoms are in the symmetry plane of the propene, and are fixed to be so in the determination of the average structure. As such, the *c* coordinate of each is 0.

b The values for the *c* substitution coordinates for C1 and C3 are imaginary in each molecule. Those for C2 are near zero with large uncertainties; specifically, they are 0.01(10) Å and 0.027(55) Å, respectively, in the (*E*) and (*Z*) isomers. Taken together, they indicate that the carbon atoms are in the *a*-*b* plane.

c Costain errors^34^ (for the substitution coordinates) or 1σ standard deviations (in the structure fit parameters) are given in parentheses.

d Although only the absolute values of the substitution coordinates can be determined from the Kraitchman analysis, the relative signs are assigned using physically reasonable atomic distances.

The experimental structure of each isomer is then determined by fixing all atoms, except two F atoms in the CF_3_ group, to lie in the same plane. For the *E* isomer, we restrict the values of the F4C3C2 and F5C3C2 angles to be the same, and each being 1.3492° smaller than the value of the F3C3C2 angle, as suggested by theory, and fit three bond lengths (C1–C2, C2–C3, C3–F3) and 4 bond angles (F1C1C2, F2C2C1, C3C2C1, F3C3C2) to the 12 rotational constants of the 4 isotopologues using Kisiel’s STRFIT program.^33^ The rms deviation of the fit is 0.0056 u Å^2^; the structural parameters are listed in Table 1, and the coordinates of each atom are available as Supporting Information. The structural fit for the *Z* isomer is similarly performed. Specifically, we restrict the values of the F4C3C2 and F5C3C2 angles to be the same, and each being 0.1981° greater than the value of the F3C3C2 angle, as suggested by theory, and fit three bond lengths (C1–C2, C1–F1, C2–C3) and 3 bond angles (F2C2C1, C3C2C1, F3C3C2) to the 12 rotational constants of the 4 isotopologues. The rms deviation of the fit is 0.0039 u Å^2^; the structural parameters are also listed in Table 1, and the coordinates of each atom are available as Supporting Information. The structures so determined, henceforth called the “average structures,” for these isomers are the ones used to determine the interaction potential energy contour diagrams with argon, as described in Section II.

The coordinates of the C atoms in these two fits are listed in Table 8. For the *E* isomer, each substitution coordinate agrees excellently with the corresponding average coordinate, differing from the average value by no more than 0.6% in most cases. The greatest differences are for the two values of the *b* coordinate for C1 (1.8%) and those for the *b* coordinate for C3 (5.6%), but they are only 1.3σ and 3.0σ using the higher uncertainty of the two parameters under comparison. Similarly, for the *Z* isomer, each substitution coordinate differs from the average value by no more than 0.4%, although the differences for the values of the *b* coordinates for C2 and those for the *b* coordinates for C3 are greater, 5.2% and 24%, respectively, which correspond to 1.3σ and 18.6σ of the higher uncertainty of the two parameters under comparison. The 18.6σ value represents a difference of 0.0298 Å and indicates that the zero-point motion in the average structure involves C3 to a greater extent than the other C atoms.

#### B2 Ar-(*E*)-1,2,3,3,3-Pentafluoropropene and Ar-(*Z*)-1,2,3,3,3-Pentafluoropropene

Fixing the structures of the *E* and *Z* isomers to their average structures as determined in the previous section, three geometric parameters are required to locate argon in each of the argon complexes. Choosing parameters that are not correlated, for Ar-(*E*)-1,2,3,3,3-pentafluoropropene we fit the distance Ar–F1, the angle Ar–F1–C1, and the dihedral angle Ar–F1–C1–F5, whereas for Ar-(*Z*)-1,2,3,3,3-pentafluoropropene we fit the distance Ar–C1, the angle Ar–C1–C2, and the dihedral angle Ar–C1–C2–F4. The rms deviations of the two fits are, respectively, 0.021 u Å^2^ and 0.064 u Å^2^, respectively. These larger rms deviations indicate that the zero-point motion is more significant in each complex than in the corresponding propene monomer. The distances between Ar and the atoms in each propene are calculated using Kisiel’s EVAL program;^27^ the values are listed in Table 3 and the complexes are displayed graphically in Figure 4.

The average coordinates of each C atom are listed in Table 9, together with the Kraitchman substitution coordinates for the C atoms in the principal axis system of the most abundant isotopologue of each isomer. This time, unlike the case with the propene monomers, the substitution and average coordinates do not agree quite as well. This is certainly expected because of large amplitude motions in weakly bound complexes. For the argon complex of the *E* isomer, they differ by 0.0006–0.098 Å, corresponding to 0.4–16 times the larger uncertainty. The differences are greater for the argon complex of the *Z* isomer: the coordinates differ by 0.0004–0.17 Å, corresponding to 0.4–53 times the larger uncertainty. Thus, Ar-(*Z*)-1,2,3,3,3-pentafluoropropene exhibits larger zero-point motions than Ar-(*E*)-1,2,3,3,3-pentafluoropropene, and this is reflected both in the large magnitudes of the quartic centrifugal constants *D*_*JK*_ and *D*_*K*_ (−25.7 and 71.3 kHz versus 1.9 and −0.1 kHz for the most abundant species of the *Z* and *E* complexes, respectively) and the necessity to use sextic centrifugal constants to fit the transitions in the argon complex of the *Z* isomer (Table 7).

**Table 9:** Coordinates of the Carbon Atoms in the Argon Complexes of (*E*)- and (*Z*)-1,2,3,3,3-Pentafluoropropene Determined from Structure Fits and from Kraitchman Analyses

	Ar–(*E*)-CHFCFCF_3_			Ar–(*Z*)-CHFCFCF_3_		
	*a*/Å	*b*/Å	*c*/Å	*a*/Å	*b*/Å	*c*/Å
from structure fit						
C-1	–0.21600(17)	–1.80368(4)	–0.08713(8)	–0.07061(41)	1.59218(32)	–0.53800(36)
C-2	–0.92011(10)	–0.70494(5)	–0.44566(6)	–0.45950(11)	0.57361(13)	0.25030(2)
C-3	–1.04870(7)	0.58854(4)	0.25678(2)	–1.49400(8)	–0.39591(10)	–0.10334(5)
substitution coordinates^a^^,^^b^						
C-1	–0.118(13)	–1.80023(83)	–0.071(21)	–0.2406(62)	1.54061(97)	–0.5651(27)
C-2	–0.8925(17)	–0.7042(21)	–0.4568(33)	–0.3523(43)	0.6040(25)	0.2455(61)
C-3	–1.0493(14)	0.5873(26)	0.2363(64)	–1.4936(10)	–0.3884(39)	–0.042(36)

a Costain errors^34^ (for the substituion coordinates) or 1σ standard deviations (in the structure fit parameters) are given in parentheses.

b Although only the absolute values of the substitution coordinates can be determined from the Kraitchman analysis, the relative signs are assigned using physically reasonable atomic distances.

## V Discussion

It is remarkable that theoretical equilibrium structures determined from a moderate level of theory [MP2/6-311++G(2d,2p)] agree very well with the experimental, average structures for all the species studied in this work. In fact, the rotational constants of the theoretical structure differ from the experimental values by 0.18–0.34% (3–6 MHz) for (*E*)-1,2,3,3,3-pentafluoropropene, and by 0.01–0.67% (0–23 MHz) for (*Z*)-1,2,3,3,3-pentafluoropropene. These differences are, not surprisingly, greater for the weakly bound argon complexes because of their larger amplitude motion: for Ar-(*E*)-1,2,3,3,3-pentafluoropropene, the rotational constants differ by 0.47–1.87% (7–15 MHz) and for Ar-(*Z*)-1,2,3,3,3-pentafluoropropene, 2.48–4.76% (16–72 MHz). Despite the larger differences for the complexes, theory remains an excellent guide to our work, and here, it is apparent that the Ar-(*Z*)-1,2,3,3,3-pentafluoropropene complex exhibits much greater zero point motions than Ar-(*E*)-1,2,3,3,3-pentafluoropropene, as we have surmised by the greater magnitudes of some quartic centrifugal constants and the need to use sextic centrifugal constants to fit the spectrum of each of the isotopologues of the former.

Because the rotational constants of the experimental average structure of each propene agree so well with the theoretical equilibrium structure, the fitted structural parameters do not deviate much form the theoretical values (Table 1). The intramolecular hydrogen bond between H and F3 is 2.4503 Å in the average structure of the *Z* isomer, slightly longer than the theoretical value of 2.4398 Å. Our interest in determining the experimental structures of these propenes ultimately concerns with how they interact with other molecules. Here, using the structureless argon, we can explore how they interact via dispersion forces (Table 3 and Figure 4). The experimental position of argon in the complex with the *E* isomer is very similar to the theoretical structure. The distance between argon and each atom with which it has significant interaction (C1, C2, C3, F1, F2, and F3) is greater in the average structure by 0.012–0.061 Å than in the equilibrium structure. For the *Z* isomer, similar increases are also seen for C1, C2, and F4, by 0.018–0.179 Å. In fact, the Ar–F4 distance is 15% longer than the van der Waals contact, significantly decreasing the importance of this interaction. On the other hand, the Ar–F2 distance in the experimental structure has decreased by 0.085 Å from the equilibrium configuration; with a distance of 3.36875(69) Å, it is just slightly longer than the van der Waals contact of 3.35 Å, which, together with the Ar–C2 interaction [of a length of 3.57091(16) Å, very close to the van der Waals contact of 3.58 Å], constitute the strongest intermolecular interactions in the complex.

**Figure 6:**
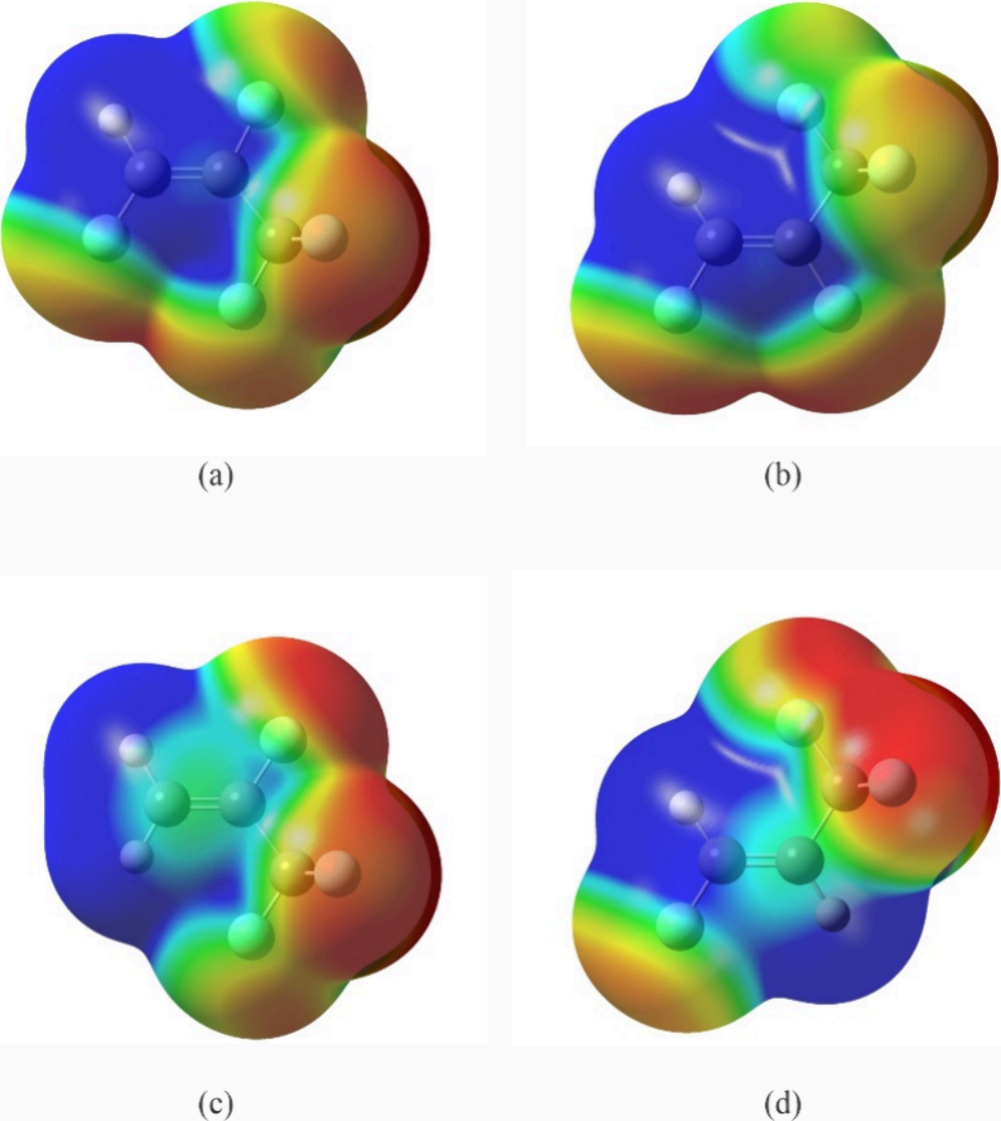
Electrostatic potential surface mapped onto a total electron isosurface for (a) (*E*)-1,2,3,3,3-pentafluoropropene, (b) (*Z*)-1,2,3,3,3-pentafluoropropene, (c) 2,3,3,3-tetrafluoropropene, and (d) (*E*)-1,3,3,3-tetrafluoropropene. The same value of electron density is used for the isosurface in these molecules and identical color scales are used (the color scales are different from those used in Figure 1). Blue color represents positive electrostatic potential and red, negative electrostatic potential.

To understand the electronic density distribution, we map the electrostatic potential onto the total electron density of each of these propenes at the MP2/6-311++G(2d,2p) level (Figure 6a and b). The most nucleophilic atoms for the *E* isomer are the two out-of-plane F atoms, whereas for the *Z* isomer, they are F1 and F2. To rationalize these, we turn to the mapped electrostatic surfaces of 2,3,3,3-tetrafluoropropene and (*E*)-1,3,3,3-tetrafluoropropene (Figure 6c and d). Treating (*E*)-1,2,3,3,3-pentafluoropropene as a molecule resulting from replacing the H atom *trans* to the F atom in 2,3,3,3-tetrafluoropropene with another F atom, we can see the presence of this additional F renders the F atom *trans* to it (F2) less nucleophilic than the F atoms in the CF_3_ group. This is likely a result of the ease of withdrawing or equalizing electron density of the two F substituents through the double bond in the pentafluoropropene. Turning to (*Z*)-1,2,3,3,3-pentafluoropropene, it can be viewed as replacing the H atom *cis* to the F atom either in 2,3,3,3-tetrafluoropropene or in (*E*)-1,3,3,3-tetrafluoropropene. Taking the first view, it appears that the presence of this extra F atom, which is *trans* to the CF_3_ group in 2,3,3,3-tetrafluoropropene, is more effective in removing electron density from that group than from the F atom (F2) located *cis* to it. If we take the second view, then the additional F atom appears to be effectively removing electron density in the geminal CF_3_ group. In either way of looking at it, an additional fluorine atom is drawing electrons from the CF_3_ group that with three fluorine atoms of its own might be expected to be more electronegative. However, through hyperconjugation, a C–F σ bond in CF_3_ can effectively become part of the π system that extends over the olefinic F and C atoms. This would provide a means for electrons in the CF_3_ group to become delocalized via the unoccupied lowest energy π* orbital with the resulting resonance stabilization driving the increased electron density on the additional F atom.

It remains to be seen how the different electron density distributions in (*E*)- and (*Z*)-1,2,3,3,3-pentafluoropropene affect their ability to bind to a protic acid. The only proton in each of these molecules is highly electropositive, so it likely will interact with the nucleophilic portion of the acid. Will the acid form a hydrogen bond with the most nucleophilic F atom in each of these molecules? If not, we will be able to gain information regarding the steric requirements in the interaction.

## VI Conclusions

Microwave spectra for four isotopologues each of (*E*)- and (*Z*)-1,2,3,3,3-pentafluoropropene and of their gas-phase heterodimers with the argon atom have been obtained and analyzed. Significant assistance in identifying and assigning the spectra is provided by the close agreement between the theoretical predictions at the MP2/6-311++G(2d,2p) level of theory of rotational constants (corresponding to equilibrium molecular structures) and the experimentally determined constants (corresponding to ground-state, vibrationally averaged structures) for both the monomeric fluoropropenes and their argon complexes. Similarly, there is close agreement between the predicted and experimentally determined structures.

As could be expected, these structures reveal differences in both intra- and intermolecular noncovalent interactions between the *E* and *Z* isomers of these two pentafluoropropenes. These include a higher barrier to internal rotation of the CF_3_ group in (*Z*)-1,2,3,3,3-pentafluoropropene that can be attributed to the stabilizing effect of an intramolecular hydrogen bond at the minimum energy configuration and the destabilizing effect of fluorine–fluorine repulsion at the top of the barrier. In (*E*)-1,2,3,3,3-pentafluoropropene, there is no opportunity for formation of the hydrogen bond to fluorine atoms in the CF_3_ group, which experiences fluorine–fluorine repulsion for all values of the dihedral angle, and indeed finds a minimum energy configuration with one of its fluorine atoms coplanar with the olefinic fluorine atom on C1 that allows the two other CF_3_ fluorine atoms to be staggered with respect to the fluorine atom on C2. Likewise, in the argon complexes, the argon atom seeks out a location that enhances its ability to interact via dispersion interactions with areas of significant electron density, namely by maximizing its contacts with heavy atoms.

However, the two isomers also display how the location of substituents can modulate the electrostatic properties of the molecule, which can then also impact noncovalent interactions. Most significantly, in (*Z*)-1,2,3,3,3-pentafluoropropene, the fluorine atoms bonded to the olefinic carbons (and consequently coplanar with them) are the two most nucleophilic atoms in the molecule. In contrast, for (*E*)-1,2,3,3,3-pentafluoropropene, the two most nucleophilic atoms are the two (equivalent) out-of-plane fluorine atoms of the CF_3_ group. For both isomers the most electrophilic atom is the lone hydrogen atom, but this is on the same side of the C=C double bond as the CF_3_ group in one isomer and on the opposite side in the other. How these electrostatic and steric effects will be balanced in leading to the preferred structures for the heterodimers with protic acids promises to be a fascinating avenue to explore.

## Supporting Information Available

Tables of the atomic coordinates in the appropriate principal inertial axis system for the structures of (*E*)- and (*Z*)-1,2,3,3,3-pentafluoropropene and their argon complexes, observed transition frequencies with assignments and residuals (obs. - calc.) from the least-squares fits, and the complete citation for Gaussian 16 (ref (22)) (PDF)
