# Supplementary material for: Modulating Electrostatic Properties and Noncovalent Interactions via Structural Isomerism: The Microwave Spectra and Molecular Structures of (E)- and (Z)-1,2,3,3,3-Pentafluoropropene and Their Gas-Phase Heterodimers with the Argon Atom
Source: J Phys Chem A. 2024 Oct 2;128(40):8739–50. doi: 10.1021/acs.jpca.4c05449 (PMC11472316; doi:10.1021/acs.jpca.4c05449)
Supplement: Supplementary file 1 — jp4c05449_si_001.pdf [file jp4c05449_si_001.pdf]

Modulating Electrostatic Properties and Non-covalent Interactions via Structural Isomerism: The Microwave Spectra and Molecular Structures of (*E*)- and (*Z*)-1,2,3,3,3-Pentafluoropropene and Their Gas-phase Heterodimers with the Argon Atom

Helen O. Leung*, Mark D. Marshall*, Kazuki M. Tayama, Maximillian D. Hauschildt, and Elizabeth A. Rose

Department of Chemistry, Amherst College, P.O. Box 5000, Amherst, MA 01002-5000, United States

Supporting Information

Address for correspondence: Prof. Mark D. Marshall
Department of Chemistry
Amherst College
P.O. Box 5000
Amherst, MA 01002-5000
Telephone: (413) 542-2006
Fax: (413) 542-2735
E-mail: mdmarshall@amherst.edu

*Corresponding authors. Fax: +1-413-542-2735; *e-mail addresses:* hleung@amherst.edu (H.O. Leung), mdmarshall@amherst.edu (M.D. Marshall).

Table S1 presents the atomic coordinates in the appropriate principal inertial axis system for the structures of (*E*)- and (*Z*)-1,2,3,3,3-pentafluoropropene and their argon complexes.

Tables S2 through S17 contain the quantum number assignments, observed transition frequencies (in MHz), and the residuals (obs. - calc., also in MHz) from the least squares fits. The quantum numbers are J, K_a, and K_c, the usual asymmetric top rotational quantum numbers for the initial and final levels of the transition.

The complete reference for Gaussian 16 (reference 22) is given here.

Frisch, M. J.; Trucks, G. W.; Schlegel, H. B.; Scuseria, G. E.; Robb, M. A.; Cheeseman, J. R.; Scalmani, G.; Barone, V.; Petersson, G. A.; Nakatsuji, H.; Li, X.; Caricato, M.; Marenich, A. V.; Bloino, J.; Janesko, B. G.; Gomperts, R.; Mennucci, B.; Hratchian, H. P.; Ortiz, J. V.; Izmaylov, A. F.; Sonnenberg, J. L.; Williams-Young, F.; Ding, F.; Lipparini, F.; Egidi, F.; Goings, J.; Peng, B.; Petrone, A.; Henderson, T.; Ranasinghe, D.; Zakrzewski, V. G.; Gao, J.; Rega, N.; Zheng, G.; Liang, W.; Hada, M.; Ehara, M.; Toyota, K.; Fukuda, R.; Hasegawa, J.; Ishida, M.; Nakajima, T.; Honda, Y.; Kitao, O.; Nakai, H.; Vreven, T.; Throssell, K.; Montgomery Jr., J. A.; Peralta, J. E.; Ogliaro, F.; Bearpark, M. J.; Heyd, J. J.; Brothers, E. N.; Kudin, K. N.; Staroverov, V. N.; Keith, T. A.; Kobayashi, R.; Normand, J.; Raghavachari, K.; Rendell, A. P.; Burant, J. C.; Iyengar, S. S.; Tomasi, J.; Cossi, M.; Millam, J. M.; Klene, M.; Adamo, C.; Cammi, R.; Ochterski, J. W.; Martin, R. L.; Morokuma, K.; Farkas, O.; Foresman, J. B.; Fox, D. J. *Gaussian 16*, Revision A.03; Gaussian, Inc.: Wallingford, CT, 2016.

Table S1: Principal coordinates for the structures of (*E*)- and (*Z*)-1,2,3,3,3-pentafluoropropene and their argon complexes

Theoretical Structures

	(*E*)-$CHFCFCF_3$			(*Z*)-$CHFCFCF_3$		
	a	*b*	*c*	*a*	*b*	*c*
C1	-1.7258	-0.4571	0.0000	-1.4758	-0.7061	0.0000
C2	-0.4139	-0.6800	0.0000	-0.5095	0.2067	0.0000
C3	0.7188	0.2972	0.0000	0.9466	-0.1245	0.0000
F1	-2.2586	0.7652	0.0000	-2.7605	-0.3486	0.0000
F2	0.0157	-1.9543	0.0000	-0.7593	1.5182	0.0000
F3	0.2834	1.5571	0.0000	1.1332	-1.4528	0.0000
F4	1.4931	0.1149	-1.0810	1.5554	0.3854	-1.0798
F5	1.4931	0.1149	1.0810	1.5554	0.3854	1.0798
H	-2.4385	-1.2655	0.0000	-1.2867	-1.7642	0.0000

Experimental Structures

	(*E*)-$CHFCFCF_3$			(*Z*)-$CHFCFCF_3$		
	a	*b*	*c*	*a*	*b*	*c*
C1	-1.7318	-0.4503	0.0000	-1.4738	-0.7125	0.0000
C2	-0.3981	-0.6798	0.0000	-0.4865	0.2015	0.0000
C3	0.7140	0.2930	0.0000	0.9385	-0.1221	0.0000
F1	-2.2479	0.7793	0.0000	-2.7739	-0.3366	0.0000
F2	0.0020	-1.9637	0.0000	-0.7540	1.5096	0.0000
F3	0.2822	1.5368	0.0000	1.1336	-1.4491	0.0000
F4	1.4939	0.1215	-1.0787	1.5542	0.3850	-1.0773
F5	1.4939	0.1215	1.0787	1.5542	0.3850	1.0773
H	-2.4463	-1.2571	0.0000	-1.2953	-1.7724	0.0000

Theoretical Structures

	Ar-(*E*)-$CHFCFCF_3$			Ar-(*Z*)-$CHFCFCF_3$		
	a	*b*	*c*	*a*	*b*	*c*
C1	-0.2007	-1.8047	-0.0993	-0.0809	1.5927	-0.5912
C2	-0.9004	-0.7020	-0.4542	-0.4705	0.6112	0.2425
C3	-1.0386	0.5832	0.2616	-1.4313	-0.4338	-0.1041
F1	0.5062	-1.8732	1.0293	0.8153	2.5279	-0.1989
F2	-1.5749	-0.7529	-1.6165	0.0124	0.5253	1.4843
F3	-0.3649	0.5935	1.3927	-1.8739	-0.2868	-1.3616
F4	-0.5968	1.5983	-0.4971	-0.8850	-1.6534	0.0006
F5	-2.3272	0.8343	0.5405	-2.4941	-0.4100	0.7124
H	-0.1814	-2.6875	-0.7171	-0.4485	1.6923	-1.5963
Ar	2.7187	0.4552	-0.2979	2.7105	-0.9084	-0.1264

Table S1: Principal coordinates for the structures of (*E*)- and (*Z*)-1,2,3,3,3-pentafluoropropene and their argon complexes

Experimental Structures

	Ar-(*E*)-$CHFCFCF_3$			Ar-(*Z*)-$CHFCFCF_3$		
	a	*b*	*c*	*a*	*b*	*c*
C1	-0.2160	-1.8037	-0.0871	-0.0706	1.5922	-0.5380
C2	-0.9201	-0.7049	-0.4457	-0.4595	0.5736	0.2503
C3	-1.0487	0.5885	0.2568	-1.4940	-0.3959	-0.1033
F1	0.5057	-1.8592	1.0328	0.8965	2.4522	-0.1421
F2	-1.6098	-0.7692	-1.5983	0.0940	0.3756	1.4491
F3	-0.3602	0.6119	1.3788	-2.0051	-0.1378	-1.3163
F4	-2.3335	0.8433	0.5497	-1.0078	-1.6449	-0.1183
F5	-0.6167	1.5946	-0.5195	-2.5016	-0.3790	0.7805
H	-0.2050	-2.6936	-0.6948	-0.4938	1.7813	-1.5077
Ar	2.7599	0.4441	-0.3005	2.7710	-0.8930	-0.1549

Table S2: Observed transition frequencies (in MHz) for (E)-$CHFCFCF_3$

J'	K_a'	K_c'	J''	K_a''	K_c''	Observed	Obs - Calc
11	5	6	11	5	7	2692.4012	0.0007
3	2	1	3	1	2	2753.8250	-0.0017
3	1	2	3	1	3	2810.0731	-0.0006
2	2	0	2	1	1	2853.8542	-0.0012
9	4	5	9	4	6	2988.8630	-0.0001
4	2	2	4	1	3	3067.7756	-0.0008
3	1	2	3	0	3	3096.9112	-0.0010
6	1	6	5	3	3	3101.9871	-0.0043
5	2	3	5	2	4	3119.1410	-0.0015
7	3	4	7	3	5	3153.5730	0.0001
9	5	5	8	7	2	3167.4147	0.0083
1	0	1	0	0	0	3181.5052	-0.0013
6	1	5	5	4	2	3547.5927	-0.0077
8	1	8	7	3	5	3579.4161	0.0141
5	2	3	5	1	4	3936.3859	-0.0006
6	2	5	5	4	2	3943.9830	0.0081
1	1	1	0	0	0	4085.2065	-0.0023
2	2	1	2	1	2	4130.3641	-0.0018
3	0	3	2	2	0	4195.8698	-0.0057
6	3	3	6	2	4	4210.4958	-0.0009
14	6	8	14	6	9	4232.8879	-0.0013
5	3	2	5	2	3	4243.3548	-0.0008
3	1	3	2	2	0	4482.7102	-0.0037
7	3	5	6	5	2	4485.5001	-0.0053
4	1	3	4	1	4	4543.2172	-0.0015
4	3	1	4	2	2	4662.0952	-0.0016
4	1	3	4	0	4	4662.9587	0.0002
12	5	7	12	5	8	4691.3611	-0.0003
2	2	1	2	0	2	4703.7222	-0.0029
7	3	4	7	2	5	4770.2699	-0.0011
5	2	3	4	4	0	4796.9904	-0.0030
3	2	2	3	1	3	4918.9988	-0.0016
6	2	4	6	2	5	4984.5466	-0.0014
10	4	6	10	4	7	5006.4029	-0.0014
8	3	5	8	3	6	5121.7923	-0.0008
3	3	0	3	2	1	5163.8081	-0.0016
3	2	2	3	0	3	5205.8359	-0.0030
2	0	2	1	1	1	5316.5544	-0.0025
4	1	3	3	3	0	5375.2420	-0.0003
6	2	4	6	1	5	5380.9221	-0.0003
8	4	4	8	3	5	5501.3101	-0.0023
7	1	6	6	4	3	5542.9061	0.0074
9	4	5	9	3	6	5690.7424	0.0014

Table S2: Observed transition frequencies (in MHz) for (E)-$CHFCFCF_3$

J'	K_a'	K_c'	J''	K_a''	K_c''	Observed	Obs - Calc
7	2	6	6	4	3	5712.1672	-0.0043
3	3	1	3	2	2	5780.2709	-0.0015
4	0	4	3	2	1	5876.0884	-0.0051
2	1	2	1	1	1	5889.9127	-0.0034
7	4	3	7	3	4	5894.7085	-0.0018
4	2	3	4	1	4	5971.6092	-0.0014
4	1	4	3	2	1	5995.8282	-0.0051
8	3	5	8	2	6	5998.9987	0.0004
4	2	3	4	0	4	6091.3496	-0.0007
4	3	2	4	2	3	6113.8622	-0.0056
2	0	2	1	0	1	6220.2537	-0.0054
5	1	4	5	1	5	6430.1331	-0.0022
5	1	4	5	0	5	6474.7529	-0.0047
6	4	2	6	3	3	6579.2500	-0.0021
10	4	6	10	3	7	6611.7720	-0.0006
5	0	5	4	2	2	6661.2240	-0.0067
6	0	6	5	2	3	6664.4484	-0.0083
6	1	6	5	2	3	6679.9782	0.0198
5	3	3	5	2	4	6697.1066	-0.0029
5	1	5	4	2	2	6705.8479	-0.0051
15	6	9	15	6	10	6731.1255	0.0141
11	5	6	11	4	7	6767.8078	-0.0019
2	1	2	1	0	1	6793.6130	-0.0053
4	2	3	3	3	0	6803.6310	-0.0031
2	1	1	1	1	0	6836.0852	-0.0030
8	1	7	7	4	4	6881.4228	-0.0157
8	2	7	7	4	4	6947.7067	-0.0073
10	5	5	10	4	6	6984.2170	-0.0065
7	2	5	7	2	6	7058.0778	-0.0047
8	3	6	7	5	3	7090.7164	-0.0008
13	5	8	13	5	9	7171.2876	-0.0039
5	4	1	5	3	2	7217.3101	-0.0034
7	2	5	7	1	6	7227.3537	-0.0016
5	2	4	5	1	5	7247.3755	-0.0038
5	2	4	5	0	5	7291.9996	-0.0021
12	5	7	12	4	8	7303.5093	-0.0050
9	3	6	9	3	7	7372.3361	-0.0042
11	4	7	11	4	8	7398.8687	-0.0046
3	1	2	2	2	1	7435.5319	-0.0049
5	3	3	5	1	4	7514.3518	-0.0017
4	3	2	4	1	3	7542.2566	-0.0030
6	3	4	6	2	5	7555.6974	-0.0036
4	4	0	4	3	1	7613.2830	-0.0017

Table S2: Observed transition frequencies (in MHz) for (E)-$CHFCFCF_3$

J'	K_a'	K_c'	J''	K_a''	K_c''	Observed	Obs - Calc
7	3	4	6	5	1	7631.7092	-0.0114
9	2	8	8	4	5	7670.7612	-0.0070
9	5	4	9	4	5	7712.2903	-0.0034
9	3	6	9	2	7	7789.5726	-0.0026
4	4	1	4	3	2	7796.2300	-0.0070
5	4	2	5	3	3	7842.3223	-0.0054
3	3	1	3	1	2	7889.1967	-0.0025
10	1	9	9	4	6	7942.4235	-0.0110
6	3	4	6	1	5	7952.0716	-0.0037
6	4	3	6	3	4	8030.4464	-0.0056
13	6	7	13	5	8	8050.5736	-0.0060
14	6	8	14	5	9	8145.8871	0.0007
11	4	7	11	3	8	8236.4891	-0.0007
6	1	5	6	1	6	8287.9312	-0.0056
6	1	5	6	0	6	8303.4328	-0.0057
5	3	3	4	4	0	8374.9557	-0.0047
7	4	4	7	3	5	8437.7851	-0.0008
3	0	3	2	1	2	8468.9869	-0.0036
4	2	2	3	3	1	8471.4519	-0.0039
5	1	4	4	3	1	8473.8883	-0.0033
8	5	3	8	4	4	8588.1108	-0.0096
13	5	8	13	4	9	8651.0742	0.0006
12	6	6	12	5	7	8672.1456	0.0011
7	3	5	7	2	6	8674.7785	-0.0020
6	2	5	6	1	6	8684.3070	-0.0042
6	2	5	6	0	6	8699.8083	-0.0046
3	1	3	2	1	2	8755.8238	-0.0052
7	3	5	7	1	6	8844.0456	-0.0077
6	2	4	5	4	1	8888.1397	-0.0088
3	0	3	2	0	2	9042.3467	-0.0031
5	3	2	4	4	1	9045.0047	-0.0055
8	4	5	8	3	6	9115.4293	-0.0090
15	6	9	15	5	10	9117.2176	-0.0054
8	2	6	8	2	7	9128.2092	-0.0034
8	2	6	8	1	7	9194.4808	-0.0073
16	7	9	16	6	10	9195.4714	-0.0104
5	2	4	4	3	1	9291.1277	-0.0079
7	5	2	7	4	3	9293.9667	-0.0046
3	1	3	2	0	2	9329.1924	0.0042
3	2	2	2	2	1	9544.4599	-0.0037
2	2	1	1	1	0	9547.1866	-0.0078
6	4	3	5	5	0	9556.1294	-0.0073
15	7	8	15	6	9	9564.6764	0.0004

Table S2: Observed transition frequencies (in MHz) for (E)-$CHFCFCF_3$

J'	K_a'	K_c'	J''	K_a''	K_c''	Observed	Obs - Calc
11	6	5	11	5	6	9672.9525	-0.0026
10	3	7	10	3	8	9678.6405	-0.0028
17	7	10	17	6	11	9712.6766	-0.0034
6	5	1	6	4	2	9720.6476	-0.0129
6	4	2	5	5	1	9744.9724	0.0030
10	3	7	10	2	8	9858.4448	-0.0088
7	5	3	7	4	4	9862.4036	-0.0111
6	5	2	6	4	3	9901.4342	-0.0124
14	5	9	14	5	10	9904.2239	-0.0084
8	5	4	8	4	5	9927.0425	0.0052
5	5	0	5	4	1	9933.6140	-0.0028
12	4	8	12	4	9	9934.7236	-0.0236
5	5	1	5	4	2	9973.2880	-0.0142
8	4	5	8	2	6	9992.6462	0.0027
8	3	6	8	2	7	10005.4150	-0.0028
3	2	1	2	2	0	10046.6073	-0.0069
7	1	6	7	1	7	10050.4420	-0.0096
7	4	4	7	2	5	10054.4728	-0.0111
7	1	6	7	0	7	10055.5805	-0.0106
8	3	6	8	1	7	10071.6807	-0.0127
9	4	6	9	3	7	10074.2109	-0.0072
3	1	2	2	1	1	10146.6424	-0.0006
2	2	0	1	1	1	10163.0360	0.0048
7	2	6	7	1	7	10219.7201	-0.0043
7	2	6	7	0	7	10224.8605	-0.0034
12	4	8	12	3	9	10327.2357	0.0067
9	4	6	9	2	7	10491.4496	-0.0035
14	7	7	14	6	8	10550.4806	-0.0099
6	4	3	6	2	4	10601.6097	0.0047
14	5	9	14	4	10	10653.0532	-0.0072
10	6	4	10	5	5	10658.4121	-0.0088
10	5	6	10	4	7	10681.6298	-0.0030
6	1	5	5	3	2	10724.5370	-0.0026
3	3	0	3	1	3	10727.7095	-0.0005
3	3	0	3	0	3	11014.5482	-0.0003
9	2	7	9	2	8	11065.5183	0.0051
2	2	0	1	0	1	11066.7323	-0.0011
9	2	7	9	1	8	11089.9486	-0.0056
6	2	5	5	3	2	11120.9173	0.0033
4	1	3	3	2	2	11183.9468	-0.0051
10	4	7	10	3	8	11284.0092	-0.0024
9	6	3	9	5	4	11380.4975	-0.0024
5	4	2	5	2	3	11420.2936	-0.0011

Table S2: Observed transition frequencies (in MHz) for (E)-$CHFCFCF_3$

J'	K_a'	K_c'	J''	K_a''	K_c''	Observed	Obs - Calc
4	0	4	3	1	3	11439.9900	-0.0039
6	3	4	5	4	1	11459.2991	-0.0024
10	4	7	10	2	8	11463.8214	-0.0006
11	5	7	11	4	8	11474.2862	0.0038
9	3	7	9	2	8	11482.7564	0.0083
9	3	7	9	1	8	11507.1854	-0.0037
4	1	4	3	1	3	11559.7281	-0.0055
4	0	4	3	0	3	11726.8327	0.0004
13	7	6	13	6	7	11738.7395	0.0001
8	1	7	8	1	8	11739.8204	-0.0020
8	1	7	8	0	8	11741.4695	-0.0009
8	2	7	8	1	8	11806.0925	-0.0053
8	2	7	8	0	8	11807.7410	-0.0048
8	6	2	8	5	3	11816.4425	-0.0008
10	6	5	10	5	6	11825.1215	0.0003
4	1	4	3	0	3	11846.5762	0.0042
11	3	8	11	3	9	11848.8759	-0.0026
9	6	4	9	5	5	11865.7738	0.0025
11	3	8	11	2	9	11921.1313	-0.0024
7	1	6	6	3	3	11934.0084	0.0014
11	6	6	11	5	7	11935.0450	0.0147
8	6	3	8	5	4	11977.0295	-0.0042
7	6	1	7	5	2	12057.6034	-0.0016
7	6	2	7	5	3	12098.4497	0.0018
7	2	6	6	3	3	12103.2716	-0.0083
8	1	7	7	3	4	12165.6518	0.0003
8	3	5	7	5	2	12170.4653	0.0089
6	6	0	6	5	1	12190.3422	0.0151
6	6	1	6	5	2	12197.5799	-0.0094
8	2	7	7	3	4	12231.9069	-0.0200
3	2	2	2	1	1	12255.5681	-0.0017
4	4	1	4	2	2	12270.7108	-0.0095
4	3	1	4	1	4	12273.0955	0.0035
12	6	7	12	5	8	12274.2124	0.0109
10	5	6	10	3	7	12287.0092	0.0080
11	5	7	11	3	8	12311.8978	-0.0011
13	4	9	13	4	10	12374.4151	-0.0019
4	3	1	4	0	4	12392.8389	0.0072
13	4	9	13	3	10	12544.2853	0.0013
12	5	8	12	4	9	12546.9058	0.0057
7	2	5	6	4	2	12582.1037	-0.0064
16	8	8	16	7	9	12583.8628	-0.0006
5	2	3	4	3	2	12597.8889	-0.0027

Table S2: Observed transition frequencies (in MHz) for (E)-$CHFCFCF_3$

J'	K_a'	K_c'	J''	K_a''	K_c''	Observed	Obs - Calc
4	2	3	3	2	2	12612.3371	-0.0066
11	4	8	11	3	9	12686.4915	-0.0034
11	4	8	11	2	9	12758.7476	-0.0025
12	7	5	12	6	6	12760.7639	-0.0119
10	2	8	10	2	9	12866.6973	-0.0070
10	2	8	10	1	9	12875.3365	0.0039
9	5	5	9	3	6	12879.1949	0.0077
13	6	8	13	5	9	12896.8212	-0.0060
7	4	4	6	5	1	12915.9271	-0.0065
12	5	8	12	3	9	12939.3736	-0.0083
4	3	2	3	3	1	12945.9357	-0.0034
10	3	8	10	2	9	13046.5187	0.0040
10	3	8	10	1	9	13055.1443	0.0012
4	3	1	3	3	0	13105.1171	0.0015
6	3	3	5	4	2	13139.0249	0.0054
4	1	3	3	1	2	13292.8704	-0.0082
9	1	8	9	1	9	13390.3664	-0.0024
9	1	8	9	0	9	13390.8769	-0.0072
9	2	8	9	1	9	13414.8118	0.0020
9	2	8	9	0	9	13415.3192	-0.0059
11	7	4	11	6	5	13466.9023	-0.0101
7	4	3	6	5	2	13533.7930	0.0045
4	2	2	3	2	1	13606.8252	-0.0033
13	7	7	13	6	8	13720.8893	-0.0017
12	7	6	12	6	7	13734.7188	0.0010
12	3	9	12	3	10	13823.0775	-0.0016
11	7	5	11	6	6	13864.2222	0.0015
10	7	3	10	6	4	13901.3860	-0.0001
4	4	0	4	2	3	13914.7702	0.0042
8	5	4	8	3	5	13920.6760	-0.0064
13	5	9	13	3	10	14024.0676	0.0015
8	5	4	7	6	1	14033.5361	0.0023
10	7	4	10	6	5	14035.8766	-0.0001
9	7	2	9	6	3	14163.0508	0.0076
7	3	5	6	4	2	14198.8074	-0.0008
9	7	3	9	6	4	14200.1825	0.0039
8	5	3	7	6	2	14203.5121	0.0170
12	4	9	12	3	10	14215.5639	0.0032
12	4	9	12	2	10	14243.1534	0.0069
5	0	5	4	1	4	14272.2370	0.0112
5	1	5	4	1	4	14316.8555	0.0074
3	2	1	2	1	2	14319.7395	0.0102
8	7	1	8	6	2	14325.5952	0.0030

Table S2: Observed transition frequencies (in MHz) for (*E*)-$CHFCFCF_3$

J'	K_a'	K_c'	J''	K_a''	K_c''	Observed	Obs - Calc
8	7	2	8	6	3	14333.5685	0.0023
5	0	5	4	0	4	14391.9755	0.0100
5	1	5	4	0	4	14436.5897	0.0018
14	6	9	14	4	10	14566.0581	0.0006
11	2	9	11	2	10	14581.0238	0.0173
11	2	9	11	1	10	14583.9626	0.0104
11	3	9	11	2	10	14653.2610	-0.0007
5	3	2	5	0	5	14654.5046	0.0048
11	3	9	11	1	10	14656.2051	-0.0024
4	2	3	3	1	2	14721.2709	0.0004
12	6	7	12	4	8	14886.3542	-0.0003
3	2	1	2	0	2	14893.0867	-0.0018
10	2	9	10	0	10	15031.7617	0.0195
7	5	3	7	3	4	15146.6241	-0.0036
3	3	1	2	2	0	15181.9853	-0.0015
14	5	10	14	4	11	15331.0033	-0.0204
4	4	0	4	1	3	15343.1581	0.0003
3	3	0	2	2	1	15353.1763	0.0032
15	6	10	15	4	11	15354.8010	0.0168
8	2	6	7	4	3	15465.4360	0.0066
5	2	4	4	2	3	15592.6184	0.0015
13	3	10	13	2	11	15655.4368	0.0102
14	8	7	14	7	8	15659.8576	0.0042
18	6	12	18	5	13	15679.9997	0.0031
6	4	2	6	2	5	15774.3006	0.0039
13	4	10	13	3	11	15815.1537	-0.0150
13	4	10	13	2	11	15825.3160	0.0224
13	8	6	13	7	7	15863.3075	-0.0019
12	8	4	12	7	5	15976.4271	-0.0010
12	8	5	12	7	6	16084.9214	0.0133
8	4	5	7	5	2	16164.0946	-0.0069
6	4	2	6	1	5	16170.6761	0.0050
5	3	3	4	3	2	16175.8643	0.0057
5	1	4	4	1	3	16203.7686	0.0039
5	4	2	4	4	1	16221.9578	0.0086
12	2	10	12	1	11	16250.8945	-0.0007
11	8	3	11	7	4	16254.1221	0.0003
5	4	1	4	4	0	16257.6647	0.0022
12	3	10	12	2	11	16277.5032	0.0016
12	3	10	12	1	11	16278.4943	0.0134
11	8	4	11	7	5	16285.7035	0.0125
6	5	2	6	3	3	16292.5699	0.0149
8	3	6	7	4	3	16342.6340	-0.0006

Table S2: Observed transition frequencies (in MHz) for (E)-$CHFCFCF_3$

J'	K_a'	K_c'	J''	K_a''	K_c''	Observed	Obs - Calc
10	8	2	10	7	3	16441.8645	0.0024
10	8	3	10	7	4	16449.4243	-0.0045
16	6	11	16	4	12	16561.7492	0.0017
9	8	1	9	7	2	16572.9127	0.0060
9	8	2	9	7	3	16574.3085	-0.0116
11	1	10	11	1	11	16647.3959	-0.0084
11	2	10	11	0	11	16650.4116	0.0138
5	3	2	4	3	1	16653.6429	0.0093
6	2	4	5	3	3	16770.8605	0.0099
15	5	11	15	4	12	16912.5650	-0.0016
16	9	7	16	8	8	16981.3399	-0.0001
5	2	4	4	1	3	17021.0209	0.0121
6	0	6	5	1	5	17030.9851	0.0067
6	1	6	5	1	5	17046.4885	0.0084
5	2	3	4	2	2	17072.3762	0.0014
6	0	6	5	0	5	17075.6068	0.0059
6	1	6	5	0	5	17091.1132	0.0107
5	5	1	5	3	2	17150.2395	-0.0018
9	2	7	8	4	4	17228.6050	-0.0090
14	4	11	14	3	12	17447.2770	-0.0109
14	4	11	14	2	12	17450.8941	0.0053
9	5	5	8	6	2	17492.8081	-0.0046
10	6	5	10	4	6	17500.3510	0.0014
7	3	4	6	4	3	17540.5313	0.0064
15	9	6	15	8	7	17619.7253	0.0073
9	3	7	8	4	4	17645.8532	0.0043
8	4	4	7	5	3	17713.8269	0.0042
7	4	3	7	2	6	17723.0672	0.0035
5	5	0	5	3	3	17816.3230	0.0041
10	2	8	9	4	5	17828.9080	0.0040
15	9	7	15	8	8	17865.3913	-0.0017
6	3	3	6	1	6	17879.3619	0.0060
7	4	3	7	1	6	17892.3540	0.0176
13	3	11	13	2	12	17906.7122	-0.0141
13	3	11	13	1	12	17907.0370	-0.0079
6	5	1	6	3	4	17939.2581	0.0018
9	5	4	8	6	3	18024.8335	0.0134
14	9	5	14	8	6	18042.7150	0.0024
3	3	0	2	1	1	18064.2907	0.0114
4	3	2	3	2	1	18081.3190	0.0073
6	1	5	5	2	4	18087.0368	-0.0008

Table S3: Observed transition frequencies (in MHz) for (E)-^{13}CHFCFCF$_3$

J'	K_a'	K_c'	J''	K_a''	K_c''	Observed	Obs - Calc
3	1	2	3	1	3	2763.4062	-0.0042
3	2	1	3	1	2	2786.1031	0.0002
2	2	0	2	1	1	2898.0582	-0.0010
7	3	4	7	3	5	3014.1923	0.0020
5	2	3	5	2	4	3028.8484	-0.0028
3	1	2	3	0	3	3065.5256	0.0033
4	2	2	4	1	3	3072.3544	-0.0044
1	0	1	0	0	0	3150.4945	0.0007
5	2	3	5	1	4	3897.5934	0.0027
1	1	1	0	0	0	4070.9845	0.0025
2	2	1	2	1	2	4156.3835	0.0010
6	3	3	6	2	4	4246.1737	-0.0020
5	3	2	5	2	3	4319.1800	-0.0005
4	1	3	4	1	4	4474.8030	0.0053
4	1	3	4	0	4	4603.6771	0.0011
7	3	4	7	2	5	4742.6458	0.0117
4	3	1	4	2	2	4751.7053	-0.0003
6	2	4	6	2	5	4862.0025	0.0061
3	2	2	3	0	3	5231.7384	-0.0027
2	0	2	1	1	1	5243.8934	-0.0070
8	4	4	8	3	5	5592.8539	-0.0084
9	4	5	9	3	6	5700.2266	0.0086
2	1	2	1	1	1	5836.0063	0.0010
3	3	1	3	2	2	5840.6984	-0.0009
8	3	5	8	2	6	5895.9185	-0.0002
4	2	3	4	1	4	5962.8614	-0.0045
7	4	3	7	3	4	6032.1089	0.0023
4	2	3	4	0	4	6091.7429	-0.0014
4	3	2	4	2	3	6161.0984	-0.0022
2	0	2	1	0	1	6164.3899	0.0013
5	1	4	5	1	5	6349.4137	-0.0011
5	1	4	5	0	5	6398.4913	0.0014
6	4	2	6	3	3	6723.2355	0.0019
5	3	3	5	2	4	6723.5533	0.0063
2	1	2	1	0	1	6756.4955	0.0019
2	1	1	1	1	0	6765.9492	-0.0006
7	2	5	7	2	6	6917.0994	-0.0152
7	2	5	7	1	6	7105.6592	0.0091
5	2	4	5	1	5	7218.1556	0.0012
5	2	4	5	0	5	7267.2542	0.0247
6	3	4	6	2	5	7555.4430	-0.0026
5	3	3	5	1	4	7592.2691	-0.0175
4	4	0	4	3	1	7721.0056	-0.0066

Table S3: Observed transition frequencies (in MHz) for (E)-^{13}CHFCFCF$_3$

J'	K_a'	K_c'	J''	K_a''	K_c''	Observed	Obs - Calc
4	4	1	4	3	2	7891.7083	0.0058
9	5	4	9	4	5	7922.5082	0.0042
5	4	2	5	3	3	7931.7103	-0.0009
3	3	1	3	1	2	8006.9279	0.0098
6	4	3	6	3	4	8105.1028	0.0014
6	1	5	6	1	6	8205.0591	0.0008
6	1	5	6	0	6	8222.4744	0.0011
3	0	3	2	1	2	8376.0726	0.0018
7	4	4	7	3	5	8487.5710	0.0017
6	2	5	6	1	6	8636.3498	-0.0096
6	2	5	6	0	6	8653.7634	-0.0109
3	1	3	2	1	2	8678.1812	-0.0015
7	3	5	7	1	6	8834.0908	-0.0031
3	0	3	2	0	2	8968.1778	0.0022
8	4	5	8	3	6	9131.6762	-0.0123
3	1	3	2	0	2	9270.2855	-0.0022
3	2	2	2	2	1	9451.4287	-0.0006
7	5	2	7	4	3	9463.0659	-0.0032
2	2	1	1	1	0	9527.4142	0.0002
6	5	1	6	4	2	9864.2703	0.0032
8	3	6	8	2	7	9949.0912	0.0020
7	1	6	7	1	7	9967.6753	0.0006
7	1	6	7	0	7	9973.5750	0.0035
7	5	3	7	4	4	9984.7792	0.0025
6	5	2	6	4	3	10028.6652	-0.0024
2	2	0	1	1	1	10128.9812	-0.0014
4	1	3	3	2	2	11005.2563	-0.0017
4	0	4	3	1	3	11331.2081	-0.0029
4	1	4	3	1	3	11460.0879	-0.0015
4	0	4	3	0	3	11633.3233	0.0003
3	2	2	2	1	1	12212.8872	-0.0064
4	2	3	3	2	2	12493.3259	-0.0003
4	3	2	3	3	1	12813.7264	-0.0011
4	3	1	3	3	0	12962.2026	-0.0031
4	1	3	3	1	2	13171.4733	-0.0034
4	2	2	3	2	1	13457.7302	-0.0023
5	0	5	4	1	4	14146.6495	0.0020
5	1	5	4	1	4	14195.7258	0.0032
5	0	5	4	0	4	14275.5244	-0.0014
5	1	5	4	0	4	14324.6093	0.0083
4	2	3	3	1	2	14659.5384	-0.0066
3	3	1	2	2	0	15155.5419	0.0080
3	3	0	2	2	1	15318.5325	-0.0135

Table S3: Observed transition frequencies (in MHz) for (E)-$^{13}CHFCFCF_3$

J'	K_a'	K_c'	J''	K_a''	K_c''	Observed	Obs - Calc
5	2	4	4	2	3	15451.0228	0.0117
5	3	3	4	3	2	16013.4587	0.0013
5	4	2	4	4	1	16053.4756	0.0096
5	1	4	4	1	3	16070.3431	0.0034
5	4	1	4	4	0	16085.7554	-0.0116
5	3	2	4	3	1	16463.0509	0.0044
6	0	6	5	1	5	16886.1519	-0.0139
5	2	3	4	2	2	16895.5724	0.0007
6	1	6	5	1	5	16903.5738	-0.0069
6	0	6	5	0	5	16935.2438	0.0029
5	2	4	4	1	3	16939.0898	0.0105
6	1	6	5	0	5	16952.6586	0.0028
6	1	5	5	2	4	17890.4815	-0.0032
4	3	2	3	2	1	18034.5510	0.0083

Table S4: Observed transition frequencies (in MHz) for (E)-CHF^{13}CFCF$_3$

J'	K_a'	K_c'	J''	K_a''	K_c''	Observed	Obs - Calc
3	2	1	3	1	2	2741.3544	0.0003
3	1	2	3	1	3	2816.6706	0.0240
2	2	0	2	1	1	2838.2114	0.0025
4	2	2	4	1	3	3061.6455	-0.0025
3	1	2	3	0	3	3099.3262	-0.0093
5	2	3	5	2	4	3135.9195	-0.0069
1	0	1	0	0	0	3178.2429	-0.0005
7	3	4	7	3	5	3182.5376	0.0070
5	2	3	5	1	4	3939.4636	0.0009
1	1	1	0	0	0	4076.3109	0.0105
2	2	1	2	1	2	4116.9522	0.0025
6	3	3	6	2	4	4195.2200	-0.0042
4	1	3	4	1	4	4552.0489	-0.0001
4	3	1	4	2	2	4632.3067	-0.0049
2	2	1	2	0	2	4684.7891	0.0095
7	3	4	7	2	5	4769.7439	0.0038
3	2	2	3	1	3	4908.0034	-0.0053
6	2	4	6	2	5	5005.8605	0.0084
3	3	0	3	2	1	5134.5663	0.0123
2	0	2	1	1	1	5314.3920	0.0045
6	2	4	6	1	5	5393.3461	0.0076
8	4	4	8	3	5	5470.7519	0.0154
9	4	5	9	3	6	5680.3989	0.0038
3	3	1	3	2	2	5755.6415	-0.0025
7	4	3	7	3	4	5851.6147	0.0021
2	1	2	1	1	1	5882.2173	-0.0001
4	2	3	4	1	4	5963.5475	-0.0040
4	2	3	4	0	4	6080.9315	0.0016
4	3	2	4	2	3	6091.9523	0.0069
2	0	2	1	0	1	6212.4422	-0.0022
5	1	4	5	1	5	6438.5711	-0.0017
5	1	4	5	0	5	6482.0812	-0.0021
6	4	2	6	3	3	6532.8550	0.0047
5	3	3	5	2	4	6679.3224	-0.0055
2	1	2	1	0	1	6780.2734	-0.0010
2	1	1	1	1	0	6830.7377	0.0021
7	2	5	7	2	6	7080.0076	-0.0002
5	4	1	5	3	2	7173.9397	0.0132
5	2	4	5	1	5	7242.1024	-0.0066
7	2	5	7	1	6	7244.5356	-0.0034
5	2	4	5	0	5	7285.6179	-0.0018
3	1	2	2	2	1	7443.3078	-0.0074
5	3	3	5	1	4	7482.8618	-0.0025

Table S4: Observed transition frequencies (in MHz) for (*E*)-$CHF^{13}CFCF_3$

J'	K_a'	K_c'	J''	K_a''	K_c''	Observed	Obs - Calc
4	3	2	4	1	3	7503.4407	-0.0072
6	3	4	6	2	5	7542.9884	-0.0147
4	4	0	4	3	1	7573.8346	-0.0005
4	4	1	4	3	2	7759.5759	0.0003
5	4	2	5	3	3	7807.1453	0.0035
6	3	4	6	1	5	7930.4864	-0.0032
6	4	3	6	3	4	7998.6511	-0.0061
6	1	5	6	1	6	8293.6052	-0.0002
6	1	5	6	0	6	8308.6381	-0.0039
7	4	4	7	3	5	8411.4987	0.0017
3	0	3	2	1	2	8460.9306	0.0013
6	2	5	6	1	6	8681.0907	-0.0011
6	2	5	6	0	6	8696.1245	-0.0039
3	1	3	2	1	2	8743.6122	-0.0060
7	3	5	7	1	6	8831.7416	-0.0070
8	4	5	8	3	6	9096.2819	0.0089
3	1	3	2	0	2	9311.4438	-0.0043
2	2	1	1	1	0	9524.9057	-0.0008
3	2	2	2	2	1	9534.6752	-0.0020
7	5	3	7	4	4	9815.7573	-0.0020
8	5	4	8	4	5	9884.0238	0.0007
5	5	0	5	4	1	9884.6383	-0.0061
3	2	1	2	2	0	10040.6263	-0.0049
7	1	6	7	1	7	10053.0656	-0.0019
7	1	6	7	0	7	10058.0346	0.0077
9	4	6	9	3	7	10062.6959	-0.0181
8	3	6	8	1	7	10066.1783	0.0084
3	1	2	2	1	1	10137.4824	-0.0036
2	2	0	1	1	1	10143.2129	0.0077
4	0	4	3	1	3	11425.3163	-0.0013
4	1	4	3	1	3	11542.6906	-0.0054
4	0	4	3	0	3	11708.0088	0.0024
4	1	4	3	0	3	11825.3829	-0.0019
3	2	2	2	1	1	12228.8467	-0.0013
4	2	3	3	2	2	12598.2319	-0.0069
4	3	2	3	3	1	12934.5395	-0.0007
4	3	1	3	3	0	13096.1436	-0.0064
4	1	3	3	1	2	13278.0943	-0.0040
4	2	2	3	2	1	13598.3900	-0.0023
5	0	5	4	1	4	14251.4431	0.0037
5	1	5	4	1	4	14294.9473	-0.0028
5	0	5	4	0	4	14368.8127	-0.0052
5	1	5	4	0	4	14412.3315	0.0030

Table S4: Observed transition frequencies (in MHz) for (*E*)-$CHF^{13}CFCF_3$

J'	K_a'	K_c'	J''	K_a''	K_c''	Observed	Obs - Calc
4	2	3	3	1	2	14689.6076	0.0068
5	1	4	4	2	3	14769.9944	0.0231
3	3	1	2	2	0	15146.2798	-0.0033
3	3	0	2	2	1	15319.2271	0.0039
5	2	4	4	2	3	15573.5201	0.0125
5	3	3	4	3	2	16160.8907	0.0006
5	1	4	4	1	3	16181.4792	0.0054
5	4	2	4	4	1	16208.4608	0.0045
5	4	1	4	4	0	16244.9908	0.0009
5	3	2	4	3	1	16644.9053	0.0068
5	2	4	4	1	3	16985.0083	-0.0018
6	0	6	5	1	5	17004.8100	-0.0051
6	1	6	5	1	5	17019.8531	0.0014
6	0	6	5	0	5	17048.3185	-0.0073
5	2	3	4	2	2	17059.2897	0.0013
6	1	6	5	0	5	17063.3521	-0.0102
4	3	2	3	2	1	18040.1914	-0.0008

Table S5: Observed transition frequencies (in MHz) for (E)-$CHFCF^{13}CF_3$

J'	K_a'	K_c'	J''	K_a''	K_c''	Observed	Obs - Calc
3	2	1	3	1	2	2758.0515	0.0014
3	1	2	3	1	3	2803.1397	-0.0005
2	2	0	2	1	1	2859.7964	0.0005
4	2	2	4	1	3	3067.9869	0.0005
3	1	2	3	0	3	3092.0939	0.0079
5	2	3	5	2	4	3105.9406	-0.0032
7	3	4	7	3	5	3133.2829	-0.0121
1	0	1	0	0	0	3176.0627	-0.0009
1	1	1	0	0	0	4082.0430	-0.0035
2	2	1	2	1	2	4133.5935	0.0011
6	3	3	6	2	4	4214.8364	0.0039
5	3	2	5	2	3	4253.5093	0.0021
4	1	3	4	1	4	4533.0388	0.0011
4	1	3	4	0	4	4654.0338	0.0026
4	3	1	4	2	2	4674.2625	-0.0009
2	2	1	2	0	2	4709.5332	-0.0027
7	3	4	7	2	5	4765.4552	-0.0018
6	2	4	6	2	5	4966.6296	0.0061
3	3	0	3	2	1	5175.0884	-0.0003
2	0	2	1	1	1	5304.2877	-0.0049
8	4	4	8	3	5	5513.2292	0.0053
3	3	1	3	2	2	5788.1947	0.0034
2	1	2	1	1	1	5880.2364	0.0001
4	2	3	4	1	4	5969.6879	-0.0020
8	3	5	8	2	6	5983.3521	0.0035
4	2	3	4	0	4	6090.6863	0.0030
4	3	2	4	2	3	6119.8588	0.0029
2	0	2	1	0	1	6210.2744	-0.0011
5	1	4	5	1	5	6418.0558	-0.0074
5	1	4	5	0	5	6463.2894	-0.0025
6	4	2	6	3	3	6598.9420	-0.0095
2	1	2	1	0	1	6786.2168	-0.0023
2	1	1	1	1	0	6823.9949	-0.0031
7	2	5	7	2	6	7037.4471	0.0083
7	2	5	7	1	6	7209.3249	-0.0019
5	4	1	5	3	2	7234.5098	-0.0049
5	2	4	5	1	5	7242.4218	0.0012
5	2	4	5	0	5	7287.6457	-0.0035
5	3	3	5	1	4	7524.4120	-0.0025
6	3	4	6	2	5	7554.7524	0.0027
4	4	0	4	3	1	7627.7649	0.0014
5	4	2	5	3	3	7854.1052	-0.0045
6	3	4	6	1	5	7955.8937	-0.0174

Table S5: Observed transition frequencies (in MHz) for (E)-CHFCF^{13}CF$_3$

J'	K_a'	K_c'	J''	K_a''	K_c''	Observed	Obs - Calc
6	4	3	6	3	4	8040.0849	0.0067
6	1	5	6	1	6	8275.4080	0.0107
6	1	5	6	0	6	8291.1591	0.0017
7	4	4	7	3	5	8443.7840	-0.0021
3	0	3	2	1	2	8452.8301	-0.0062
8	5	3	8	4	4	8615.6935	-0.0005
7	3	5	7	2	6	8669.6023	0.0015
6	2	5	6	1	6	8676.5463	-0.0124
6	2	5	6	0	6	8692.3321	0.0133
3	1	3	2	1	2	8741.7826	0.0004
7	3	5	7	1	6	8841.4873	-0.0015
8	2	6	8	2	7	9107.5126	-0.0078
8	2	6	8	1	7	9175.0346	-0.0012
5	2	4	4	3	1	9261.7902	0.0028
7	5	2	7	4	3	9317.0693	-0.0120
3	1	3	2	0	2	9317.7177	-0.0081
3	2	2	2	2	1	9528.1366	-0.0015
2	2	1	1	1	0	9541.9471	0.0008
6	5	1	6	4	2	9740.0537	0.0158
7	5	3	7	4	4	9878.6420	0.0024
6	5	2	6	4	3	9918.4112	0.0162
5	5	0	5	4	1	9951.0499	-0.0125
5	5	1	5	4	2	9990.1783	-0.0069
3	2	1	2	2	0	10027.5300	-0.0024
7	1	6	7	1	7	10037.7287	-0.0063
7	1	6	7	0	7	10042.9825	0.0067
8	3	6	8	1	7	10063.7912	-0.0007
9	4	6	9	3	7	10069.8324	0.0052
3	1	2	2	1	1	10129.2792	0.0009
2	2	0	1	1	1	10155.6826	0.0065
7	2	6	7	0	7	10214.8559	-0.0079
4	0	4	3	1	3	11420.5867	0.0055
4	1	4	3	1	3	11541.5739	-0.0007
4	0	4	3	0	3	11709.5240	-0.0031
4	1	4	3	0	3	11830.5184	-0.0020
3	2	2	2	1	1	12246.0889	0.0024
4	2	3	3	2	2	12591.3121	-0.0040
4	3	2	3	3	1	12922.9695	-0.0112
4	3	1	3	3	0	13080.5802	-0.0032
4	1	3	3	1	2	13271.4714	-0.0007
5	0	5	4	1	4	14249.4256	-0.0003
5	1	5	4	1	4	14294.6602	0.0057
5	0	5	4	0	4	14370.4107	-0.0086

Table S5: Observed transition frequencies (in MHz) for (E)-$CHFCF^{13}CF_3$

J'	K_a'	K_c'	J''	K_a''	K_c''	Observed	Obs - Calc
5	1	5	4	0	4	14415.6578	0.0100
5	1	4	4	2	3	14743.0323	0.0044
3	3	1	2	2	0	15174.4843	0.0025
3	3	0	2	2	1	15344.4738	0.0051
5	2	4	4	2	3	15567.3930	0.0078
5	3	3	4	3	2	16147.5961	0.0096
5	1	4	4	1	3	16179.6809	0.0009
5	4	2	4	4	1	16192.7858	0.0019
5	4	1	4	4	0	16227.9846	-0.0051
5	3	2	4	3	1	16621.2367	-0.0018
5	2	4	4	1	3	17004.0531	0.0158
6	0	6	5	1	5	17004.4505	-0.0029
6	1	6	5	1	5	17020.2093	-0.0041
5	2	3	4	2	2	17041.9975	0.0028
6	0	6	5	0	5	17049.6718	-0.0102
6	1	6	5	0	5	17065.4351	-0.0070
6	1	5	5	2	4	18053.1947	0.0045

Table S6: Observed transition frequencies (in MHz) for (Z)-$CHFCFCF_3$

J'	K_a'	K_c'	J''	K_a''	K_c''	Observed	Obs - Calc
8	2	6	8	2	7	2193.6114	-0.0023
5	2	4	4	3	1	2201.0649	0.0034
1	1	0	1	0	1	2268.4213	0.0023
2	1	1	2	0	2	2474.1231	0.0036
10	5	6	9	6	3	2563.2026	0.0008
10	5	5	9	6	4	2564.9218	0.0067
1	0	1	0	0	0	2627.0225	0.0027
5	2	3	4	3	2	2634.4091	0.0020
7	6	2	8	5	3	2773.0421	0.0058
7	6	1	8	5	4	2773.2066	0.0060
3	1	2	3	0	3	2806.0658	0.0037
5	1	4	5	1	5	2878.2632	0.0027
4	1	4	3	2	1	2932.1019	0.0051
2	0	2	1	1	1	3165.6712	0.0037
9	2	7	9	2	8	3182.1342	-0.0011
4	1	3	4	0	4	3289.4752	0.0046
7	3	5	6	4	2	3305.4417	-0.0050
7	3	4	6	4	3	3415.2611	0.0043
12	6	7	11	7	4	3513.3929	0.0082
12	6	6	11	7	5	3513.6854	-0.0097
10	8	3	11	7	4	3543.3709	0.0072
10	8	2	11	7	5		
17	9	9	16	10	6	3685.3880	0.0132
17	9	8	16	10	7		
5	5	1	6	4	2	3715.0848	0.0037
5	5	0	6	4	3	3715.9121	0.0024
5	1	4	5	0	5	3950.7461	0.0049
6	1	5	6	1	6	4006.1528	0.0003
14	3	11	14	3	12	4177.3849	-0.0149
9	4	6	8	5	3	4292.4158	0.0053
9	4	5	8	5	4	4315.7627	0.0052
10	2	8	10	2	9	4366.4911	0.0092
8	7	1	9	6	4	4486.3649	0.0039
8	7	2	9	6	3		
1	1	1	0	0	0	4702.5700	0.0069
6	2	5	5	3	2	4720.6555	0.0043
6	1	5	6	0	6	4808.7990	0.0070
5	1	5	4	2	2	4911.1547	0.0055
4	1	3	3	2	2	4920.8858	0.0064
2	1	2	1	1	1	5061.1661	0.0062
2	0	2	1	0	1	5241.2200	0.0092
11	5	7	10	6	4	5249.2765	0.0038
11	5	6	10	6	5	5253.8278	0.0127

Table S6: Observed transition frequencies (in MHz) for (*Z*)-$CHFCFCF_3$

J'	K_a'	K_c'	J''	K_a''	K_c''	Observed	Obs - Calc
11	9	3	12	8	4	5256.3481	0.0089
11	9	2	12	8	5		
7	1	6	7	1	7	5291.4592	0.0099
16	8	9	15	9	6	5414.0329	0.0118
16	8	8	15	9	7		
6	6	0	7	5	3	5427.0398	0.0022
6	6	1	7	5	2		
6	2	4	6	1	5	5436.5606	0.0071
2	1	1	1	1	0	5446.9200	0.0089
7	2	5	7	1	6	5447.6534	0.0100
5	2	3	5	1	4	5558.0077	0.0085
6	2	4	5	3	3	5561.9040	0.0071
8	2	6	8	1	7	5629.0606	0.0094
11	2	9	11	2	10	5730.5480	0.0123
4	2	2	4	1	3	5765.9181	0.0072
7	1	6	7	0	7	5866.2719	0.0079
3	0	3	2	1	2	5934.6335	0.0089
8	3	6	7	4	3	5961.5533	0.0071
3	2	1	3	1	2	6009.3639	-0.0011
9	2	7	9	1	8	6010.6452	-0.0021
9	8	1	10	7	4	6197.9703	0.0115
9	8	2	10	7	3		
8	3	5	7	4	4	6199.2586	-0.0012
2	2	0	2	1	1	6239.4509	0.0004
10	2	8	10	1	9	6615.0211	-0.0007
6	1	6	5	2	3	6658.5328	-0.0004
2	2	1	2	1	2	6805.2501	0.0001
10	4	7	9	5	4	6986.1738	0.0016
10	4	6	9	5	5	7040.0985	0.0011
3	2	2	3	1	3	7102.3185	-0.0002
8	1	7	8	0	8	7105.2041	-0.0016
2	1	2	1	0	1	7136.7043	0.0012
7	7	0	8	6	3	7137.3169	0.0009
7	7	1	8	6	2		
7	2	6	6	3	3	7162.7899	-0.0011
11	2	9	11	1	10	7455.0400	-0.0032
4	2	3	4	1	4	7502.1872	-0.0026
3	1	3	2	1	2	7583.9656	-0.0030
3	0	3	2	0	2	7830.1153	-0.0017
3	2	2	2	2	1	7881.0384	0.0011
3	2	1	2	2	0	7931.9699	-0.0042
12	5	8	11	6	5	7949.4366	-0.0047
12	5	7	11	6	6	7960.3885	0.0029

Table S6: Observed transition frequencies (in MHz) for (Z)-$CHFCFCF_3$

J'	K_a'	K_c'	J''	K_a''	K_c''	Observed	Obs - Calc
5	1	4	4	2	3	7978.1492	0.0002
5	2	4	5	1	5	8006.6457	-0.0020
17	8	10	16	9	7	8101.0711	0.0004
17	8	9	16	9	8		
7	1	7	6	2	4	8133.6028	-0.0002
3	1	2	2	1	1	8162.0601	0.0004
9	1	8	9	1	9	8223.8009	-0.0069
12	3	9	12	2	10	8246.7456	0.0019
13	3	10	13	2	11	8286.7080	0.0010
11	3	8	11	2	9	8404.4645	-0.0003
9	1	8	9	0	9	8489.3479	-0.0022
12	2	10	12	1	11	8528.7522	-0.0032
14	3	11	14	2	12	8560.3622	-0.0027
9	3	7	8	4	4	8604.0517	-0.0024
7	2	5	6	3	4	8616.1083	-0.0006
6	2	5	6	1	6	8616.3207	-0.0009
10	3	7	10	2	8	8712.4690	-0.0032
4	0	4	3	1	3	8733.7232	-0.0042
14	6	9	13	7	6	8899.8003	0.0015
14	6	8	13	7	7	8901.8904	0.0104
19	9	11	18	10	8	9051.7980	-0.0019
19	9	10	18	10	9		
9	3	6	8	4	5	9067.9499	-0.0009
15	3	12	15	2	13	9092.4486	-0.0026
9	3	6	9	2	7	9113.8213	0.0010
8	1	8	7	2	5	9308.2666	-0.0011
7	2	6	7	1	7	9329.9794	0.0011
3	1	3	2	0	2	9479.4630	0.0019
8	2	7	7	3	4	9494.6632	-0.0010
8	3	5	8	2	6	9547.6456	-0.0022
11	4	8	10	5	5	9690.5072	-0.0062
10	1	9	10	1	10	9797.8569	-0.0024
11	4	7	10	5	6	9804.4211	0.0011
13	2	11	13	1	12	9815.2642	-0.0026
16	3	13	16	2	14	9895.4160	-0.0014
7	3	4	7	2	5	9957.6217	-0.0041
10	1	9	10	0	10	9971.4312	-0.0051
4	1	4	3	1	3	10098.1801	0.0003
8	2	7	8	1	8	10144.0203	-0.0020
9	1	9	8	2	6	10171.5890	0.0035
6	3	3	6	2	4	10300.7907	0.0003
4	0	4	3	0	3	10383.0734	0.0019
4	2	3	3	2	2	10498.0456	-0.0053

Table S6: Observed transition frequencies (in MHz) for (Z)-$CHFCFCF_3$

J'	K_a'	K_c'	J''	K_a''	K_c''	Observed	Obs - Calc
4	3	2	3	3	1	10532.1451	-0.0007
4	3	1	3	3	0	10535.3444	-0.0002
5	3	2	5	2	3	10554.2040	0.0004
4	2	2	3	2	1	10623.0343	0.0083
13	5	9	12	6	6	10664.8932	0.0025
13	5	8	12	6	7	10689.2540	-0.0052
4	3	1	4	2	2	10716.7344	-0.0010
10	1	10	9	2	7	10729.0001	0.0001
3	3	0	3	2	1	10804.4157	-0.0010
4	1	3	3	1	2	10866.4723	-0.0077
3	3	1	3	2	2	10867.6423	-0.0040
18	4	14	18	3	15	10874.9604	-0.0113
4	3	2	4	2	3	10901.7398	-0.0014
17	3	14	17	2	15	10966.1738	-0.0109
5	3	3	5	2	4	10968.9368	-0.0177
17	4	13	17	3	14	10981.0436	-0.0049
11	1	11	10	2	8	10998.7885	-0.0036
12	1	12	11	2	9	11008.8324	-0.0061
19	4	15	19	3	16	11028.3509	0.0007
9	2	8	9	1	9	11052.3224	0.0025
6	3	4	6	2	5	11082.9708	-0.0006
6	1	5	5	2	4	11094.2929	-0.0050
10	3	8	9	4	5	11216.7927	-0.0011
7	3	5	7	2	6	11257.7584	0.0003
14	2	12	14	1	13	11275.1819	-0.0013
16	4	12	16	3	13	11303.4647	-0.0129
20	4	16	20	3	17	11470.3487	0.0012
11	1	10	11	0	11	11502.8036	0.0035
8	3	6	8	2	7	11506.5735	0.0032
5	0	5	4	1	4	11529.6002	0.0024
15	6	10	14	7	7	11613.6049	0.0049
15	6	9	14	7	8	11618.4094	-0.0050
2	2	1	1	1	0	11673.5395	0.0054
9	2	8	8	3	5	11676.2665	0.0089
4	1	4	3	0	3	11747.5265	0.0026
15	4	11	15	3	12	11786.1133	0.0034
8	2	6	7	3	5	11797.2579	-0.0021
9	3	7	9	2	8	11841.0633	-0.0003
2	2	0	1	1	1	11879.2386	0.0012
10	2	9	10	1	10	12046.3943	-0.0048
10	3	7	9	4	6	12048.3641	-0.0072
21	4	17	21	3	18	12215.5621	-0.0113
10	3	8	10	2	9	12270.5585	-0.0009

Table S6: Observed transition frequencies (in MHz) for (*Z*)-$CHFCFCF_3$

J'	K_a'	K_c'	J''	K_a''	K_c''	Observed	Obs - Calc
18	3	15	18	2	16	12282.7091	0.0054
14	4	10	14	3	11	12363.4602	0.0003
12	4	9	11	5	6	12401.0057	0.0082
5	1	5	4	1	4	12602.0780	-0.0003
12	4	8	11	5	7	12624.3336	0.0018
11	3	9	11	2	10	12801.4898	0.0004
15	2	13	15	1	14	12856.3097	-0.0013
5	0	5	4	0	4	12894.0513	0.0013
13	4	9	13	3	10	12967.9870	-0.0020
12	1	11	12	0	12	13042.4716	-0.0069
5	2	4	4	2	3	13106.5400	0.0038
11	2	10	11	1	11	13115.9806	-0.0033
5	4	2	4	4	1	13163.4695	0.0021
5	4	1	4	4	0	13163.6122	-0.0027
5	3	3	4	3	2	13173.7514	0.0019
5	3	2	4	3	1	13184.8735	-0.0037
22	4	18	22	3	19	13261.9299	-0.0046
5	2	3	4	2	2	13347.4092	0.0002
14	5	10	13	6	7	13396.0205	-0.0055
23	5	18	23	4	19	13436.0329	-0.0033
12	3	10	12	2	11	13437.0361	0.0046
14	5	9	13	6	8	13446.7743	0.0007
19	8	12	18	9	9	13505.1841	-0.0076
12	4	8	12	3	9	13539.4007	0.0014
5	1	4	4	1	3	13555.3225	0.0019
10	2	9	9	3	6	13661.5756	-0.0031
22	5	17	22	4	18	13694.0025	0.0128
11	3	9	10	4	6	13777.6555	0.0104
19	3	16	19	2	17	13803.6446	0.0039
5	1	5	4	0	4	13966.5354	0.0048
11	4	7	11	3	8	14033.2670	0.0100
3	2	2	2	1	1	14107.6593	-0.0010
13	3	11	13	2	12	14176.9554	0.0041
12	2	11	12	1	12	14249.7717	0.0168
7	1	6	6	2	5	14251.4399	0.0031
6	0	6	5	1	5	14292.1520	-0.0016
10	4	6	10	3	7	14426.1547	0.0066
13	1	12	13	0	13	14562.9530	-0.0071
9	4	5	9	3	6	14715.3420	-0.0023
3	2	1	2	1	2	14750.0497	-0.0019
8	4	4	8	3	5	14913.2619	-0.0054
14	3	12	14	2	13	15017.6723	-0.0043
7	4	3	7	3	4	15039.6864	-0.0018

Table S6: Observed transition frequencies (in MHz) for (Z)-$CHFCFCF_3$

J'	K_a'	K_c'	J''	K_a''	K_c''	Observed	Obs - Calc
9	2	7	8	3	6	15093.0793	-0.0048
6	1	6	5	1	5	15094.7892	-0.0038
13	4	10	12	5	7	15109.6673	-0.0242
6	4	2	6	3	3	15115.1019	-0.0005
8	4	5	8	3	6	15138.9757	0.0220
7	4	4	7	3	5	15145.6382	-0.0098
9	4	6	9	3	7	15147.0405	-0.0130
5	4	1	5	3	2	15156.9147	-0.0023
6	4	3	6	3	4	15158.4735	-0.0044
11	3	8	10	4	7	15164.5048	-0.0047
5	4	2	5	3	3	15171.6088	-0.0034
4	4	0	4	3	1	15178.1836	0.0043
10	4	7	10	3	8	15181.1839	-0.0056
4	4	1	4	3	2	15181.8896	-0.0047
11	4	8	11	3	9	15254.5664	-0.0009
6	0	6	5	0	5	15364.6338	-0.0004
12	4	9	12	3	10	15381.4544	-0.0002
11	2	10	10	3	7	15402.3124	0.0086
13	2	12	13	1	13	15436.1801	-0.0030
13	4	9	12	5	8	15520.3246	-0.0022
13	4	10	13	3	11	15576.1480	0.0124
6	2	5	5	2	4	15704.4625	-0.0044
6	5	1	5	5	0	15794.0245	0.0008
6	5	2	5	5	1		
6	4	3	5	4	2	15805.3520	0.0026
6	4	2	5	4	1	15806.0069	-0.0045
6	3	4	5	3	3	15818.4813	-0.0025
6	3	3	5	3	2	15847.8234	-0.0027
14	4	11	14	3	12	15851.8751	0.0162
15	3	13	15	2	14	15952.6163	-0.0052
14	1	13	14	0	14	16050.8827	-0.0107
6	2	4	5	2	3	16101.2406	0.0013
15	5	11	14	6	8	16141.9794	-0.0010
17	2	15	17	1	16	16164.7731	0.0105
6	1	6	5	0	5	16167.2737	0.0001
15	4	12	15	3	13	16219.8996	0.0076
6	1	5	5	1	4	16222.6815	-0.0035
12	3	10	11	4	7	16257.9068	0.0037
4	2	3	3	1	2	16443.6473	-0.0042
14	2	13	14	1	14	16664.2821	-0.0094
12	2	11	11	3	8	16854.1245	-0.0042
16	3	14	16	2	15	16972.7760	0.0227
7	0	7	6	1	6	17001.4948	0.0003

Table S6: Observed transition frequencies (in MHz) for (*Z*)-$CHFCFCF_3$

J'	K_a'	K_c'	J''	K_a''	K_c''	Observed	Obs - Calc
17	5	12	17	4	13	17040.6243	-0.0084
17	4	14	17	3	15	17263.7486	0.0163
8	1	7	7	2	6	17425.9649	-0.0020
15	1	14	15	0	15	17503.7397	-0.0092
7	1	7	6	1	6	17576.3094	0.0003
16	5	11	16	4	12	17664.9266	-0.0071
4	2	2	3	1	3	17789.1075	-0.0014
7	0	7	6	0	6	17804.1368	0.0030
18	2	16	18	1	17	17805.6341	0.0154
15	2	14	15	1	15	17924.2299	-0.0042
18	4	15	18	3	16	17946.5094	-0.0029
13	2	12	12	3	9	17984.2833	0.0021
17	3	15	17	2	16	18067.3350	-0.0092

Table S7: Observed transition frequencies (in MHz) for (Z)-$^{13}CHFCFCF_3$

J'	K_a'	K_c'	J''	K_a''	K_c''	Observed	Obs - Calc
1	1	0	1	0	1	2264.2276	0.0101
2	1	1	2	0	2	2469.1431	0.0030
3	1	2	3	0	3	2799.7909	0.0026
4	1	4	3	2	1	2882.7248	-0.0011
2	0	2	1	1	1	3136.7486	0.0023
4	1	3	4	0	4	3281.2567	0.0001
5	1	4	5	0	5	3939.8326	0.0019
1	1	1	0	0	0	4682.8307	0.0059
6	1	5	6	0	6	4794.4009	0.0017
5	1	5	4	2	2	4848.2065	0.0075
4	1	3	3	2	2	4864.1180	0.0012
2	1	2	1	1	1	5029.3867	0.0072
2	0	2	1	0	1	5208.8028	0.0081
2	1	1	1	1	0	5413.7199	0.0025
6	2	4	6	1	5	5427.3734	0.0066
7	2	5	7	1	6	5437.4391	0.0076
6	2	4	5	3	3	5480.2371	0.0081
5	2	3	5	1	4	5549.1040	0.0079
8	2	6	8	1	7	5616.9613	0.0075
4	2	2	4	1	3	5756.7285	0.0081
7	1	6	7	0	7	5847.7026	0.0020
8	3	6	7	4	3	5857.8952	0.0031
3	0	3	2	1	2	5889.0397	0.0056
9	2	7	9	1	8	5995.7317	0.0113
3	2	1	3	1	2	5999.5521	0.0103
2	2	0	2	1	1	6228.8953	0.0003
6	1	6	5	2	3	6583.0472	-0.0040
10	2	8	10	1	9	6596.2679	-0.0152
2	2	1	2	1	2	6792.6468	0.0011
7	2	6	6	3	3	7070.4032	0.0077
8	1	7	8	0	8	7082.0010	0.0017
3	2	2	3	1	3	7088.6122	0.0005
2	1	2	1	0	1	7101.4265	-0.0015
11	2	9	11	1	10	7431.5608	-0.0028
4	2	3	4	1	4	7486.9925	-0.0013
3	1	3	2	1	2	7536.3353	-0.0052
3	0	3	2	0	2	7781.6657	-0.0016
3	2	2	2	2	1	7832.3011	-0.0054
3	2	1	2	2	0	7882.9635	0.0014
5	1	4	4	2	3	7903.6733	-0.0012
5	2	4	5	1	5	7989.5689	-0.0017
3	1	2	2	1	1	8112.3154	-0.0001
9	1	8	9	0	9	8461.3066	-0.0020

Table S7: Observed transition frequencies (in MHz) for (Z)-^{13}CHFCFCF$_3$

J'	K_a'	K_c'	J''	K_a''	K_c''	Observed	Obs - Calc
7	2	5	6	3	4	8516.1744	0.0043
6	2	5	6	1	6	8596.9701	-0.0052
4	0	4	3	1	3	8671.4694	-0.0025
10	3	7	10	2	8	8700.6984	-0.0047
9	3	6	9	2	7	9101.5342	-0.0008
7	2	6	7	1	7	9307.9894	-0.0031
8	2	7	7	3	4	9387.7135	0.0005
3	1	3	2	0	2	9428.9706	-0.0030
8	3	5	8	2	6	9534.1961	0.0011
7	3	4	7	2	5	9942.6888	-0.0004
4	1	4	3	1	3	10034.7476	-0.0019
8	2	7	8	1	8	10119.0585	-0.0007
6	3	3	6	2	4	10284.3817	0.0019
4	0	4	3	0	3	10318.7769	-0.0014
4	3	2	3	3	1	10467.0398	-0.0002
4	3	1	3	3	0	10470.2304	0.0153
5	3	2	5	2	3	10536.5776	0.0004
4	2	2	3	2	1	10557.4262	0.0010
4	3	1	4	2	2	10698.2582	-0.0136
3	3	0	3	2	1	10785.4991	0.0172
4	1	3	3	1	2	10800.2521	0.0055
3	3	1	3	2	2	10848.3602	-0.0030
5	3	3	5	2	4	10949.1187	-0.0027
6	1	5	5	2	4	11001.9759	-0.0077
9	2	8	9	1	9	11024.0915	0.0025
7	3	5	7	2	6	11236.4052	0.0030
5	0	5	4	1	4	11450.8311	-0.0066
11	1	10	11	0	11	11465.5148	0.0012
9	2	8	8	3	5	11555.6142	-0.0051
2	2	1	1	1	0	11629.8565	0.0005
4	1	4	3	0	3	11682.0487	-0.0072
9	3	7	9	2	8	11816.7498	-0.0031
2	2	0	1	1	1	11834.7829	0.0014
5	1	5	4	1	4	12522.8996	0.0011
11	3	9	11	2	10	12772.5601	0.0056
5	0	5	4	0	4	12814.1121	-0.0032
5	2	4	4	2	3	13025.4617	-0.0136
5	1	4	4	1	3	13472.6828	-0.0065
5	1	5	4	0	4	13886.1802	0.0041
3	2	2	2	1	1	14048.4468	0.0016
6	0	6	5	1	5	14197.1525	-0.0024
6	4	2	6	3	3	15088.6941	-0.0047
6	1	6	5	0	5	16071.9752	-0.0027

Table S7: Observed transition frequencies (in MHz) for (Z)-$^{13}CHFCFCF_3$

J'	K_a'	K_c'	J''	K_a''	K_c''	Observed	Obs - Calc
4	2	3	3	1	2	16369.2575	-0.0039
7	0	7	6	1	6	16890.5395	-0.0047
7	0	7	6	0	6	17693.3161	0.0097
4	2	2	3	1	3	17709.4479	-0.0010

Table S8: Observed transition frequencies (in MHz) for (Z)-$CHF^{13}CFCF_3$

J'	K_a'	K_c'	J''	K_a''	K_c''	Observed	Obs - Calc
5	2	4	4	3	1	2192.5635	-0.0053
2	1	1	2	0	2	2473.8827	0.0032
3	1	2	3	0	3	2805.5668	0.0049
4	1	4	3	2	1	2926.0024	-0.0014
2	0	2	1	1	1	3162.1624	0.0027
4	1	3	4	0	4	3288.5762	0.0048
5	1	4	5	0	5	3949.2846	0.0039
1	1	1	0	0	0	4700.8775	0.0066
6	2	5	5	3	2	4710.5797	0.0095
6	1	5	6	0	6	4806.6131	0.0039
5	1	5	4	2	2	4903.8761	0.0017
4	1	3	3	2	2	4913.3620	0.0022
2	1	2	1	1	1	5057.8228	0.0067
2	0	2	1	0	1	5237.7527	0.0027
2	1	1	1	1	0	5443.2988	0.0008
7	2	5	7	1	6	5447.3976	0.0091
5	2	3	5	1	4	5558.2999	0.0088
8	2	6	8	1	7	5628.2337	0.0086
4	2	2	4	1	3	5766.2380	0.0076
7	1	6	7	0	7	5863.2424	0.0072
3	0	3	2	1	2	5929.3201	0.0074
8	3	6	7	4	3	5947.6447	0.0159
9	2	7	9	1	8	6009.0263	-0.0072
3	2	1	3	1	2	6009.6110	0.0022
8	3	5	7	4	4	6184.8532	-0.0111
10	2	8	10	1	9	6612.3999	-0.0044
6	1	6	5	2	3	6650.3058	-0.0046
2	2	1	2	1	2	6804.9880	0.0006
3	2	2	3	1	3	7101.8449	0.0013
2	1	2	1	0	1	7133.4048	-0.0016
7	2	6	6	3	3	7151.2321	-0.0019
4	2	3	4	1	4	7501.4255	-0.0015
3	1	3	2	1	2	7578.9596	-0.0037
3	0	3	2	0	2	7824.9661	-0.0030
3	2	2	2	2	1	7875.8236	0.0040
3	2	1	2	2	0	7926.6801	-0.0062
5	1	4	4	2	3	7968.6226	-0.0060
5	2	4	5	1	5	8005.5198	-0.0011
3	1	2	2	1	1	8156.6478	-0.0037
12	3	9	12	2	10	8246.9913	-0.0015
13	3	10	13	2	11	8285.9831	0.0018
11	3	8	11	2	9	8405.3612	-0.0115
9	1	8	9	0	9	8484.5308	-0.0081

Table S8: Observed transition frequencies (in MHz) for (Z)-$CHF^{13}CFCF_3$

J'	K_a'	K_c'	J''	K_a''	K_c''	Observed	Obs - Calc
7	2	5	6	3	4	8602.7319	0.0029
6	2	5	6	1	6	8614.7533	-0.0032
10	3	7	10	2	8	8713.7218	-0.0056
4	0	4	3	1	3	8726.6286	-0.0034
9	3	6	9	2	7	9115.1502	0.0073
7	2	6	7	1	7	9327.9059	-0.0002
3	1	3	2	0	2	9474.6171	-0.0026
8	3	5	8	2	6	9548.8185	-0.0026
7	3	4	7	2	5	9958.5192	-0.0002
10	1	9	10	0	10	9965.8356	-0.0002
4	1	4	3	1	3	10091.5464	0.0225
8	2	7	8	1	8	10141.3822	-0.0016
6	3	3	6	2	4	10301.3639	-0.0013
4	0	4	3	0	3	10376.2834	0.0008
4	2	3	3	2	2	10491.1070	-0.0003
5	3	2	5	2	3	10554.5019	0.0054
4	2	2	3	2	1	10615.9245	0.0109
4	1	3	3	1	2	10859.2932	0.0011
6	3	4	6	2	5	11082.5751	-0.0009
6	1	5	5	2	4	11082.7587	-0.0083
7	3	5	7	2	6	11257.1496	0.0087
8	3	6	8	2	7	11505.6494	0.0027
5	0	5	4	1	4	11520.7699	-0.0049
2	2	1	1	1	0	11670.0556	-0.0067
4	1	4	3	0	3	11741.1757	0.0012
8	2	6	7	3	5	11781.6011	0.0003
9	3	7	9	2	8	11839.7369	-0.0053
2	2	0	1	1	1	11875.6128	-0.0004
10	3	8	10	2	9	12268.7506	0.0040
5	1	5	4	1	4	12593.7862	0.0020
11	3	9	11	2	10	12799.0993	0.0036
5	0	5	4	0	4	12885.6642	-0.0026
5	2	4	4	2	3	13097.8732	-0.0049
5	3	3	4	3	2	13165.0028	0.0007
5	3	2	4	3	1	13176.1170	0.0101
5	2	3	4	2	2	13338.4404	0.0036
5	1	4	4	1	3	13546.3692	-0.0069
5	1	5	4	0	4	13958.6798	0.0036
3	2	2	2	1	1	14102.5751	-0.0087
6	0	6	5	1	5	14281.6765	-0.0022
3	2	1	2	1	2	14744.4745	-0.0090
8	4	4	8	3	5	14913.6019	0.0019
6	2	4	5	2	3	16090.4081	0.0177

Table S8: Observed transition frequencies (in MHz) for (Z)-$CHF^{13}CFCF_3$

J'	K_a'	K_c'	J''	K_a''	K_c''	Observed	Obs - Calc
6	1	6	5	0	5	16157.8809	-0.0012
4	2	3	3	1	2	16437.0329	-0.0067
7	0	7	6	1	6	16989.4335	-0.0043
4	2	2	3	1	3	17781.4310	-0.0028
7	0	7	6	0	6	17792.6213	-0.0106

Table S9: Observed transition frequencies (in MHz) for (Z)-$CHFCF^{13}CF_3$

J'	K_a'	K_c'	J''	K_a''	K_c''	Observed	Obs - Calc
1	1	0	1	0	1	2270.9379	0.0032
2	1	1	2	0	2	2475.6737	0.0027
2	0	2	1	1	1	3150.3703	0.0015
4	1	3	4	0	4	3286.7489	0.0019
5	1	4	5	0	5	3944.3333	0.0020
6	2	5	5	3	2	4671.6046	-0.0057
1	1	1	0	0	0	4699.8835	0.0060
6	1	5	6	0	6	4797.6515	0.0035
5	1	5	4	2	2	4880.9694	0.0076
4	1	3	3	2	2	4884.1150	0.0009
2	1	2	1	1	1	5049.9314	0.0102
2	0	2	1	0	1	5229.2676	0.0038
2	1	1	1	1	0	5434.0070	0.0069
6	2	4	6	1	5	5445.2600	0.0101
7	2	5	7	1	6	5453.3497	0.0083
6	2	4	5	3	3	5505.0778	0.0128
5	2	3	5	1	4	5568.3618	0.0100
8	2	6	8	1	7	5630.3165	0.0124
4	2	2	4	1	3	5776.7938	0.0107
7	1	6	7	0	7	5849.6494	0.0033
8	3	6	7	4	3	5891.7867	-0.0063
3	0	3	2	1	2	5912.9638	0.0078
9	2	7	9	1	8	6005.9564	0.0000
3	2	1	3	1	2	6019.9596	0.0013
2	2	0	2	1	1	6249.3782	0.0002
10	2	8	10	1	9	6602.9108	-0.0122
2	2	1	2	1	2	6812.7952	-0.0018
8	1	7	8	0	8	7082.9052	0.0027
3	2	2	3	1	3	7108.5340	-0.0012
7	2	6	6	3	3	7109.6329	0.0000
2	1	2	1	0	1	7128.8169	0.0006
11	2	9	11	1	10	7434.3190	0.0029
4	2	3	4	1	4	7506.5990	-0.0021
3	1	3	2	1	2	7567.1823	-0.0043
3	2	2	2	2	1	7862.9270	0.0021
3	2	1	2	2	0	7913.3631	0.0053
5	1	4	4	2	3	7933.8008	-0.0058
5	2	4	5	1	5	8008.7721	-0.0019
3	1	2	2	1	1	8142.7790	0.0014
13	3	10	13	2	11	8292.6694	0.0105
11	3	8	11	2	9	8423.9998	-0.0024
9	1	8	9	0	9	8461.7639	0.0147
6	2	5	6	1	6	8615.6933	-0.0061

Table S9: Observed transition frequencies (in MHz) for (Z)-CHFCF^{13}CF$_3$

J'	K_a'	K_c'	J''	K_a''	K_c''	Observed	Obs - Calc
4	0	4	3	1	3	8705.8998	-0.0025
10	3	7	10	2	8	8735.2864	-0.0029
9	3	6	9	2	7	9137.7567	0.0099
7	2	6	7	1	7	9326.1839	-0.0046
8	2	7	7	3	4	9438.5671	-0.0007
3	1	3	2	0	2	9466.7393	0.0002
8	3	5	8	2	6	9570.9505	0.0008
7	3	4	7	2	5	9979.1984	-0.0013
4	1	4	3	1	3	10075.9295	-0.0051
8	2	7	8	1	8	10136.7212	0.0011
6	3	3	6	2	4	10320.2237	-0.0004
4	0	4	3	0	3	10360.1327	-0.0002
4	2	3	3	2	2	10473.9965	-0.0041
5	3	2	5	2	3	10571.6787	0.0017
4	2	2	3	2	1	10597.7563	-0.0083
4	3	1	4	2	2	10732.7797	-0.0002
3	3	0	3	2	1	10819.6302	-0.0023
4	1	3	3	1	2	10840.9329	-0.0069
4	3	2	4	2	3	10915.9987	-0.0025
5	3	3	5	2	4	10982.5706	-0.0017
6	1	5	5	2	4	11042.3974	0.0099
6	3	4	6	2	5	11095.5286	0.0019
7	3	5	7	2	6	11268.7253	-0.0073
11	1	10	11	0	11	11467.5078	-0.0025
5	0	5	4	1	4	11496.0760	-0.0005
8	3	6	8	2	7	11515.3676	-0.0045
9	2	8	8	3	5	11618.8377	0.0108
2	2	1	1	1	0	11670.6869	0.0084
8	2	6	7	3	5	11721.4734	-0.0030
4	1	4	3	0	3	11730.1657	0.0004
9	3	7	9	2	8	11847.0628	0.0024
2	2	0	1	1	1	11875.4211	0.0033
5	1	5	4	1	4	12574.4652	0.0057
11	3	9	11	2	10	12800.0019	-0.0108
5	0	5	4	0	4	12866.1111	0.0022
5	2	4	4	2	3	13076.6335	0.0010
5	3	3	4	3	2	13143.2079	0.0043
5	3	2	4	3	1	13154.1472	-0.0116
5	1	4	4	1	3	13523.6950	0.0019
5	1	5	4	0	4	13944.4935	0.0015
3	2	2	2	1	1	14099.6028	-0.0005
6	0	6	5	1	5	14253.5086	-0.0050
3	2	1	2	1	2	14738.8491	-0.0053

Table S9: Observed transition frequencies (in MHz) for (Z)-$CHFCF^{13}CF_3$

J'	K_a'	K_c'	J''	K_a''	K_c''	Observed	Obs - Calc
6	1	6	5	0	5	16140.2715	0.0007
4	2	3	3	1	2	16430.8255	-0.0009
7	0	7	6	1	6	16958.2367	-0.0080
7	0	7	6	0	6	17766.6129	-0.0060
4	2	2	3	1	3	17769.4284	-0.0040

Table S10: Observed transition frequencies (in MHz) for Ar-(E)-$CHFCFCF_3$

J'	K_a'	K_c'	J''	K_a''	K_c''	Observed	Obs - Calc
3	2	1	3	1	2	2003.4362	-0.0034
7	2	5	7	1	6	2019.7431	-0.0016
2	2	0	2	1	1	2108.0898	0.0010
6	1	5	6	0	6	2228.8836	-0.0048
1	1	1	0	0	0	2232.1138	0.0013
2	0	2	1	1	0	2255.3478	-0.0017
8	2	6	8	1	7	2258.3477	-0.0024
1	1	0	0	0	0	2332.8619	0.0090
2	0	2	1	1	1	2356.0929	0.0030
4	2	3	3	3	0	2367.8264	0.0035
9	3	7	9	2	7	2393.6200	0.0158
2	2	1	2	1	2	2400.1846	-0.0011
5	3	3	4	4	0	2460.6552	0.0135
5	3	2	4	4	1	2478.2709	0.0137
4	2	2	3	3	1	2513.9172	0.0043
3	2	2	3	1	3	2557.2839	-0.0014
3	2	1	3	1	3	2607.2102	-0.0003
9	2	7	9	1	8	2619.8004	0.0009
3	1	2	2	2	1	2644.6811	0.0028
8	3	6	8	2	6	2747.5459	0.0132
4	2	3	4	1	4	2769.2544	-0.0041
7	1	6	7	0	7	2797.0788	-0.0040
10	3	7	10	2	8	2830.8301	0.0002
9	3	6	9	2	7	2878.5932	0.0006
11	3	8	11	2	9	2893.8026	0.0058
4	2	2	4	1	4	2914.6964	-0.0133
2	1	2	1	1	1	2964.7902	-0.0029
8	3	5	8	2	6	3008.6545	0.0004
5	2	4	5	1	5	3036.6208	-0.0036
2	0	2	1	0	1	3055.4167	-0.0029
12	3	9	12	2	10	3087.0073	-0.0049
10	2	8	10	1	9	3102.3704	-0.0001
2	1	1	1	1	0	3166.2652	-0.0021
7	3	4	7	2	5	3184.8581	0.0005
4	1	4	3	2	1	3303.7219	-0.0004
6	2	5	6	1	6	3358.4058	-0.0040
5	2	3	5	1	5	3359.1950	-0.0007
6	3	3	6	2	4	3368.5184	-0.0020
13	3	10	13	2	11	3420.8092	0.0075
8	1	7	8	0	8	3424.4656	-0.0042
5	3	2	5	2	3	3526.6490	0.0026
4	3	2	4	2	2	3635.2533	-0.0060
4	3	1	4	2	2	3639.7033	-0.0022

Table S10: Observed transition frequencies (in MHz) for Ar-(E)-$CHFCFCF_3$

J'	K_a'	K_c'	J''	K_a''	K_c''	Observed	Obs - Calc
3	0	3	2	1	1	3647.5056	-0.0037
2	1	2	1	0	1	3664.1201	-0.0028
11	2	9	11	1	10	3689.4792	0.0026
3	3	1	3	2	1	3704.5154	-0.0037
3	3	0	3	2	1	3705.1532	-0.0047
7	2	6	7	1	7	3731.6973	-0.0040
15	4	11	15	3	12	3749.6809	-0.0012
3	3	1	3	2	2	3754.4404	-0.0039
3	3	0	3	2	2	3755.0713	-0.0118
14	4	10	14	3	11	3755.1998	0.0048
4	3	2	4	2	3	3780.7075	-0.0029
4	3	1	4	2	3	3785.1546	-0.0020
5	3	3	5	2	4	3831.6321	-0.0035
5	3	2	5	2	4	3849.2148	-0.0029
5	2	4	4	3	1	3855.6729	0.0041
13	4	9	13	3	10	3880.0136	-0.0063
14	3	11	14	2	12	3893.8303	-0.0004
6	3	4	6	2	5	3916.2387	-0.0015
3	0	3	2	1	2	3949.7220	-0.0018
2	1	1	1	0	1	3966.3395	0.0021
6	3	3	6	2	5	3967.9332	0.0092
7	3	5	7	2	6	4042.7952	-0.0005
7	4	4	6	5	1	4059.1217	-0.0104
6	3	3	5	4	2	4061.8949	0.0028
7	4	3	6	5	2	4064.5441	-0.0032
9	1	8	9	0	9	4079.7037	0.0003
12	4	8	12	3	9	4089.6413	-0.0032
8	2	7	8	1	8	4151.6039	-0.0033
7	3	4	7	2	6	4167.7505	-0.0051
5	2	3	4	3	1	4178.2420	0.0020
17	4	13	17	3	14	4181.7352	-0.0067
5	2	3	4	3	2	4182.6872	0.0010
8	3	6	8	2	7	4217.9983	-0.0111
4	1	3	3	2	1	4306.5798	-0.0029
11	4	7	11	3	8	4342.1811	-0.0003
12	2	10	12	1	11	4351.4548	0.0007
4	1	3	3	2	2	4356.5083	0.0004
3	1	3	2	1	2	4441.0892	-0.0039
9	3	7	9	2	8	4446.4135	0.0004
8	3	5	8	2	7	4479.1220	-0.0089
15	3	12	15	2	13	4490.0006	0.0130
10	4	7	10	3	7	4504.1563	-0.0055
3	0	3	2	0	2	4558.4214	-0.0057

Table S10: Observed transition frequencies (in MHz) for Ar-(E)-$CHFCFCF_3$

J'	K_a'	K_c'	J''	K_a''	K_c''	Observed	Obs - Calc
10	4	6	10	3	7	4595.4019	-0.0024
3	2	2	2	2	1	4598.1832	-0.0095
5	1	5	4	2	3	4604.1955	-0.0055
9	2	8	9	1	9	4611.6494	-0.0028
3	2	1	2	2	0	4637.9905	-0.0097
18	5	13	18	4	14	4683.1854	0.0150
7	2	5	7	1	7	4714.6113	0.0120
10	1	9	10	0	10	4736.8891	0.0034
9	4	5	9	3	6	4815.5747	0.0013
17	5	12	17	4	13	4885.9592	-0.0051
4	0	4	3	1	2	4940.1432	-0.0041
8	4	5	8	3	5	4968.2144	-0.0027
8	4	4	8	3	5	4984.1487	-0.0005
3	1	3	2	0	2	5049.7944	-0.0020
13	2	11	13	1	12	5052.7810	0.0024
11	3	9	11	2	10	5067.9494	-0.0003
7	4	4	7	3	4	5093.5028	-0.0051
7	4	3	7	3	4	5098.9037	-0.0021
10	2	9	10	1	10	5104.4834	-0.0020
6	4	3	6	3	3	5167.4837	-0.0004
6	4	2	6	3	3	5168.9679	-0.0042
16	5	11	16	4	12	5171.4872	-0.0044
16	3	13	16	2	14	5178.8812	-0.0114
5	4	2	5	3	2	5207.2439	-0.0132
5	4	1	5	3	2	5207.5568	0.0000
7	4	4	7	3	5	5218.4683	0.0004
6	4	3	6	3	4	5219.1667	-0.0011
6	4	2	6	3	4	5220.6403	-0.0155
7	4	3	7	3	5	5223.8674	0.0016
5	4	2	5	3	3	5224.8328	-0.0064
5	4	1	5	3	3	5225.1415	0.0026
4	4	0	4	3	1	5226.6630	0.0004
8	4	5	8	3	6	5229.3375	-0.0011
4	4	1	4	3	2	5231.0620	-0.0135
8	4	4	8	3	6	5245.2802	0.0096
9	4	6	9	3	7	5260.1193	0.0079
2	2	1	1	1	0	5264.2324	-0.0061
2	2	0	1	1	0	5274.3642	0.0081
6	2	5	5	3	2	5301.2246	-0.0007
10	4	7	10	3	8	5320.0114	-0.0083
2	2	1	1	1	1	5364.9913	0.0125
2	2	0	1	1	1	5375.0944	-0.0021
10	4	6	10	3	8	5411.2527	-0.0095

Table S10: Observed transition frequencies (in MHz) for Ar-(E)-$CHFCFCF_3$

J'	K_a'	K_c'	J''	K_a''	K_c''	Observed	Obs - Calc
11	4	8	11	3	9	5418.1817	0.0011
12	3	10	12	2	11	5457.0346	0.0053
6	1	6	5	2	3	5469.4663	0.0044
15	5	10	15	4	11	5493.1404	-0.0002
4	0	4	3	1	3	5543.9186	0.0006
7	3	5	6	4	2	5556.4074	0.0006
12	4	9	12	3	10	5562.5993	0.0049
11	4	7	11	3	9	5604.6771	0.0102
8	4	5	7	5	2	5622.0047	0.0015
11	2	10	11	1	11	5622.6898	0.0003
8	4	4	7	5	3	5638.0421	0.0034
3	1	2	2	0	2	5653.5665	-0.0008
9	5	5	8	6	2	5654.5297	-0.0057
7	3	4	6	4	3	5682.8596	0.0047
13	4	10	13	3	11	5759.3157	0.0056
14	2	12	14	1	13	5761.3052	0.0001
14	5	9	14	4	10	5806.6803	0.0110
13	3	11	13	2	12	5891.7668	0.0015
6	2	4	5	3	2	5900.6068	-0.0221
4	1	4	3	1	3	5910.9316	-0.0012
6	2	4	5	3	3	5918.2110	0.0000
5	1	4	4	2	2	5952.0427	-0.0030
12	1	11	12	0	12	6006.5494	0.0021
14	4	11	14	3	12	6011.8490	0.0011
13	5	9	13	4	9	6017.6893	-0.0231
4	0	4	3	0	3	6035.2893	0.0019
13	5	8	13	4	9	6079.0470	-0.0012
5	1	4	4	2	3	6097.4977	0.0009
5	0	5	4	1	3	6116.0900	0.0064
4	2	3	3	2	2	6122.9033	-0.0028
4	3	2	3	3	1	6149.1789	0.0066
4	3	1	3	3	0	6152.9790	-0.0006
12	2	11	12	1	12	6159.4754	-0.0061
4	2	2	3	2	1	6218.4298	-0.0022
12	5	7	12	4	8	6293.7535	0.0065
4	1	3	3	1	2	6310.0214	-0.0009
7	1	7	6	2	4	6319.4025	0.0001
15	4	12	15	3	13	6320.9220	0.0038
14	3	12	14	2	13	6365.2630	0.0019
4	1	4	3	0	3	6402.2999	-0.0022
11	5	6	11	4	7	6449.9360	-0.0036
15	2	13	15	1	14	6455.2465	-0.0014
10	5	6	10	4	6	6552.2042	-0.0073

Table S10: Observed transition frequencies (in MHz) for Ar-(E)-$CHFCFCF_3$

J'	K_a'	K_c'	J''	K_a''	K_c''	Observed	Obs - Calc
10	5	5	10	4	6	6556.7670	-0.0005
12	5	8	12	4	9	6615.1298	0.0165
13	1	12	13	0	13	6615.4813	0.0016
11	5	7	11	4	8	6624.5116	0.0005
9	5	4	9	4	5	6626.7039	0.0018
11	5	6	11	4	8	6636.4370	0.0112
10	5	6	10	4	7	6643.4569	0.0028
19	6	13	19	5	14	6646.1169	0.0045
10	5	5	10	4	7	6647.9986	-0.0114
14	5	10	14	4	11	6658.2824	0.0034
9	5	5	9	4	6	6665.6105	-0.0011
9	5	4	9	4	6	6667.1510	-0.0014
8	5	4	8	4	4	6670.7239	0.0053
8	5	3	8	4	4	6671.1614	-0.0021
7	2	6	6	3	3	6682.5827	-0.0006
16	4	13	16	3	14	6684.4574	0.0048
8	5	4	8	4	5	6686.6451	-0.0055
3	2	2	2	1	1	6696.1608	-0.0031
7	5	2	7	4	3	6698.8493	-0.0010
7	5	3	7	4	4	6704.1403	-0.0044
13	2	12	13	1	13	6709.1667	0.0016
6	5	1	6	4	2	6715.7487	0.0061
6	5	2	6	4	3	6717.2033	-0.0101
5	5	0	5	4	1	6725.7444	0.0066
5	5	1	5	4	2	6726.0277	-0.0082
15	5	11	15	4	12	6729.3857	0.0123
3	2	1	2	1	1	6746.0858	-0.0033
16	5	12	16	4	13	6845.2197	-0.0030
15	3	13	15	2	14	6869.9880	-0.0012
7	1	7	6	2	5	6918.8229	0.0170
3	2	2	2	1	2	6998.3758	-0.0025
8	1	8	7	2	5	7005.6633	0.0014
18	6	12	18	5	13	7011.6220	-0.0010
17	5	13	17	4	14	7012.8013	-0.0003
3	2	1	2	1	2	7048.2989	-0.0047
8	3	6	7	4	3	7091.5143	-0.0007
17	4	14	17	3	15	7097.9516	0.0090
5	0	5	4	1	4	7118.9418	-0.0022
16	2	14	16	1	15	7124.8904	-0.0091
6	0	6	5	1	4	7169.8699	0.0013
9	4	6	8	5	3	7191.1814	-0.0048
14	1	13	14	0	14	7211.3273	-0.0081
10	5	6	9	6	3	7222.4349	0.0052

Table S10: Observed transition frequencies (in MHz) for Ar-(E)-$CHFCFCF_3$

J'	K_a'	K_c'	J''	K_a''	K_c''	Observed	Obs - Calc
10	5	5	9	6	4	7227.0045	-0.0127
9	4	5	8	5	4	7232.0796	-0.0019
8	3	5	7	4	4	7358.0360	0.0017
5	1	5	4	1	4	7373.4616	0.0021
16	3	14	16	2	15	7398.5892	0.0125
4	1	3	3	0	3	7405.1595	-0.0030
5	0	5	4	0	4	7485.9593	0.0006
6	1	5	5	2	3	7532.8598	0.0028
16	6	10	16	5	11	7580.4043	0.0031
5	2	4	4	2	3	7640.8279	0.0024
7	2	5	6	3	3	7665.4724	-0.0090
5	4	2	4	4	1	7685.5133	-0.0010
5	4	1	4	4	0	7685.7786	-0.0020
5	3	3	4	3	2	7691.7519	0.0013
5	3	2	4	3	1	7704.8890	0.0026
7	2	5	6	3	4	7717.1669	0.0019
5	1	5	4	0	4	7740.4723	-0.0019
17	2	15	17	1	16	7769.6030	-0.0094
15	6	9	15	5	10	7771.0280	0.0020
15	1	14	15	0	15	7797.8960	-0.0020
5	2	3	4	2	2	7817.9451	-0.0004
6	1	5	5	2	4	7855.4287	0.0004
5	1	4	4	1	3	7863.8942	-0.0008
14	6	8	14	5	9	7909.2897	0.0104
10	1	10	9	2	7	7930.3037	-0.0154
17	6	12	17	5	13	7942.0648	-0.0033
17	3	15	17	2	16	7944.3968	-0.0216
16	6	11	16	5	12	7956.4213	0.0034
8	2	7	7	3	4	7972.4084	-0.0030
8	1	8	7	2	6	7988.5630	0.0031
13	6	7	13	5	8	8007.4686	-0.0007
14	6	9	14	5	10	8025.0752	0.0007
13	6	8	13	5	9	8065.4863	0.0003
12	6	7	12	5	7	8075.4604	-0.0229
4	2	3	3	1	2	8076.4201	-0.0003
12	6	6	12	5	7	8076.7241	-0.0032
8	2	7	7	3	5	8097.3604	-0.0110
12	6	7	12	5	8	8103.6825	0.0013
7	0	7	6	1	5	8111.6048	0.0124
11	6	5	11	5	6	8125.6528	-0.0019
11	6	6	11	5	7	8137.1423	-0.0067
10	6	4	10	5	5	8160.3518	0.0025
10	6	5	10	5	6	8164.7807	0.0003

Table S10: Observed transition frequencies (in MHz) for Ar-(E)-$CHFCFCF_3$

J'	K_a'	K_c'	J''	K_a''	K_c''	Observed	Obs - Calc
9	6	3	9	5	4	8184.9595	0.0054
9	6	4	9	5	5	8186.4584	-0.0050
8	6	2	8	5	3	8202.2617	-0.0008
8	6	3	8	5	4	8202.6936	-0.0074
7	6	1	7	5	2	8214.2534	0.0002
7	6	2	7	5	3		
4	2	2	3	1	2	8221.8666	-0.0050
6	6	1	6	5	2	8222.1715	-0.0032
6	6	0	6	5	1		
3	3	1	2	2	0	8342.5150	-0.0043
3	3	0	2	2	0	8343.1516	-0.0066
3	3	1	2	2	1	8352.6340	-0.0030
3	3	0	2	2	1	8353.2732	-0.0026
9	3	7	8	4	4	8602.2385	0.0002
6	0	6	5	1	5	8663.1645	0.0001
4	2	3	3	1	3	8680.1849	-0.0064
10	4	7	9	5	4	8763.9371	0.0074
11	5	7	10	6	4	8798.8500	-0.0048
11	5	6	10	6	5	8810.8987	0.0042
4	2	2	3	1	3	8825.6398	-0.0026
6	1	6	5	1	5	8828.6533	-0.0043
10	4	6	9	5	5	8856.7134	0.0003
6	0	6	5	0	5	8917.6791	-0.0008
17	1	16	17	0	17	8954.2463	0.0144
9	1	9	8	2	7	9007.4489	-0.0041
7	1	6	6	2	4	9014.2365	-0.0205
6	1	6	5	0	5	9083.1758	0.0027
9	3	6	8	4	4	9087.2301	0.0035
9	3	6	8	4	5	9103.1503	-0.0083
9	2	8	8	3	5	9139.9634	-0.0110
6	2	5	5	2	4	9150.4393	-0.0036
6	5	2	5	5	1	9220.5578	-0.0038
6	5	1	5	5	0		
6	4	3	5	4	2	9229.3750	-0.0012
6	4	2	5	4	1	9230.5648	0.0002
5	1	4	4	0	4	9233.7648	-0.0052
6	3	4	5	3	3	9235.0503	0.0026
6	3	3	5	3	2	9269.1446	-0.0047
17	7	10	17	6	11	9358.3756	0.0018
18	7	12	18	6	13	9373.1367	-0.0199
6	1	5	5	1	4	9398.7582	0.0013
9	2	8	8	3	6	9401.1067	0.0109
5	2	4	4	1	3	9407.2241	0.0006

Table S10: Observed transition frequencies (in MHz) for Ar-(E)-$CHFCFCF_3$

J'	K_a'	K_c'	J''	K_a''	K_c''	Observed	Obs - Calc
6	2	4	5	2	3	9427.2777	0.0024
17	7	11	17	6	12	9431.7536	0.0048
8	2	6	7	3	4	9442.8867	-0.0014
16	7	9	16	6	10	9450.2928	0.0011
16	7	10	16	6	11	9487.2448	-0.0169
15	7	8	15	6	9	9519.6549	-0.0050
15	7	9	15	6	10	9537.1959	-0.0060
8	2	6	7	3	5	9567.8471	-0.0010
14	7	7	14	6	8	9572.5711	-0.0064
14	7	8	14	6	9	9580.3584	-0.0080
13	7	6	13	6	7	9613.2799	0.0031
7	1	6	6	2	5	9613.6546	-0.0060
13	7	7	13	6	8	9616.4833	-0.0012
12	7	5	12	6	6	9644.6793	0.0063
12	7	6	12	6	7	9645.8766	-0.0068
11	7	4	11	6	5	9668.8197	0.0089
11	7	5	11	6	6	9669.2235	0.0013
10	7	3	10	6	4	9687.1854	0.0037
10	7	4	10	6	5	9687.2979	-0.0068
9	7	2	9	6	3	9700.9463	0.0007
9	7	3	9	6	4		
8	7	1	8	6	2	9710.9734	-0.0070
8	7	2	8	6	3		
7	7	0	7	6	1	9718.0851	-0.0080
7	7	1	7	6	2		
5	2	3	4	1	3	9729.7875	-0.0073
4	3	2	3	2	1	9853.6835	-0.0077
4	3	1	3	2	1	9858.1351	-0.0024
4	3	2	3	2	2	9903.6112	-0.0053
4	3	1	3	2	2	9908.0609	-0.0019
10	3	8	9	4	5	10070.6035	-0.0086
7	0	7	6	1	6	10174.9961	0.0086
7	1	7	6	1	6	10277.2205	0.0047
11	4	8	10	5	5	10334.6814	-0.0116
7	0	7	6	0	6	10340.4903	0.0096
8	1	7	7	2	5	10369.3886	-0.0071
5	2	4	4	1	4	10410.0860	0.0021
12	5	7	11	6	6	10412.4292	0.0043
7	1	7	6	0	6	10442.7121	0.0032
7	2	6	6	2	5	10650.5005	-0.0068
6	2	5	5	1	4	10693.7695	-0.0020
5	2	3	4	1	4	10732.6492	-0.0060

Table S10: Observed transition frequencies (in MHz) for Ar-(E)-CHFCFCF$_3$

J'	K_a'	K_c'	J''	K_a''	K_c''	Observed	Obs - Calc
7	6	1	6	6	0	10755.4156	-0.0001
7	6	2	6	6	1		
7	3	4	6	3	3	10850.3422	0.0032
7	1	6	6	1	5	10908.6760	0.0007
10	3	7	9	4	6	10926.9133	-0.0071
7	2	5	6	2	4	11034.0044	0.0028
11	8	3	11	7	4	11186.2613	-0.0025
11	8	4	11	7	5		
9	2	7	8	3	5	11192.7806	-0.0026
10	8	2	10	7	3	11198.1211	-0.0016
10	8	3	10	7	4		
9	8	1	9	7	2	11207.1061	-0.0025
9	8	2	9	7	3		
8	8	1	8	7	2	11213.7380	-0.0074
8	8	0	8	7	1		
6	2	4	5	1	4	11293.1744	-0.0008
11	0	11	10	1	9	11300.2609	-0.0262
5	3	3	4	2	2	11327.0040	-0.0058
5	3	2	4	2	2	11344.5900	-0.0019
8	1	7	7	2	6	11352.2905	-0.0032
4	4	1	3	3	0	11379.5998	-0.0090
4	4	0	3	3	1	11380.2842	0.0031
9	2	7	8	3	6	11453.9014	-0.0032
5	3	3	4	2	3	11472.4615	0.0005
11	3	9	10	4	6	11473.2765	-0.0034
5	3	2	4	2	3	11490.0279	-0.0152
8	0	8	7	1	7	11659.5257	0.0005
8	1	8	7	1	7	11720.2524	-0.0088
8	1	8	7	0	7	11822.4876	-0.0017
8	2	7	7	2	6	12140.1656	-0.0015
6	2	5	5	1	5	12187.0645	-0.0029
8	3	6	7	3	5	12315.3823	0.0015
8	4	4	7	4	3	12336.7667	-0.0189
8	1	7	7	1	6	12389.1344	-0.0060
8	3	5	7	3	4	12451.5412	-0.0010
8	2	6	7	2	5	12627.7491	0.0034
13	9	5	13	8	6	12670.3684	-0.0048
13	9	4	13	8	5		
12	9	4	12	8	5	12683.8315	0.0059
12	9	3	12	8	4		
11	9	3	11	8	4	12694.5022	0.0077
11	9	2	11	8	3		

Table S10: Observed transition frequencies (in MHz) for Ar-(E)-$CHFCFCF_3$

J'	K_a'	K_c'	J''	K_a''	K_c''	Observed	Obs - Calc
10	9	2	10	8	3	12702.8210	0.0099
10	9	1	10	8	2		
9	9	1	9	8	2	12709.1732	0.0098
9	9	0	9	8	1		
6	3	4	5	2	3	12744.1101	-0.0019
12	3	10	11	4	7	12781.6976	0.0044
6	2	4	5	1	5	12786.4674	-0.0035
6	3	3	5	2	3	12795.7964	0.0007
11	3	8	10	4	7	12827.0162	0.0083
5	4	2	4	3	1	12912.1418	-0.0017
5	4	1	4	3	1	12912.4376	-0.0056
5	4	2	4	3	2	12916.5852	-0.0046
5	4	1	4	3	2	12916.8888	-0.0006
7	2	5	6	1	5	12928.4194	-0.0005
9	1	8	8	2	7	13052.1155	0.0011
6	3	4	5	2	4	13066.6807	-0.0025
6	3	3	5	2	4	13118.3634	-0.0036
9	0	9	8	1	8	13124.0138	-0.0044
7	1	6	6	0	6	13137.5641	0.0005
9	1	9	8	1	8	13159.0523	-0.0078
8	2	7	7	1	6	13177.0094	-0.0043
9	0	9	8	0	8	13184.7480	-0.0062
9	1	9	8	0	8	13219.7945	-0.0016
10	2	8	9	3	7	13356.1980	-0.0040
9	2	8	8	2	7	13619.1095	0.0043
9	1	8	8	1	7	13840.0069	0.0191
7	2	6	6	1	6	14008.9160	-0.0010
9	3	6	8	3	5	14071.3869	0.0111
7	3	5	6	2	4	14093.8971	-0.0023
9	2	8	8	1	7	14406.9841	0.0056
5	5	0	4	4	1	14411.5378	0.0035
5	5	1	4	4	0		
6	4	2	5	3	2	14438.1351	0.0137
6	4	3	5	3	3	14454.2285	0.0131
6	4	2	5	3	3	14455.7040	0.0006
10	0	10	9	1	9	14575.0068	-0.0042
10	1	10	9	0	9	14629.8246	0.0025
8	2	6	7	1	6	14647.4920	0.0016
7	3	5	6	2	5	14693.3052	0.0023
10	1	9	9	2	8	14700.2463	0.0018
7	3	4	6	2	5	14818.2614	-0.0016
7	2	5	6	1	6	14991.8075	-0.0076
10	2	9	9	2	8	15087.6033	-0.0100

Table S10: Observed transition frequencies (in MHz) for Ar-(E)-$CHFCFCF_3$

J'	K_a'	K_c'	J''	K_a''	K_c''	Observed	Obs - Calc
8	1	7	7	0	7	15186.2166	-0.0066
11	2	9	10	3	8	15253.2182	-0.0126
10	1	9	9	1	8	15267.2213	-0.0140
8	3	6	7	2	5	15375.2937	0.0153
8	3	5	7	2	5	15636.4125	0.0126
10	2	9	9	1	8	15654.6190	0.0149
8	2	7	7	1	7	15871.8773	0.0089
7	4	4	6	3	3	15943.8516	0.0048
6	5	2	5	4	1	15946.2916	0.0017
6	5	1	5	4	2	15946.6235	0.0166
7	4	3	6	3	3	15949.2492	0.0045
7	4	4	6	3	4	15995.5404	0.0099
7	4	3	6	3	4	16000.9391	0.0107
11	0	11	10	1	10	16017.4065	0.0029
11	1	11	10	1	10	16028.3637	0.0019
11	0	11	10	0	10	16037.1783	0.0055
11	1	11	10	0	10	16048.1309	0.0000
11	1	10	10	2	9	16293.7323	0.0045
8	3	6	7	2	6	16358.1894	0.0129
9	2	7	8	1	7	16459.7804	-0.0069
11	2	10	10	2	9	16546.5723	0.0064
9	3	7	8	2	6	16595.0479	0.0064
8	3	5	7	2	6	16619.3053	0.0074
11	2	10	10	1	9	16933.9479	0.0132
11	4	7	10	4	6	17077.9543	0.0076
9	3	6	8	2	6	17080.0476	0.0178
12	2	10	11	3	9	17121.8628	0.0009
9	1	8	8	0	8	17264.4611	0.0035
11	2	9	10	2	8	17268.2043	0.0016
11	3	8	10	3	7	17331.1746	0.0049
8	2	6	7	1	7	17342.3510	0.0060
8	4	5	7	3	4	17419.7640	0.0047
8	4	4	7	3	4	17435.6943	0.0030
6	6	1	5	5	0	17442.7462	0.0099
6	6	0	5	5	1		
12	0	12	11	1	11	17454.4992	-0.0006
12	1	12	11	0	11	17471.4491	0.0024
7	5	3	6	4	2	17479.0261	0.0066
7	5	2	6	4	3	17480.6229	0.0120
8	4	4	7	3	5	17560.6551	0.0037
10	3	8	9	2	7	17764.7807	0.0026
9	2	8	8	1	8	17770.7114	-0.0009
12	1	11	11	2	10	17838.3447	-0.0129

Table S10: Observed transition frequencies (in MHz) for Ar-(E)-$CHFCFCF_3$

J'	K_a'	K_c'	J''	K_a''	K_c''	Observed	Obs - Calc
9	3	7	8	2	7	18065.5262	0.0080

Table S11: Observed transition frequencies (in MHz) for Ar-(E)-^{13}CHFCFCF$_3$

J'	K_a'	K_c'	J''	K_a''	K_c''	Observed	Obs - Calc
4	1	4	3	0	3	6362.0580	0.0002
3	2	2	2	1	1	6642.5793	0.0018
5	0	5	4	1	4	7109.3084	-0.0001
5	1	5	4	0	4	7693.3631	-0.0001
6	1	5	5	2	4	7894.6242	0.0003
4	2	3	3	1	2	8014.5705	0.0020
3	3	1	2	2	0	8267.5950	-0.0002
3	3	0	2	2	1	8279.2921	0.0020
6	0	6	5	1	5	8643.5310	-0.0010
6	1	6	5	0	5	9030.9941	0.0002
5	2	4	4	1	3	9335.6654	-0.0009
4	3	2	3	2	1	9773.6541	0.0003
4	3	1	3	2	2	9832.7607	-0.0006
7	0	7	6	1	6	10144.6549	0.0000
7	1	7	6	0	6	10386.8377	-0.0005
6	2	5	5	1	4	10611.7080	-0.0005
5	3	3	4	2	2	11238.9706	0.0005
4	4	1	3	3	0	11275.9648	-0.0008
4	4	0	3	3	1	11276.7312	-0.0020
5	3	2	4	2	3	11415.9862	-0.0014
8	0	8	7	1	7	11618.8041	-0.0005
8	1	8	7	0	7	11763.3237	0.0005
7	2	6	6	1	5	11853.2956	-0.0005
6	3	4	5	2	3	12644.5716	-0.0007
5	4	2	4	3	1	12805.0963	-0.0005
5	4	1	4	3	2	12810.5156	-0.0006
6	3	3	5	2	4	13049.9922	0.0016
9	0	9	8	1	8	13073.6910	0.0004
8	2	7	7	1	6	13076.5557	0.0010
9	1	9	8	0	8	13156.9654	0.0005
7	3	5	6	2	4	13979.9361	0.0003
5	5	1	4	4	0	14278.7726	0.0014
5	5	0	4	4	1	14278.8120	-0.0012
6	4	3	5	3	2	14325.2199	-0.0008
6	4	2	5	3	3	14346.9895	0.0012
10	0	10	9	1	9	14515.9205	-0.0004
10	1	10	9	0	9	14562.6517	-0.0003
7	3	4	6	2	5	14762.2877	0.0001
6	5	2	5	4	1	15810.4549	0.0014
6	5	1	5	4	2	15810.8344	0.0007
7	4	4	6	3	3	15825.9866	-0.0006
7	4	3	6	3	4	15891.0611	-0.0011
11	1	11	10	0	10	15975.9598	0.0001

Table S11: Observed transition frequencies (in MHz) for Ar-(E)-^{13}CHFCFCF$_3$

J'	K_a'	K_c'	J''	K_a''	K_c''	Observed	Obs - Calc
6	6	1	5	5	0	17280.8524	-0.0001
6	6	0	5	5	1		

Table S12: Observed transition frequencies (in MHz) for Ar-(E)-$CHF^{13}CFCF_3$

J'	K_a'	K_c'	J''	K_a''	K_c''	Observed	Obs - Calc
4	1	4	3	0	3	6390.0495	-0.0002
3	2	2	2	1	1	6682.7022	0.0029
5	0	5	4	1	4	7107.0592	0.0007
5	1	5	4	0	4	7725.7432	-0.0008
6	1	5	5	2	4	7844.7205	-0.0012
4	2	3	3	1	2	8060.3459	-0.0011
3	3	1	2	2	0	8325.2907	-0.0006
3	3	0	2	2	1	8336.0725	-0.0004
6	0	6	5	1	5	8648.3190	0.0004
6	1	6	5	0	5	9066.0397	-0.0017
5	2	4	4	1	3	9388.5563	0.0014
4	3	2	3	2	1	9833.7863	-0.0007
4	3	1	3	2	2	9888.2866	0.0013
7	0	7	6	1	6	10157.1973	0.0002
7	1	7	6	0	6	10423.2146	-0.0001
6	2	5	5	1	4	10672.5416	0.0006
5	3	3	4	2	2	11304.3582	-0.0001
4	4	1	3	3	0	11356.0330	-0.0005
4	4	0	3	3	1	11356.7090	0.0000
5	3	2	4	2	3	11467.7616	-0.0016
8	0	8	7	1	7	11638.8466	0.0006
8	1	8	7	0	7	11800.6209	0.0005
7	2	6	6	1	5	11921.7976	-0.0013
6	3	4	5	2	3	12718.6423	0.0004
5	4	2	4	3	1	12885.9340	-0.0012
5	4	1	4	3	2	12890.7037	0.0003
6	3	3	5	2	4	13093.7119	0.0013
9	0	9	8	1	8	13100.5188	0.0006
8	2	7	7	1	6	13150.9171	0.0011
9	1	9	8	0	8	13195.5077	0.0004
7	3	5	6	2	4	14065.5807	-0.0005
5	5	1	4	4	0	14381.5888	0.0012
5	5	0	4	4	1	14381.6210	-0.0018
6	4	3	5	3	2	14407.7622	0.0001
6	4	2	5	3	3	14426.9230	0.0015
10	0	10	9	1	9	14548.7512	-0.0003
10	1	10	9	0	9	14603.0596	-0.0007
7	3	4	6	2	5	14791.4217	0.0006
7	4	4	6	3	3	15912.2465	-0.0006
6	5	2	5	4	1	15913.7381	0.0003
6	5	1	5	4	2	15914.0571	0.0001
7	4	3	6	3	4	15969.5916	-0.0013
11	1	11	10	0	10	16018.8495	-0.0001

Table S12: Observed transition frequencies (in MHz) for Ar-(E)-CHF^{13}CFCF$_3$

J'	K_a'	K_c'	J''	K_a''	K_c''	Observed	Obs - Calc
6	6	1	5	5	0	17406.4561	0.0005
6	6	0	5	5	1		

Table S13: Observed transition frequencies (in MHz) for Ar-(E)-$CHFCF^{13}CF_3$

J'	K_a'	K_c'	J''	K_a''	K_c''	Observed	Obs - Calc
4	1	4	3	0	3	6390.3430	0.0019
3	2	2	2	1	1	6686.3561	0.0006
5	0	5	4	1	4	7104.2702	0.0001
5	1	5	4	0	4	7725.6396	-0.0001
6	1	5	5	2	4	7837.9670	0.0007
4	2	3	3	1	2	8063.7220	-0.0012
3	3	1	2	2	0	8331.9874	0.0005
6	0	6	5	1	5	8645.5263	-0.0011
6	1	6	5	0	5	9065.4555	-0.0009
5	2	4	4	1	3	9391.6671	0.0000
4	3	2	3	2	1	9840.1943	-0.0003
4	3	1	3	2	2	9894.5216	-0.0010
7	0	7	6	1	6	10154.4068	0.0003
7	1	7	6	0	6	10422.0964	-0.0003
6	2	5	5	1	4	10675.3809	0.0000
5	3	3	4	2	2	11310.5779	-0.0005
4	4	1	3	3	0	11365.4563	0.0006
4	4	0	3	3	1	11366.1276	0.0003
5	3	2	4	2	3	11473.4808	-0.0010
8	0	8	7	1	7	11636.0128	0.0001
8	1	8	7	0	7	11798.9705	0.0004
7	2	6	6	1	5	11924.3131	-0.0001
6	3	4	5	2	3	12724.7874	0.0009
5	4	2	4	3	1	12895.0104	-0.0004
5	4	1	4	3	2	12899.7530	0.0011
9	0	9	8	1	8	13097.5807	-0.0008
6	3	3	5	2	4	13098.7465	-0.0001
8	2	7	7	1	6	13152.9897	-0.0006
9	1	9	8	0	8	13193.3642	0.0014
7	3	5	6	2	4	14071.7249	0.0009
5	5	1	4	4	0	14393.7488	0.0009
5	5	0	4	4	1	14393.7802	-0.0028
6	4	3	5	3	2	14416.5303	0.0016
6	4	2	5	3	3	14435.5800	-0.0002
10	0	10	9	1	9	14545.6539	-0.0001
10	1	10	9	0	9	14600.4704	-0.0006
7	3	4	6	2	5	14795.5254	0.0004
7	4	4	6	3	3	15920.7859	-0.0007
6	5	2	5	4	1	15925.5402	-0.0004
6	5	1	5	4	2	15925.8596	0.0024
7	4	3	6	3	4	15977.8109	-0.0017
11	1	11	10	0	10	16015.8614	0.0004

Table S13: Observed transition frequencies (in MHz) for Ar-(E)-$CHFCF^{13}CF_3$

J'	K_a'	K_c'	J''	K_a''	K_c''	Observed	Obs - Calc
6	6	1	5	5	0	17421.3533	-0.0002
6	6	0	5	5	1		

Table S14: Observed transition frequencies (in MHz) for Ar-(Z)-$CHFCFCF_3$

J'	K_a'	K_c'	J''	K_a''	K_c''	Observed	Obs - Calc
3	2	2	3	1	2	2019.3807	0.0010
4	2	2	4	1	3	2023.3304	-0.0026
2	0	2	1	1	1	2023.7456	0.0146
5	2	3	5	1	4	2027.0822	-0.0004
9	3	7	9	2	7	2060.8601	0.0005
3	2	1	3	1	2	2110.2223	-0.0004
1	1	1	0	0	0	2131.2550	-0.0068
3	1	2	2	2	0	2135.9129	0.0003
6	2	4	6	1	5	2160.0944	0.0048
2	2	1	2	1	1	2219.5912	0.0013
2	2	0	2	1	1	2238.1795	0.0025
1	1	0	0	0	0	2273.5003	0.0004
5	1	4	5	0	5	2286.7971	-0.0003
4	1	4	3	2	2	2376.2378	0.0046
7	2	5	7	1	6	2449.7036	-0.0006
8	3	6	8	2	6	2526.3623	0.0039
13	4	10	13	3	10	2593.8711	-0.0035
7	4	4	6	5	2	2594.4289	-0.0044
2	2	1	2	1	2	2646.2164	0.0071
2	2	0	2	1	2	2664.8012	0.0049
6	3	4	5	4	1	2776.3630	-0.0011
6	3	4	5	4	2	2777.2960	0.0003
5	2	4	4	3	1	2791.0942	0.0049
5	2	4	4	3	2	2801.6974	0.0053
3	2	2	3	1	3	2870.8007	-0.0003
8	2	6	8	1	7	2910.9025	0.0022
3	2	1	3	1	3	2961.6470	0.0030
7	3	5	7	2	5	2962.3940	0.0040
6	1	5	6	0	6	2975.7484	0.0041
3	0	3	2	1	1	3057.8107	0.0026
9	3	6	9	2	7	3062.9895	0.0056
8	3	5	8	2	6	3092.8298	-0.0018
5	1	5	4	2	2	3099.0464	-0.0078
4	2	3	4	1	4	3173.9153	0.0017
10	3	7	10	2	8	3195.4662	0.0045
12	4	9	12	3	9	3199.0391	0.0039
7	3	4	7	2	5	3244.2578	0.0016
6	3	4	6	2	4	3341.8873	0.0035
5	2	3	4	3	1	3351.9003	0.0018
5	1	5	4	2	3	3358.8301	0.0031
5	2	3	4	3	2	3362.5042	0.0029
2	1	2	1	0	1	3380.2958	0.0015
4	2	2	4	1	4	3433.6880	0.0017

Table S14: Observed transition frequencies (in MHz) for Ar-(Z)-$CHFCFCF_3$

J'	K_a'	K_c'	J''	K_a''	K_c''	Observed	Obs - Calc
6	3	3	6	2	4	3461.7220	0.0020
3	0	3	2	1	2	3484.4302	0.0027
11	3	8	11	2	9	3514.8696	0.0041
9	2	7	9	1	8	3538.1413	0.0028
5	2	4	5	1	5	3555.0242	0.0021
10	6	4	9	7	2	3612.6199	0.0118
5	3	3	5	2	3	3642.9441	0.0037
5	3	2	5	2	3	3684.4347	0.0037
6	1	6	5	2	3	3691.4778	0.0142
4	1	3	3	2	1	3695.7498	0.0061
7	1	6	7	0	7	3752.2153	0.0100
11	4	8	11	3	8	3786.5245	0.0233
2	1	1	1	0	1	3806.9156	0.0020
9	5	4	8	6	2	3826.6364	-0.0104
4	3	2	4	2	2	3852.3890	0.0048
4	3	1	4	2	2	3862.9912	0.0042
3	3	1	3	2	1	3972.8423	0.0048
3	3	0	3	2	1	3974.3740	0.0040
6	2	5	6	1	6	4010.2428	0.0054
6	2	5	5	3	2	4017.2638	-0.0061
12	3	9	12	2	10	4028.0666	0.0023
8	4	5	7	5	2	4030.2568	-0.0024
8	4	5	7	5	3	4030.6811	0.0073
13	4	9	13	3	10	4046.1595	0.0146
7	1	7	6	2	4	4052.1467	-0.0099
6	2	5	5	3	3	4058.7666	0.0061
3	3	1	3	2	2	4063.6844	0.0040
3	3	0	3	2	2	4065.2183	0.0054
4	0	4	3	1	2	4078.4161	0.0034
12	4	8	12	3	9	4095.1239	0.0036
4	3	2	4	2	3	4112.1605	0.0035
5	2	3	5	1	5	4115.8344	0.0031
4	3	1	4	2	3	4122.7641	0.0043
7	3	5	6	4	2	4173.4062	-0.0026
7	3	5	6	4	3	4178.0013	0.0047
5	3	3	5	2	4	4203.7541	0.0045
5	3	2	5	2	4	4245.2446	0.0043
6	1	6	5	2	4	4252.2733	0.0005
11	4	7	11	3	8	4292.2616	0.0019
10	2	8	10	1	9	4299.6623	0.0008
10	4	7	10	3	7	4321.5598	0.0027
6	3	4	6	2	5	4352.1810	0.0053
6	3	3	6	2	5	4472.0149	0.0031

Table S14: Observed transition frequencies (in MHz) for Ar-(Z)-$CHFCFCF_3$

J'	K_a'	K_c'	J''	K_a''	K_c''	Observed	Obs - Calc
7	2	6	7	1	7	4531.9986	0.0078
15	5	11	15	4	11	4555.8204	-0.0054
3	1	3	2	0	2	4565.8267	0.0041
8	1	7	8	0	8	4566.4098	0.0075
7	3	5	7	2	6	4568.5081	0.0024
10	4	6	10	3	7	4580.1201	0.0044
13	3	10	13	2	11	4718.6286	-0.0071
9	4	6	9	3	6	4774.2690	0.0054
7	3	4	7	2	6	4850.3749	0.0030
8	3	6	8	2	7	4859.8504	0.0048
9	4	5	9	3	6	4892.6671	0.0021
5	0	5	4	1	3	4924.3429	0.0042
4	0	4	3	1	3	4929.8350	0.0010
6	2	4	6	1	6	5020.5366	0.0074
6	2	4	5	3	2	5027.5684	0.0066
11	6	5	10	7	3	5049.1961	0.0077
7	1	7	6	2	5	5062.4540	0.0056
6	2	4	5	3	3	5069.0592	0.0068
8	2	7	8	1	8	5109.7683	0.0048
7	2	6	6	3	3	5122.4339	0.0065
8	4	5	8	3	5	5123.8302	0.0051
2	2	1	1	1	0	5144.2401	0.0029
2	2	0	1	1	0	5162.8296	0.0053
8	4	4	8	3	5	5171.5655	0.0047
5	1	4	4	2	2	5187.8070	0.0041
14	5	10	14	4	10	5225.7827	0.0050
9	3	7	9	2	8	5228.5773	0.0052
7	2	6	6	3	4	5242.2744	0.0108
10	5	5	9	6	3	5282.8558	-0.0074
2	2	1	1	1	1	5286.4799	0.0046
2	2	0	1	1	1	5305.0678	0.0055
7	4	4	7	3	4	5365.4225	0.0076
9	1	8	9	0	9	5378.8622	0.0125
7	4	3	7	3	4	5381.8662	0.0060
3	1	2	2	0	2	5417.2486	0.0047
8	3	5	8	2	7	5426.3233	0.0046
9	4	6	8	5	4	5471.7529	0.0072
6	4	3	6	3	3	5512.9419	0.0054
6	4	2	6	3	3	5517.5307	0.0065
8	3	6	7	4	4	5553.1228	0.0140
9	4	5	8	5	3	5588.3913	0.0072
5	4	2	5	3	2	5592.1558	0.0059
5	4	1	5	3	2	5593.0880	0.0066

Table S14: Observed transition frequencies (in MHz) for Ar-(Z)-$CHFCFCF_3$

J'	K_a'	K_c'	J''	K_a''	K_c''	Observed	Obs - Calc
6	0	6	5	1	4	5603.2476	0.0062
4	4	1	4	3	1	5629.3324	0.0146
6	4	3	6	3	4	5632.7763	0.0036
5	4	2	5	3	3	5633.6495	0.0090
5	4	1	5	3	3	5634.5805	0.0085
6	4	2	6	3	4	5637.3675	0.0070
4	4	0	4	3	2	5640.0303	0.0051
7	4	4	7	3	5	5647.2864	0.0054
7	4	3	7	3	5	5663.7308	0.0044
8	4	5	8	3	6	5690.3024	0.0042
4	1	4	3	0	3	5711.9007	0.0063
9	2	8	9	1	9	5731.5802	0.0116
8	4	4	8	3	6	5738.0408	0.0068
9	4	6	9	3	7	5776.3908	0.0028
8	1	8	7	2	6	5799.2937	0.0072
13	5	9	13	4	9	5812.7512	-0.0033
9	4	5	9	3	7	5894.7812	-0.0082
10	4	7	10	3	8	5919.4393	0.0050
8	2	7	7	3	4	6058.6868	0.0088
8	3	5	7	4	3	6103.1418	0.0052
11	4	8	11	3	9	6130.6870	-0.0002
7	2	5	7	1	7	6138.0966	-0.0099
7	0	7	6	1	5	6148.4387	-0.0045
10	1	9	10	0	10	6169.5795	-0.0020
10	4	6	10	3	8	6177.9926	-0.0004
11	3	9	11	2	10	6183.7383	-0.0100
9	3	6	9	2	8	6230.6952	-0.0013
12	5	8	12	4	8	6290.1733	-0.0003
5	0	5	4	1	4	6334.6866	-0.0053
8	2	7	7	3	5	6340.5430	-0.0012
10	2	9	10	1	10	6385.6188	-0.0037
12	5	7	12	4	8	6393.0105	-0.0023
3	2	2	2	1	1	6393.4659	-0.0034
12	4	9	12	3	10	6417.3025	-0.0166
9	1	9	8	2	7	6475.4659	0.0047
3	2	1	2	1	1	6484.3100	-0.0023
6	1	5	5	2	3	6551.9016	-0.0016
8	0	8	7	1	6	6608.8222	-0.0008
11	4	7	11	3	9	6636.4484	0.0027
11	5	7	11	4	7	6647.2546	-0.0039
11	5	6	11	4	7	6691.9600	-0.0036
7	2	5	6	3	3	6728.5443	0.0012
12	3	10	12	2	11	6753.1771	0.0020

Table S14: Observed transition frequencies (in MHz) for Ar-(Z)-$CHFCFCF_3$

J'	K_a'	K_c'	J''	K_a''	K_c''	Observed	Obs - Calc
13	4	10	13	3	11	6781.6504	-0.0038
3	2	2	2	1	2	6820.0829	-0.0057
9	3	7	8	4	4	6837.7485	0.0261
7	2	5	6	3	4	6848.3869	0.0076
5	1	5	4	0	4	6849.9355	-0.0053
9	3	7	8	4	5	6885.4628	0.0046
10	5	6	10	4	6	6892.5014	0.0028
10	4	7	9	5	5	6909.8259	-0.0183
10	5	5	10	4	6	6909.9945	0.0123
3	2	1	2	1	2	6910.9284	-0.0032
11	1	10	11	0	11	6935.1121	-0.0013
9	0	9	8	1	7	7035.1645	-0.0028
9	5	5	9	4	5	7048.6390	0.0005
9	5	4	9	4	5	7054.6542	-0.0040
11	2	10	11	1	11	7061.6576	-0.0038
10	1	10	9	2	8	7104.6162	0.0008
6	1	5	5	2	4	7112.7153	0.0030
4	1	3	3	0	3	7122.2456	-0.0021
8	5	4	8	4	4	7142.3621	-0.0026
8	5	3	8	4	4	7144.1321	0.0044
10	5	6	10	4	7	7151.0577	0.0005
11	5	7	11	4	8	7153.0126	-0.0045
10	4	6	9	5	4	7162.3879	0.0048
9	5	5	9	4	6	7167.0405	0.0006
10	5	5	10	4	7	7168.5397	-0.0011
9	5	4	9	4	6	7173.0602	0.0005
12	5	8	12	4	9	7186.2522	-0.0065
8	5	4	8	4	5	7190.1030	0.0025
8	5	3	8	4	5	7191.8641	0.0006
7	5	3	7	4	3	7196.2886	0.0008
7	5	2	7	4	3	7196.7041	0.0017
11	5	6	11	4	8	7197.7199	-0.0023
7	5	3	7	4	4	7212.7336	0.0004
7	5	2	7	4	4	7213.1498	0.0020
6	5	2	6	4	2	7226.2710	0.0144
6	5	1	6	4	3	7230.9033	-0.0111
5	5	1	5	4	1	7242.1955	0.0036
5	5	0	5	4	2	7243.1322	0.0023
13	5	9	13	4	10	7265.0357	0.0109
12	5	7	12	4	9	7289.1036	0.0057
9	2	8	8	3	6	7347.1818	-0.0025
8	2	6	8	1	8	7443.2472	-0.0035
10	0	10	9	1	8	7466.0205	0.0119

Table S14: Observed transition frequencies (in MHz) for Ar-(Z)-$CHFCFCF_3$

J'	K_a'	K_c'	J''	K_a''	K_c''	Observed	Obs - Calc
13	5	8	13	4	10	7480.7945	0.0111
4	2	3	3	1	2	7569.5258	-0.0007
12	1	11	12	0	12	7679.9522	0.0159
6	0	6	5	1	5	7691.9887	-0.0014
11	1	11	10	2	9	7699.6406	-0.0143
7	1	6	6	2	4	7740.5586	-0.0003
4	2	2	3	1	2	7829.2952	-0.0041
9	3	6	8	4	4	7839.8518	0.0050
15	6	10	15	5	10	7860.9273	-0.0166
11	0	11	10	1	9	7920.1844	-0.0024
6	1	6	5	0	5	8005.3426	-0.0009
10	3	8	9	4	6	8157.4497	-0.0001
14	6	9	14	5	9	8197.0950	0.0154
3	3	1	2	2	0	8218.9693	-0.0034
3	3	0	2	2	0	8220.5030	-0.0022
3	3	1	2	2	1	8237.5588	-0.0010
3	3	0	2	2	1	8239.0900	-0.0023
10	2	9	9	3	7	8261.6689	0.0032
12	1	12	11	2	10	8271.5349	0.0024
8	2	6	7	3	4	8392.1707	0.0054
4	2	3	3	1	3	8420.9429	-0.0049
13	6	8	13	5	8	8428.8637	-0.0093
11	3	8	11	2	10	8527.9252	-0.0092
12	6	7	12	5	7	8582.6097	-0.0100
12	6	6	12	5	7	8588.6782	0.0034
13	6	8	13	5	9	8644.6261	-0.0055
14	6	8	14	5	10	8650.8122	0.0085
13	6	7	13	5	9	8660.4081	0.0091
5	2	4	4	1	3	8677.4068	-0.0025
4	2	2	3	1	3	8680.7163	-0.0043
11	6	6	11	5	6	8682.6373	0.0003
12	6	7	12	5	8	8685.4758	0.0170
12	6	6	12	5	8	8691.5141	0.0001
8	1	7	7	2	5	8725.5208	-0.0004
11	6	6	11	5	7	8727.3449	0.0027
11	6	5	11	5	7	8729.4292	-0.0014
10	6	5	10	5	5	8747.3922	0.0006
10	6	4	10	5	5	8748.0247	0.0015
7	1	6	6	2	5	8750.8530	0.0022
10	6	5	10	5	6	8764.8753	0.0000
10	6	4	10	5	6	8765.5086	0.0018
9	6	4	9	5	4	8789.3438	0.0032
9	6	3	9	5	4	8789.4861	-0.0160

Table S14: Observed transition frequencies (in MHz) for Ar-(Z)-$CHFCFCF_3$

J'	K_a'	K_c'	J''	K_a''	K_c''	Observed	Obs - Calc
9	6	4	9	5	5	8795.3727	0.0123
9	6	3	9	5	5	8795.5180	-0.0038
8	6	3	8	5	3	8816.3481	-0.0146
8	6	2	8	5	4	8818.1642	0.0056
11	4	7	10	5	5	8821.3757	0.0124
13	1	13	12	2	11	8828.6990	0.0001
7	6	2	7	5	2	8833.3595	0.0039
7	6	1	7	5	3	8833.7775	0.0026
6	6	0	6	5	2	8843.4986	0.0064
6	6	1	6	5	1		
5	1	4	4	0	4	8938.6843	-0.0052
7	0	7	6	1	6	9008.8834	0.0006
11	2	10	10	3	8	9089.1444	0.0001
11	3	9	10	4	6	9094.9049	0.0052
7	1	7	6	0	6	9187.9898	-0.0008
5	2	3	4	1	3	9238.2197	0.0012
11	3	9	10	4	7	9353.4561	-0.0022
4	3	2	3	2	1	9571.4612	0.0004
4	3	1	3	2	1	9582.0638	0.0002
10	3	7	9	4	5	9636.9320	0.0064
4	3	2	3	2	2	9662.3005	-0.0032
4	3	1	3	2	2	9672.9021	-0.0044
6	2	5	5	1	4	9728.7849	0.0014
9	2	7	8	3	5	9948.4300	0.0064
12	3	10	11	4	7	9956.1430	0.0050
5	2	4	4	1	4	10087.7687	0.0061
10	1	9	9	2	7	10097.4608	0.0094
14	7	8	14	6	8	10218.1127	-0.0091
13	7	7	13	6	7	10289.1083	-0.0056
8	0	8	7	1	7	10297.2217	-0.0036
8	1	7	7	2	6	10331.6427	0.0059
12	7	6	12	6	6	10339.5306	0.0020
12	7	5	12	6	6	10339.7424	-0.0016
11	7	5	11	6	5	10375.4685	0.0109
11	7	4	11	6	6	10377.6012	-0.0031
8	1	8	7	0	7	10395.0779	-0.0023
10	7	4	10	6	4	10400.8301	0.0026
10	7	3	10	6	5	10401.4655	-0.0069
9	7	3	9	6	3	10418.2776	0.0017
9	7	2	9	6	4	10418.4433	0.0036
11	1	10	10	2	8	10555.6367	-0.0020
5	2	3	4	1	4	10648.5667	-0.0051
6	2	4	5	1	4	10739.0723	-0.0031

Table S14: Observed transition frequencies (in MHz) for Ar-(*Z*)-$CHFCFCF_3$

J'	K_a'	K_c'	J''	K_a''	K_c''	Observed	Obs - Calc
7	2	6	6	1	5	10744.2342	-0.0028
5	3	3	4	2	2	10857.8234	-0.0026
6	1	5	5	0	5	10865.7806	-0.0024
5	3	2	4	2	2	10899.3143	-0.0022
12	1	11	11	2	9	10936.1385	-0.0025
5	3	3	4	2	3	11117.5946	-0.0042
5	3	2	4	2	3	11159.0868	-0.0025
4	4	1	3	3	0	11236.9872	-0.0243
4	4	0	3	3	0	11237.1188	0.0029
4	4	1	3	3	1	11238.5384	-0.0056
4	4	0	3	3	1	11238.6697	0.0212
10	2	8	9	3	6	11334.1262	-0.0028
11	3	8	10	4	6	11439.0777	-0.0080
9	0	9	8	1	8	11567.5157	-0.0020
9	1	9	8	0	8	11619.2787	0.0021
8	2	7	7	1	6	11752.6442	0.0058
6	2	5	5	1	5	11817.5306	-0.0017
9	1	8	8	2	7	11836.6094	0.0055
13	8	6	13	7	6	11956.5856	0.0152
12	8	5	12	7	5	11980.2628	0.0045
12	8	4	12	7	6	11980.4762	-0.0023
11	8	4	11	7	4	11997.3536	-0.0235
11	8	3	11	7	5		
10	8	3	10	7	3	12009.1248	0.0014
10	8	2	10	7	4		
6	3	4	5	2	3	12053.8708	-0.0058
6	3	3	5	2	3	12173.7127	-0.0001
10	2	8	9	3	7	12336.2550	0.0016
7	2	5	6	1	5	12350.3524	-0.0004
11	2	9	10	3	7	12504.3300	-0.0058
6	3	4	5	2	4	12614.6823	-0.0035
5	4	2	4	3	1	12628.4761	-0.0034
5	4	1	4	3	1	12629.4082	-0.0027
5	4	2	4	3	2	12639.0809	-0.0013
5	4	1	4	3	2	12640.0126	-0.0012
6	3	3	5	2	4	12734.5192	-0.0028
9	2	8	8	1	7	12784.4404	-0.0026
10	0	10	9	1	9	12827.1520	0.0007
6	2	4	5	1	5	12827.8154	-0.0087
10	1	10	9	0	9	12853.8966	0.0056
7	1	6	6	0	6	12876.3956	0.0027
7	3	5	6	2	4	13152.6514	-0.0017
7	3	4	6	2	4	13434.5153	-0.0040

Table S14: Observed transition frequencies (in MHz) for Ar-(Z)-$CHFCFCF_3$

J'	K_a'	K_c'	J''	K_a''	K_c''	Observed	Obs - Calc
10	9	2	10	8	2	13593.4697	0.0053
7	2	6	6	1	6	13604.6777	0.0011
10	2	9	9	1	8	13860.6665	0.0027
6	4	3	5	3	2	14002.2166	-0.0017
6	4	2	5	3	2	14006.8011	-0.0050
6	4	3	5	3	3	14043.7096	0.0008
6	4	2	5	3	3	14048.2900	-0.0066
11	0	11	10	1	10	14080.7379	0.0025
8	2	6	7	1	6	14086.1238	-0.0018
11	1	11	10	0	10	14094.3139	0.0037
8	3	6	7	2	5	14162.7923	0.0125
7	3	5	6	2	5	14162.9406	-0.0044
5	5	0	4	4	0	14242.1690	-0.0180
5	5	1	4	4	1	14242.2981	0.0130
7	3	4	6	2	5	14444.8112	0.0000
8	3	5	7	2	5	14729.2499	-0.0031
8	1	7	7	0	7	14927.4302	-0.0005
11	2	10	10	1	9	14986.3816	-0.0086
9	3	7	8	2	6	15102.1015	-0.0133
12	0	12	11	1	11	15330.9301	-0.0050
12	1	12	11	0	11	15337.7357	-0.0003
7	4	4	6	3	3	15338.2158	0.0016
7	4	3	6	3	3	15354.6512	-0.0083
8	2	7	7	1	7	15441.0404	-0.0004
7	4	4	6	3	4	15458.0441	-0.0063
7	4	3	6	3	4	15474.4899	-0.0058
6	5	1	5	4	1	15640.0364	-0.0149
6	5	2	5	4	2	15640.9169	0.0042
8	3	6	7	2	6	15768.8911	-0.0045
12	1	11	11	2	10	15949.2036	-0.0063
9	2	7	8	1	7	15952.1514	-0.0041
10	3	8	9	2	7	15994.6830	-0.0143
9	3	6	8	2	6	16104.2365	-0.0026
12	2	11	11	1	10	16154.2399	-0.0072
13	0	13	12	1	12	16579.2124	0.0032
13	1	13	12	0	12	16582.6050	0.0226
8	4	5	7	3	4	16608.8210	-0.0009
8	4	4	7	3	4	16656.5531	-0.0046
8	4	5	7	3	5	16890.6837	-0.0043
8	4	4	7	3	5	16938.4123	-0.0115
9	1	8	8	0	8	16980.4250	0.0056
7	5	3	6	4	2	17033.4381	0.0150
7	5	2	6	4	2	17033.8346	-0.0030

Table S14: Observed transition frequencies (in MHz) for Ar-(Z)-$CHFCFCF_3$

J'	K_a'	K_c'	J''	K_a''	K_c''	Observed	Obs - Calc
7	5	3	6	4	3	17038.0092	-0.0016
7	5	2	6	4	3	17038.4270	0.0016
13	1	12	12	2	11	17237.6905	-0.0129
6	6	0	5	5	0	17241.3085	-0.0048
6	6	1	5	5	1		
9	2	8	8	1	8	17316.7904	-0.0030
13	2	12	12	1	11	17352.3404	-0.0261
9	3	7	8	2	7	17435.5995	-0.0025
10	3	7	9	2	7	17592.5672	-0.0075
9	4	6	8	3	5	17785.6678	-0.0034
14	0	14	13	1	13	17826.3182	-0.0035
14	1	14	13	0	13	17827.9994	0.0175
9	4	5	8	3	5	17904.0700	-0.0026
10	2	8	9	1	8	17935.2492	-0.0023

Table S15: Observed transition frequencies (in MHz) for Ar-(*Z*)-$^{13}CHFCFCF_3$

J'	K_a'	K_c'	J''	K_a''	K_c''	Observed	Obs - Calc
3	2	2	2	1	2	6782.8905	0.0019
5	1	5	4	0	4	6821.5161	-0.0005
4	1	3	3	0	3	7110.3930	-0.0017
6	1	6	5	0	5	7975.0936	-0.0005
3	3	0	2	2	0	8159.3658	0.0001
3	3	1	2	2	1	8176.9593	0.0000
4	2	3	3	1	3	8383.4625	0.0019
5	1	4	4	0	4	8929.4607	0.0018
7	1	7	6	0	6	9156.2149	0.0003
5	2	3	4	1	3	9202.9399	-0.0022
4	3	2	3	2	1	9506.5900	-0.0018
4	3	1	3	2	1	9517.8115	-0.0001
4	3	2	3	2	2	9600.4147	-0.0011
4	3	1	3	2	2	9611.6351	-0.0004
8	1	8	7	0	7	10361.4711	0.0005
6	2	4	5	1	4	10708.5301	-0.0026
5	3	3	4	2	2	10787.4655	0.0004
5	3	2	4	2	2	10831.3275	0.0000
6	1	5	5	0	5	10859.0971	0.0037
5	3	3	4	2	3	11055.2458	-0.0020
5	3	2	4	2	3	11099.1094	-0.0007
4	4	0	3	3	0	11152.3538	0.0007
4	4	1	3	3	1	11153.8624	0.0003
9	1	9	8	0	8	11583.3127	-0.0008
6	2	5	5	1	5	11780.7572	0.0007
6	3	4	5	2	3	11976.2837	0.0013
6	3	3	5	2	3	12102.7440	0.0002
7	2	5	6	1	5	12326.4370	0.0003
5	4	2	4	3	1	12541.2253	-0.0001
5	4	1	4	3	1	12542.2354	0.0005
5	4	2	4	3	2	12552.4459	0.0007
6	3	4	5	2	4	12552.8563	-0.0013
5	4	1	4	3	2	12553.4558	0.0011
6	3	3	5	2	4	12679.3189	-0.0002
10	1	10	9	0	9	12815.1043	0.0002
7	1	6	6	0	6	12870.1016	-0.0002
7	3	4	6	2	4	13363.5287	0.0023
7	2	6	6	1	6	13568.4125	-0.0024
6	4	3	5	3	2	13911.5678	-0.0001
6	4	2	5	3	2	13916.5377	0.0004
6	4	3	5	3	3	13955.4308	0.0006
6	4	2	5	3	3	13960.4008	0.0011
8	2	6	7	1	6	14070.6045	-0.0002

Table S15: Observed transition frequencies (in MHz) for Ar-(Z)-$^{13}CHFCFCF_3$

J'	K_a'	K_c'	J''	K_a''	K_c''	Observed	Obs - Calc
5	5	0	4	4	0	14133.6615	0.0000
5	5	1	4	4	1	14133.7668	-0.0009
8	3	5	7	2	5	14662.6848	0.0023
7	4	3	6	3	3	15260.0901	-0.0006
7	4	4	6	3	4	15368.7548	0.0002
6	5	1	5	4	1	15529.3360	-0.0007
6	5	2	5	4	2	15530.2680	-0.0006
8	3	6	7	2	6	15711.0255	-0.0017
8	4	4	7	3	4	16556.3566	-0.0002
8	4	5	7	3	5	16801.3622	-0.0003
7	5	2	6	4	2	16920.7382	0.0004
7	5	3	6	4	3	16925.2471	-0.0003
6	6	0	5	5	0	17109.0675	0.0004
6	6	1	5	5	1		

Table S16: Observed transition frequencies (in MHz) for Ar-(Z)-$CHF^{13}CFCF_3$

J'	K_a'	K_c'	J''	K_a''	K_c''	Observed	Obs - Calc
3	2	2	2	1	2	6813.7436	0.0023
5	1	5	4	0	4	6844.7530	0.0003
4	1	3	3	0	3	7119.2217	0.0011
6	1	6	5	0	5	7999.6758	-0.0004
3	3	0	2	2	0	8210.6890	0.0011
3	3	1	2	2	1	8227.8107	0.0008
5	1	4	4	0	4	8935.6501	0.0002
7	1	7	6	0	6	9181.8818	-0.0008
5	2	3	4	1	3	9231.6177	-0.0014
4	3	2	3	2	1	9560.8687	-0.0009
4	3	2	3	2	2	9652.0846	-0.0016
4	3	1	3	2	2	9662.7669	-0.0002
5	2	4	4	1	4	10080.7422	0.0011
8	1	8	7	0	7	10388.4891	-0.0005
5	3	3	4	2	2	10846.2405	0.0001
6	1	5	5	0	5	10862.7100	0.0009
5	3	2	4	2	2	10888.0312	-0.0003
5	3	3	4	2	3	11107.0161	-0.0012
5	3	2	4	2	3	11148.8069	-0.0015
4	4	0	3	3	0	11223.5446	-0.0003
4	4	1	3	3	1	11224.9829	-0.0002
9	1	9	8	0	8	11612.1346	0.0019
6	2	5	5	1	5	11810.2301	-0.0022
6	3	4	5	2	3	12041.0838	0.0023
6	3	3	5	2	3	12161.7588	-0.0003
7	2	5	6	1	5	12344.6414	-0.0006
6	3	4	5	2	4	12603.8634	-0.0012
5	4	1	4	3	1	12615.2263	0.0000
5	4	2	4	3	2	12624.9657	-0.0002
5	4	1	4	3	2	12625.9063	-0.0010
6	3	3	5	2	4	12724.5439	0.0017
10	1	10	9	0	9	12846.1292	-0.0009
7	1	6	6	0	6	12872.9971	0.0013
7	3	4	6	2	4	13422.2871	0.0007
7	2	6	6	1	6	13597.0912	-0.0016
6	4	3	5	3	2	13987.2865	0.0003
6	4	2	5	3	2	13991.9223	0.0001
6	4	3	5	3	3	14029.0779	0.0005
6	4	2	5	3	3	14033.7144	0.0010
8	2	6	7	1	6	14081.1977	-0.0009
7	3	5	6	2	5	14152.0114	-0.0004
5	5	0	4	4	0	14224.8541	0.0005
5	5	1	4	4	1	14224.9520	-0.0007

Table S16: Observed transition frequencies (in MHz) for Ar-(Z)-$CHF^{13}CFCF_3$

J'	K_a'	K_c'	J''	K_a''	K_c''	Observed	Obs - Calc
8	3	5	7	2	5	14717.3286	0.0011
7	4	3	6	3	3	15338.9232	-0.0004
7	4	4	6	3	4	15442.9854	0.0009
6	5	1	5	4	1	15622.1255	-0.0008
6	5	2	5	4	2	15622.9986	0.0019
8	3	6	7	2	6	15757.9644	0.0016
8	4	4	7	3	4	16639.7990	-0.0007
8	4	5	7	3	5	16875.3148	-0.0013
7	5	2	6	4	2	17015.2966	0.0002
7	5	3	6	4	3	17019.5117	-0.0005
6	6	0	5	5	0	17220.2335	-0.0001
6	6	1	5	5	1		

Table S17: Observed transition frequencies (in MHz) for Ar-(Z)-CHFCF^{13}CF$_3$

J'	K_a'	K_c'	J''	K_a''	K_c''	Observed	Obs - Calc
3	2	2	2	1	2	6810.3056	-0.0006
5	1	5	4	0	4	6833.3365	0.0000
6	1	6	5	0	5	7984.7138	-0.0010
3	3	0	2	2	0	8214.9689	0.0000
3	3	1	2	2	1	8231.8250	0.0015
4	2	3	3	1	3	8405.6774	0.0002
5	1	4	4	0	4	8911.5597	0.0005
7	1	7	6	0	6	9163.1762	0.0001
5	2	3	4	1	3	9218.4898	-0.0006
4	3	2	3	2	1	9562.0392	-0.0011
4	3	1	3	2	1	9572.4380	0.0006
4	3	2	3	2	2	9651.7852	-0.0007
5	2	4	4	1	4	10066.7075	0.0014
8	1	8	7	0	7	10366.1131	0.0004
6	2	4	5	1	4	10712.5604	-0.0015
6	1	5	5	0	5	10831.5460	0.0023
5	3	3	4	2	2	10845.2016	0.0001
5	3	2	4	2	2	10885.8985	-0.0002
5	3	3	4	2	3	11101.9835	-0.0003
5	3	2	4	2	3	11142.6798	-0.0012
4	4	0	3	3	0	11230.1206	-0.0004
4	4	1	3	3	1	11231.5222	0.0003
9	1	9	8	0	8	11586.2752	-0.0001
6	2	5	5	1	5	11790.4540	-0.0012
6	3	4	5	2	3	12038.7892	0.0016
6	3	3	5	2	3	12156.3940	0.0004
7	2	5	6	1	5	12316.2286	0.0009
6	3	4	5	2	4	12593.5818	-0.0009
5	4	2	4	3	1	12617.1452	0.0001
5	4	1	4	3	1	12618.0520	0.0002
5	4	2	4	3	2	12627.5428	0.0006
6	3	3	5	2	4	12711.1894	0.0006
10	1	10	9	0	9	12816.9782	-0.0001
7	1	6	6	0	6	12835.4842	-0.0002
7	3	4	6	2	4	13412.4578	-0.0011
7	2	6	6	1	6	13571.4441	-0.0009
6	4	3	5	3	2	13986.8547	0.0001
6	4	2	5	3	2	13991.3206	-0.0006
6	4	3	5	3	3	14027.5519	0.0001
6	4	2	5	3	3	14032.0180	-0.0005
7	3	5	6	2	5	14135.9319	0.0000
5	5	0	4	4	0	14233.8017	0.0005
5	5	1	4	4	1	14233.8951	-0.0016

Table S17: Observed transition frequencies (in MHz) for Ar-(Z)-$CHFCF^{13}CF_3$

J'	K_a'	K_c'	J''	K_a''	K_c''	Observed	Obs - Calc
8	3	5	7	2	5	14701.0335	0.0014
7	4	3	6	3	3	15335.4833	0.0022
7	4	4	6	3	4	15437.0732	0.0012
6	5	1	5	4	1	15627.2324	0.0003
6	5	2	5	4	2	15628.0717	0.0005
8	3	6	7	2	6	15735.5680	-0.0018
8	4	4	7	3	4	16634.2045	0.0003
8	4	5	7	3	5	16864.5543	-0.0003
7	5	2	6	4	2	17016.6552	-0.0007
7	5	3	6	4	3	17020.7209	-0.0010
6	6	0	5	5	0	17231.5371	0.0005
6	6	1	5	5	1		
